# Empirical networks for localized COVID-19 interventions using WiFi infrastructure at university campuses

**DOI:** 10.3389/fdgth.2023.1060828

**Published:** 2023-05-16

**Authors:** Vedant Das Swain, Jiajia Xie, Maanit Madan, Sonia Sargolzaei, James Cai, Munmun De Choudhury, Gregory D. Abowd, Lauren N. Steimle, B. Aditya Prakash

**Affiliations:** ^1^ College of Computing, Georgia Institute of Technology, Atlanta, GA, United States; ^2^ H. Milton Stewart School of Industrial and Systems Engineering, Georgia Institute of Technology, Atlanta, GA, United States; ^3^ Department of Computer Science, Brown University, Providence, RI, United States; ^4^ College of Engineering, Northeastern University, Boston, MA, United States

**Keywords:** COVID-19, mobility, modeling, policy, non-pharmaceutical intervention, WiFi

## Abstract

Infectious diseases, like COVID-19, pose serious challenges to university campuses, which typically adopt closure as a non-pharmaceutical intervention to control spread and ensure a gradual return to normalcy. Intervention policies, such as remote instruction (RI) where large classes are offered online, reduce potential contact but also have broad side-effects on campus by hampering the local economy, students’ learning outcomes, and community wellbeing. In this paper, we demonstrate that university policymakers can mitigate these tradeoffs by leveraging anonymized data from their WiFi infrastructure to learn community mobility—a methodology we refer to as *WiFi mobility models* (WiMob). This approach enables policymakers to explore more granular policies like localized closures (LC). WiMob can construct contact networks that capture behavior in various spaces, highlighting new potential transmission pathways and temporal variation in contact behavior. Additionally, WiMob enables us to design LC policies that close super-spreader locations on campus. By simulating disease spread with contact networks from WiMob, we find that LC maintains the same reduction in cumulative infections as RI while showing greater reduction in peak infections and internal transmission. Moreover, LC reduces campus burden by closing fewer locations, forcing fewer students into completely online schedules, and requiring no additional isolation. WiMob can empower universities to conceive and assess a variety of closure policies to prevent future outbreaks.

## 1. Introduction

University campuses are often hotspots for infectious disease outbreaks and hence are targeted for interventions. In the wake of the Coronavirus Disease (COVID-19) (1), the U.S. witnessed more than half a million cases at universities (2). On the event of infectious disease outbreaks, colleges must make crucial decisions to ensure continuity of operations in safe way (3,4). Controlling the disease at universities can be pivotal to securing the surrounding environment (5). To reduce on-campus infections and the likelihood of superspreading events, a recommended form of non-pharmaceutical intervention (NPI) is partial closure of the campus (6).

During COVID-19, advancement in teleconferencing technology equips universities to continue operations by adopting a form of campus closure that relies on remote instruction (RI) (7). As a consequence, the campus community has fewer opportunities to visit spaces, such as classrooms, to congregate and risk transmission (8,9). One common approach campuses consider to design RI policies is to use enrollment data (En) to assume contact and therefore, offer large classes online while other classes remain in person (10,11). In fact, during COVID-19, 44% colleges and universities in the U.S., primarily offered instruction online (12). However, these policies can still have broad, negative, and indiscriminate impact on the community by forcing students into completely remote course schedules. Such policies can have adverse effect on learning outcomes (13), where students can lose close to 7 months of education (14). Additionally, RI can disincentivize students to stay on campus and thus, universities incur losses in auxiliary revenue (e.g., boarding, parking, dining, etc.) (15,16), with universities standing to lose up to $50 million because of unused services (17). Even the local population unaffiliated with the university takes sustains losses to business due to university closures (18,19). Furthermore, with socioeconomic disparities and heterogeneous household contexts, the demands of remote instruction can lead to added anxiety and stress among students (20,21). Relying on RI, university campuses struggle to balance community health with the demands of learning, economy, and broad wellbeing (22). Instead, there is a need for a more versatile approach to design closure policies that empowers policymakers to accurately assess impact of closure interventions and model more data-driven targeted intervention strategies.

**Figure 1:**
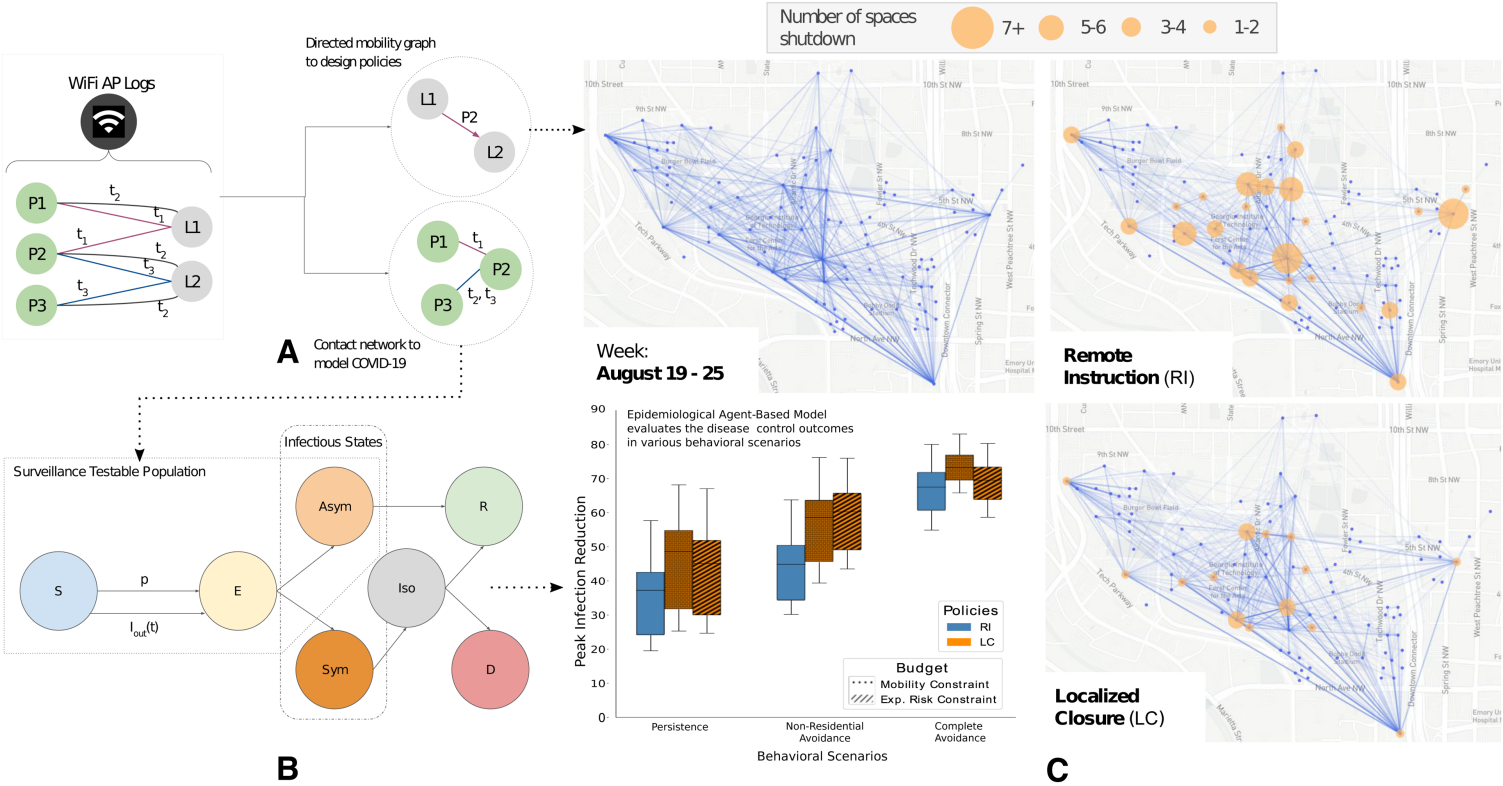
The WiFi mobility models (WiMob) methodology uses anonymized network logs to model campus mobility for localized closures (LC) (**A**) WiFi network logs reflect timestamps when people’s devices associate with access points (APs) on campus. WiMob mines these logs to characterize mobility as a bipartite graph that describes people (e.g., P1, P2) visiting campus locations (e.g., L1, L2) during different times (e.g., $t_{1}$, $t_{2}$). Since people’s devices can proxy their presence, we estimate collocation (e.g., P1 and P2 were collocated at L1 at $t_{1}$), and movement (P2 dwelled at L1 and then at L2). (**B**) We use the collocation network construct a SEIR–based epidemiological ABM, calibrated to Fall 2020 incidence of COVID-19 (*C*) WiMob highlights mobility behavior to evaluate and inform policy. (**C**)–*top-left:* Mobility on campus between the top 100 most frequented locations on the GT campus in the Fall semester of 2019. Edges only connect points of significant dwelling and thus do not represent pedestrian routes. (**C**)–*top-right:* RI is a form of broad closure which affects a large number of students and locations. (**C**)–*bottom-right:* By contrast, we propose to use WiMob to parsimoniously identify a small set of spreader locations within buildings and design LC policies. (**C**)–*bottom-left:* We use our epidemiological ABM to evaluate these policies under different budgetary constraints and various behavioral scenarios (Persistence, Non-Residential Avoidance, Complete Avoidance). Our study shows that LC policies provide equal or better control on the disease spread, and yet minimize the burden on campus compared to RI.

This paper showcases a new approach that universities can take to design closure policies by leveraging data from their existing WiFi infrastructure. Our methodology, *WiFi mobility models* (WiMob), involves constructing anonymized mobility networks of campus (Figure 1A), which helps determine extended periods of collocation—or “proximate contact” (23)—between individuals to describe contact networks on campus. Particularly, WiMob enables a more expressive toolkit for university policymakers that represents contact longitudinally and allows them to assess closure at the granularity of a room, suite, or hall. Thus, it lends itself to the design of targeted interventions that focus on localized closures (LC). We demonstrate the utility of WiMob with data collected over two years, of approximately 40,000 anonymous occupants and visitors of the Georgia Institute of Technology (GT), a large urban campus in the U.S.—including about 16,000 undergraduate students, 9,000 graduate students, and 7,600 staff members. In general, on comparing WiMob to En as an approach to model contact, we find that WiMob captures contact behavior at a community scale for a variety of campus spaces, describes temporal variations in contact, and provides a better estimate of local context by being aware of occupancy and the non-student population. Using WiMob also reveals that En overestimates the impact of RI on reducing contact on campus. Hence, we propose a less burdensome alternative to RI, by deriving more targeted LC policies based on WiMob (Figure 1) (indeed En is too coarse-grained for designing targeted LC policies).

We further exhibit that LC presents better disease control outcomes than RI by constructing and simulating an agent-based epidemiological model (ABM) over the people–people contact networks (Figure 1B) derived from the collocation identified with WiMob (Figure 1A). Our ABM was calibrated with GT on-campus COVID-19 cases from the Fall semester of 2020 (24) and infection rates from Fulton County (25). To compare the effect of interventions, we construct a counterfactual semester—that is unaltered by other policy–induced behaviors of 2020—by leveraging WiFi data from Fall 2019 to determine the contact structure of the simulation. We assess the effectiveness of closure NPIs (Figure 1C) by simulating COVID-19 under various behavioral scenarios. We find LC is comparable to RI in controlling total infections but more effective at reducing the peak infections and internal transmission. Additionally, LC targets fewer locations, forces fewer students into fully online schedules, and does not isolate any more people than RI—illustrating that WiMob can help universities devise highly-specific closure policies, like LC, which can contain disease spread and mitigate campus disruption in comparison to RI policies.

Our methodology also promises other advantages. Mobility generally has been used to dynamically model disease spread of influenza (26), rubella (27) and COVID-19 (9,28) showing the effectiveness of mobility restrictions at a regional–, or city–level (29–33). These studies typically rely on cell tower localization or aggregating GPS information from mobile phones (34). Neither of these data sources is easy to access for university campuses. At the same time, studies to infer campus mobility networks have relied on accessing user devices with specialized data logging applications (e.g., contact tracing mobile apps) (35–38), but these approaches are typically constrained for disease modeling because they require mass adoption to represent the entire community and continuous maintenance of software is needed to capture longitudinal behavior changes. In contrast, our work repurposes already existing managed WiFi networks to model mobility, which provides room level granularity for mobility (39–42) and consequently indicates proximate contact (23). Much like En, universities internally archive such data over a long term for other purposes and do not need to install any additional surveillance infrastructure to access it. Prior work has repurposed such data for campuses of size 10,000–50,000 in different locations including Singapore, the U.K., and the U.S (39,43). With the appropriate privacy considerations, a university can obtain such data at a low cost, continuously and unobtrusively. The possibility of pandemic still looms large in the future (44,45). As campuses prepare for upcoming semestera and unforeseen contagious diseases of tomorrow, WiMob presents an attractive and practical method to inform better public health policies.

## 2. Materials and methods

This section summarizes (i) the data used to derive contact networks and policies, and (ii) the dynamics of our simulation and calibration approach. Additional information for every subsection is present in Supplementary Material, Methods.

### 2.1. WiFi mobility

Here we describe the data for our methodology, WiFi mobility models (WiMob) and the process to yield Localized (LC) policies.

#### 2.1.1. Data use and access

The IT management facility at Georgia Tech (GT) accumulates WiFi access point logs over time. This is common in most universities with managed WiFi infrastructure. We actively collaborated with IT management to define safety and security safeguards that allow us to obtain a de-identified version of these raw logs. Before accessing the data we established a data-use agreement and an ethics protocol that was approved by the Institutional Review Board (IRB) at Georgia Institute of Technology (Protocol H20208). For the WiFi data, we were provided access to logs from Fall 2019 and Fall 2020. We processed these logs to characterize mobility (WiMob) and it encompasses all 40,000 unique visitors that connected to the network via 6,959 different access points (41). The logs did not contain any personally identifiable information and locations are also coded. The logs indicated the WiFi access point (AP) a device associates with and can therefore be used to infer dwelling locations of users across the entire campus. This is limited to indoor spaces where APs are located and the scope of this localization is at the granularity of a room or suite (39,43)). For En we only used aggregate insights for enrollment, which were derived from course registration transcripts. Note, we did not cross-identify any students. We used publicly accessible course schedules to approximate schedules of de-identified nodes and infer if they were students or staff, and non-residential or residential. We elaborate on our data in Supplementary Material, Data.

**Note.** Like most universities, GT’s managed WiFi network is not equipped with any Real-Time Location System (RTLS) (46,47). RTLS systems use Received Signal Strength Indicator (RSSI) values from multiple neighboring APs to provide high precise localization of individuals in terms of time and space. However, deploying such systems requires surveying the entire network. Additionally, precision localization raises more privacy concerns. These factors together make it challenging for universities to justify the deployment of RTLS, unlike small retail settings that can monetize RTLS insights directly (e.g., insights on footfall can be tied to improving revenue).

#### 2.1.2. Contact and movement networks

WiMob leverages the logs to create bipartite graphs $K_{t}$, for each day t, which connect P users to L access point locations (Figure 1A). Any edge, $\{p,l{\}}_{i}$ indicates the ith instance when a p was dwelling at l. These edges describe the time period of dwelling. Subsequently, by comparing all edges in $K_{t}$ we can infer if different individuals are collocated near an AP to create a contact network, $G_{t}$, for each day t—between any collocated $p_{i},p_{j}\in P$. These networks define the contact structure for an epidemiological agent-based model at every time-step. Similarly, by inspecting the sequence of dwelling locations for any p in graph K, we compute a mobility network, $H_{t}$—between locations l∈L. In our approach, we considered collocation as a form of *proximate contact*—people in the same room—and therefore established collocation only when this occurred for “an extended period” (23). By varying this threshold between 30 and 40 minutes we found the contact networks to be structurally similar as their clustering coefficients (over the semester) were highly correlated (r=0.97). In our experiments, we used the 40 minute threshold as it was more computationally less expensive. We provide more details of our approach in Supplementary Material, Data Processing and in Supplementary Material, Modeling Contact and Movement.

#### 2.1.3. Modeling policies

We compared the disease outcomes and burdens of 2 policies, Remote Instruction (RI) and Localized Closure (LC), both of which are modeled with WiMob.

**Remote Instruction (RI):** The status quo for data-driven policies offers strictly online instruction for large class enrollment, while continuing the other classes in person. When using En to model contacts, we implemented RI by removing connections between students who were only in contact through courses of size ≥30. When using WiMob to model contacts, we removed connections between students if they were only connected because of collocations during scheduled lectures of such courses.
**Localized Closure (LC):** We identified rooms–level spaces that are highly central location nodes in the network. We removed contacts between people who are only connected because of collocating at these locations.

To further elaborate, for RI we inferred enrollment size of each course in Fall 2019 by determining the number of unique individuals that visited lecture locations during scheduled times. After the first week, we applied the RI by removing all visiting edges in $K_{t}$ for any $l_{c}\in L_{\text{RI }}$ if visits were during lecture times of course c with an enrollment ≥30. This helped create counterfactual contact networks $G_{t}^{\prime }$. The removal of edges from K described the mobility budget of RI and the structure of $G_{t}^{\prime }$ indicated the risk of exposure budget. We designed LC with these budgets by identifying locations for closure ($L_{\text{LC }}$) with different algorithms, such as *PageRank (48), Eigenvector Centrality (49), Load Centrality (50), and Betweenness Centrality (51)*. When a location was closed, we removed all edges in $K_{t}$ connected to any $l_{x}\in L_{\text{LC }}$. We aggregated the movement graph $H_{t}$ over a week and apply the algorithms to identify locations. Subsequently, we identified the number of top-ranked locations to remove such that the resultant counterfactual contact network $G_{t}^{\prime \prime }$ has is within 1% of the budget. More details for closure policies have been expanded in Supplementary Material, Identifying Locations for Closure.

To make the comparisons between the closure policies, we established fixed budgets to design LC based on the resource utilization on RI. We considered 2 kinds of budgets, (i) mobility reduction—to depict space use on campus, and (ii) risk of exposure—to reflect testing capacity. Also note, response to closure policies can lead to unpredictable side-effects in campus behavior, particularly when a student’s schedule is entirely online. Therefore, we design policies within three behavioral scenarios (each with a varying budget):

*[S1] Persistence*: Irrespective of the locations closed or classes restricted, individuals continue their other visiting behaviors.
*[S2] Non-Residential Avoidance*: Non-residential students stop all visits to campus if they enrolled in at least 3 courses and the policy forces their entire academic schedule online.
*[S3] Complete Avoidance*: Same as S2, but even residential students avoid campus based on their schedule.

The budgets varied for different behavioral scenarios and we only compared policies within the same scenario. Similar to other works that model closure (11,52), we assume that when a location is shutdown, the individuals who ought to have visited that location isolated during the time. This is further elaborated in Supplementary Material, Modeling Policies and Scenarios.

### 2.2. Disease simulation

Here we summarize our epidemiological model and calibration process.

#### 2.2.1. Agent-based model

We constructed an agent-based model (ABM) that captures the spread of COVID-19 between individuals active on campus. This ABM leveraged the contact networks produced by WiMob. The simulation iterated a time-step each day with the underlying contact networks i.e., $G_{t}$ for no interventions, $G_{t}^{\prime }$ for RI, and $G_{t}^{\prime \prime }$ for LC. Each agent in our ABM follows a modified version of *susceptible–exposed–infectious–removed* (SEIR) template that disambiguates the *infectious* compartment into *asymptomatic* and *symptomatic*. New infections were introduced to the model either externally or internally. External transmission arose because individuals could contract the virus outside campus and bring the infection back for local spread (7,53). We adopted data of positive cases from Fulton county (25) with a scaling factor α to estimate the probability that a *susceptible* individual, who is active on campus, was infected from interactions that take place outside campus. Internal transmissions are determined by p, as the probability of *susceptible* individuals in contact with an *infectious* one. We calibrated the parameters related to disease transmission by training and validating our models on the positivity rate reported by GT surveillance testing (24). Supplementary Material, Agent-Based Model details the disease progression and describes the various parameters.

#### 2.2.2. Calibration

In our study, we estimated three key parameters: (i) infectious individuals at day 0, (ii) transmission probability between infectious and susceptible individuals, and (iii) the probability of infection transmission from contacts outside the network. We estimated the range of optimal parameters for disease transmission by minimizing the root means square error (r.m.s.e) between the Georgia Tech surveillance testing positive rates (24,54) and the observed positivity rate of the model every week—percentage of new *asymptomatic* cases out of the total testable population. The surveillance testing conducted by Georgia Tech was designed for detecting individuals who contracted Covid-19 without showing Flu-like symptoms within the community (54). We calibrated the model on the positivity rates on the first 5 weeks of Fall 2020. To attain a point estimation of the optimal parameters, we fitted the model to predict trends in the remaining weeks by running a numerical optimization algorithm, Nelder-Mead (55). To account for quantitative uncertainty, we estimated a range of parameters, within 40% of optimum r.m.s.e (30). For other model parameters, we adopted values proposed by previous studies on similar populations (56–58). Table 1 shows a full list of our parameters.

**Table 1:** Model parameters of the ABM.

Parameter	Definition	Value	Std	Source
p	Transmission probability: For any edge between a *susceptible* and *infectious* individual in the contact network, p is the probability that the *susceptible* person will enter into the *exposed* state. This only dictates internal transmission	0.034	0.007	Calibration
α	Scaling factor of the normalized confirmed cases in the surrounding county (S1). This is the parameter for us to generate $I_{out}(t)$	0.032	0.0032	Calibration
$I_{0}$	Proportion of population that is *asymptomatic* at day 0	0.012	0.0009	Calibration
$p_{S}$	Probability of *exposed* persons becoming symptomatic	0.66	-	(56)
$\Delta_{S}$	Incubation period (days) since the first day of exposure	5	-	(56)
$\Delta_{\text{Asym}\to R}$	Asymptomatic duration (days); it is the time taken for an *asymptomatic* person to recover since the first day of exposure	7	-	(56)
$\Delta_{I}$, $\sigma_{I}$	Time of an *symptomatic* entering *isolated* since the first day of exposure of a *symptomatic* person	8	2	(57)
$\Delta_{R}$, $\sigma_{R}$	Time for recovery for a *symptomatic*, since the first day of exposure	12	2	(58)
$p_{D}$	Death rate under isolation	0.0006	-	(58)

The variables p, α, and $I_{0}$ are estimated by calibrating the simulation model on the first 5 weeks of positivity rates provided by GT surveillance for Fall 2020, while incorporating external cases from Fulton County. These parameters were found by validating the ABM on the remaining weeks of Fall 2020. Supplementary Material, Calibration provides additional details.

Note that our calibration characterized latent factors associated with pandemic-related cautious behaviors, including the relationship with external transmission. And these factors could be related to “county characteristics, partisanship, media consumption, and racial and ethnic composition” (8). To account for the effect of these varying latent factors on disease outcomes, we performed additional calibrations for hypothetical variations in disease spread. For these analyses we kept the GT mobility behavior constant while calibrating the model on different time periods of surveillance testing and on positivity rates of different U.S. universities—University of Illinois at Urbana-Champaign (59) and University of California (60), Berkeley. We evaluated RI and LC on these variations and describe the design of these complementary experiments in Supplementary Material, Sensitivity Analyses. See Supplementary Material, Calibration for details on the calibration process and results of all variations are in Table S3.

## 3. Results

We present two sets of analyses in our work. The first set contrasts structural characteristics of contact networks described by WiMob with current practices that use enrollment data (En). In the next set, we used WiMob to build an epidemiological model (an agent-based model over the contact networks, referred to as ABM) and analyze the remote instruction (RI) and localized closure (LC) interventions in terms of their differences in dynamic disease-control outcomes and burdens to campus.

Note, throughout the paper we use the small-caps to denote different methodologies to model contact (WiMob and En) and sans-serif to denote different intervention strategies (RI and LC).

### 3.1. WiMob provides local, holistic and dynamic structural insights for contact networks on campus

Studies on RI policies tend to assume that contact in universities is largely informed by En—transcripts showing which courses a student is registered for, or “enrolled” into. En can provide structural insights on density of connections and disease transmission paths to inform modeling disease simulations (61). However, such static data can overestimate attendance and ignore overlap between courses (via instructors) and organic interactions outside classes (e.g., waiting areas, dining, parties, and extra-curricular activities). Therefore, using En can overemphasize the disease-mitigating structural changes to the network by RI interventions. By contrast, WiMob is more grounded in community behavior as it captures multiple scheduled and serendipitous contact situations dynamically over the semester. We compared the features of contact networks constructed with WiMob, against networks constructed with En using data from GT for Fall semester of 2019 (August 19–December 14), prior to any COVID-19 reported cases in the U.S. En approximates contact based on students enrolling for classes that could potentially collocate them in the same room during lectures. WiMob infers contact when any two individuals actually collocate near the same WiFi access point (41,42) for extended period (see explanation in Supplementary Material, WiFi Mobility). We found that WiMob rendered new insight into contact on campus that was invisible to the En methodology.

#### 3.1.1. WiMob characterizes temporal variation in proximity

**Figure 2:**
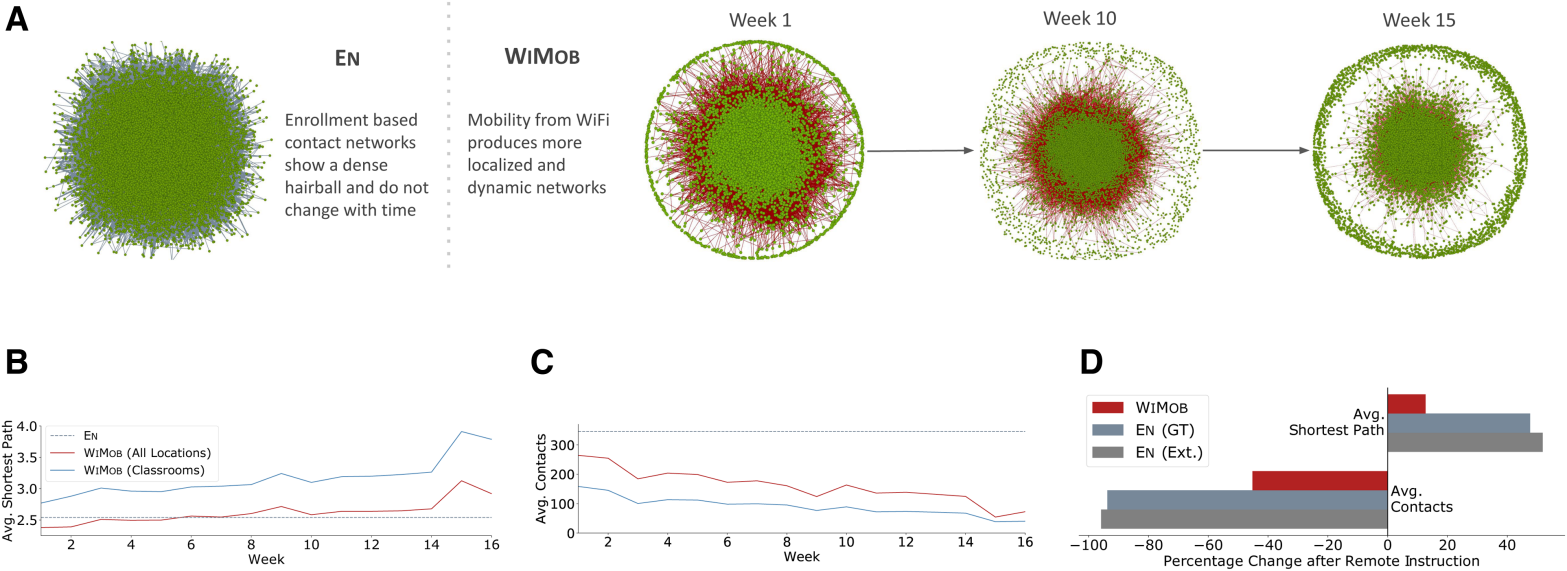
Results show difference in structural characteristics of contact networks from En (course enrollment) and WiMob (campus mobility). (**A**) In general, En overestimates connections (grey edges) between students (green nodes) and does not anticipate changes through the semester. En assumes 90% of students to be connected in a single component, but WiMob reveals (red edges) that on any given week only 69% are in the largest component (those not on campus are isolated and shown in the circumference). Moreover, WiMob reveals that density of connections changes over the semester. (**B**) En depicts campus contacts to be connected closely into a “small world.” WiMob shows that contacts evolve over time. As mobility captures interactions outside classrooms we observe that for the first 6 weeks the shortest transmission path between people is shorter than what is reported by En. (**C**) Enrolling into a course does not necessitate physically collocating with the class for extended periods (students can also choose to be entirely absent). WiMob reflects this behavior and highlights a decline in average contacts over time. (**D**) These structural differences can help policymakers anticipate the effect of closure policies by describing how it fragments the underlying contact network. En shows that remote instruction leads to a 94% reduction in contacts and 50% increase in transmission path length (similar to numbers reported in prior work (11), shown as En (Ext.)). However, the estimate is significantly lower when measured using WiMob. As a result, WiMob emphasizes the limits of remote instruction policies and in turn motivates new policies that can be designed and evaluated with actual on-campus behavior.

Variation in contact over the semester would naturally impact the severity of disease spread. However, En describes a static network that does not capture such dynamics (Figure 2A). Instead, we found that WiMob shows contacts got sparser over the semester. Figure 2C presents a notable decline in contacts after the first two weeks, which coincides with multiple orientation seminars and the so-called “course shopping” period of Fall 2019. In fact, contact decreased considerably in classrooms, with a steeper slope possibly because of reduction in attendance. WiMob was able to reveal other observable changes, such as drop in contacts during exam period (week 15) and increase after fall recess (week 10). En rendered a highly connected static network, which can miscalculate the speed at which a disease spreads. By contrast, the longitudinal behavior represented by WiMob can help universities anticipate disease spread more accurately.

#### 3.1.2. En overestimates contact-based risk

Campuses can assess risk of an outbreak by characterizing the number of individuals that would be at risk of infection through contact. In our study, En indicated 99% of the individuals on campus were clustered in a single component—if any of them would have been infected in Fall 2019, the entire component would be at risk. From the lens of En a virus can exhaust an entire population with infection very early. However, WiMob showed that only 69% of the population was connected in a single component (Table S2). This difference is because WiMob can distinguish how many individuals are active on campus. Therefore, WiMob provides a pragmatic estimate of risk by grounding it in local occupancy and helps campuses budget for resources better.

#### 3.1.3. WiMob reveals different paths for disease transmission

Reports suggest that a key contributor to cases in the pandemic is actually clustering of individuals in non-academic spaces (7). However, En does not depict a holistic view of campus contact. It is limited to classrooms and, therefore, fixates on contacts in lectures, while ignoring other spaces. In fact, WiMob showed that in the first 6 weeks of Fall 2019, the shortest path among individuals was smaller than that approximated by En (Figure 2B). With WiMob, we observed new paths in the contact network from situations outside classes. On a given week, WiMob showed the average shortest path with contact is 3.26(±0.5) when only considering lectures, whereas capturing all contexts reduced the average shortest path to 2.67(±0.28). Characterizing shorter pathways is crucial for policymakers as closure policies by design aim to disconnect these pathways.

#### 3.1.4. En overemphasizes the impact of remote instruction

Prior work uses En to posit that RI reduces contact and in turn significantly fragments the network for disease spread in universities (10,11). We evaluated the effectiveness of such a policy if it were applied in Fall 2019, with both WiMob and En. Figure 2D shows that RI with En reduced contact by 94% and increases shortest path by 50%. However, the same intervention with WiMob showed a relatively milder impact (contact reduction 45%; shortest path increase 11%). This reinforces that contact outside courses are significant and remain unaffected by enrollment-oriented policies like RI. WiMob provides a more encompassing view of the structural effects to a network and motivates design of more impactful closure policies.

### 3.2. Epidemiological model built with WiMob shows that LC yields better infection reduction with lower burden

As outlined above, En does not comprehensively capture the contact on campus. By contrast, contact networks built with WiMob demonstrate new structural insights, which are critical to describe disease spread. A campus is composed of many different spaces, and En does not have the flexibility to design closure of such spaces or assess its impact. These drawbacks naturally motivate a new approach to design interventions. Since WiMob mitigates the limitations of En, we leveraged it to demonstrate the effectiveness of localized closure (LC) policies.

We used WiMob to define the contact structure of each day and simulate COVID-19 with an agent-based model. Our ABM was overlayed by a modified SEIR compartmental model for COVID-19 for each agent. GT also had implemented a robust surveillance program on campus. Hence we calibrated the ABM on the positivity rate for COVID-19 for GT (24) in the first 5 weeks of Fall 2020 also incorporating external seeding from the surrounding Fulton County, GA (25). We validated our model by predicting future trends for the rest of Fall 2020. For robustness, we performed additional calibrations by varying time windows and university context (details in Supplementary Material, Sensitivity Analyses). We studied interventions by applying the ABM over the contact networks produced by WiMob with data from Fall 2019—a counterfactual to Fall 2020 if no closure had occurred (see Supplementary Material, Simulation Model for further details). The results in the main article refer to LC policies derived using *PageRank* (48). The corresponding results for other centrality algorithms are available in the Supplementary Information.

#### 3.2.1. WiMob can model RI and LC interventions with various configurations

**Figure 3:**
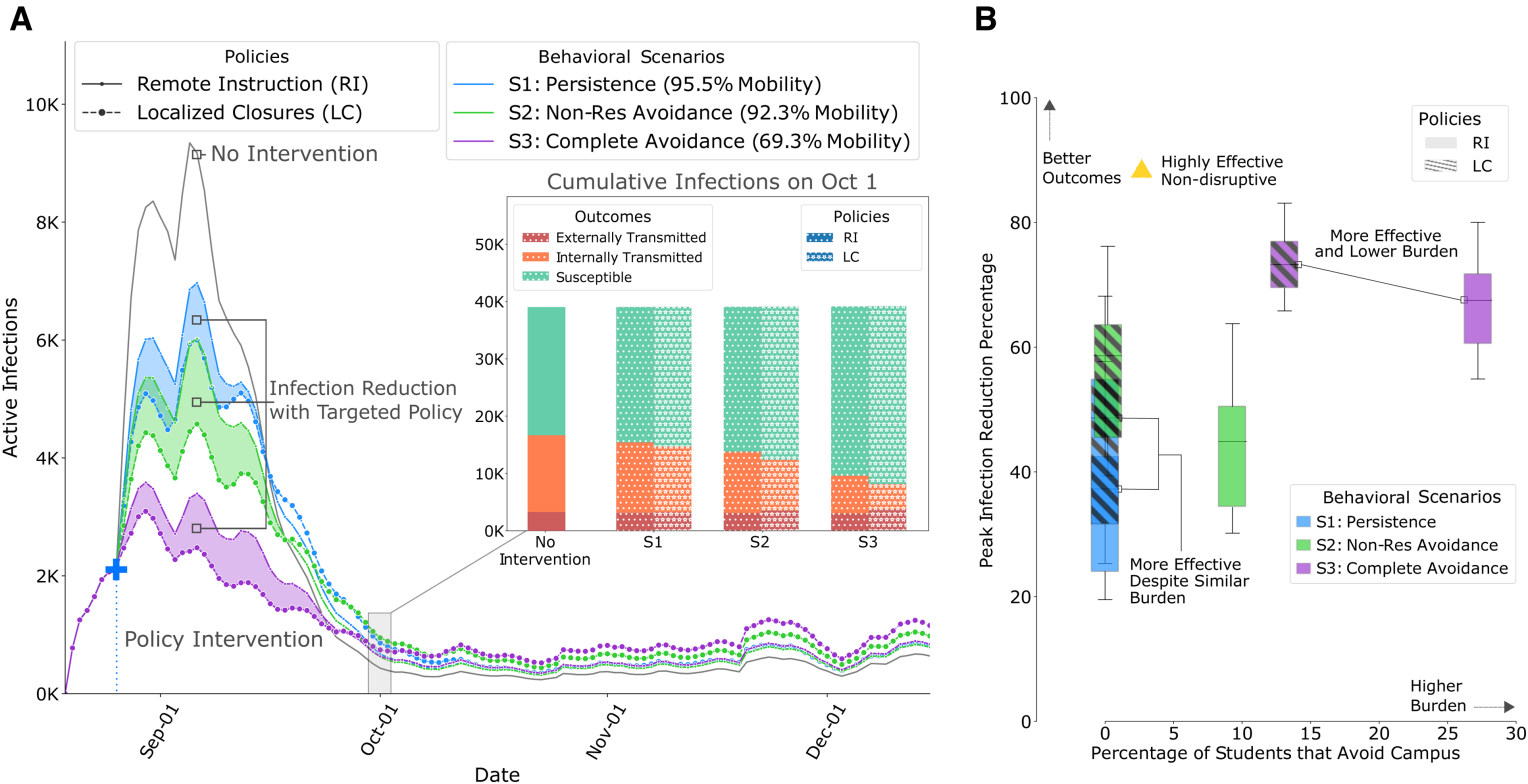
Results of policy interventions with our calibrated ABM on contact networks from Fall 2019, derived from WiMob (**A**) This graph compares the mean active infections between LC and RI. LC show improved outcomes (shaded regions) even when constrained to the same restrictions of RI policies. (**a**)–*inset:* After the first wave, even though LC shows slightly higher active infections, the cumulative infections are still lower, especially those that are a result of internal transmission on campus. Figures S10–S17 show changes in cumulative infections under different policies, including 2.5th and 97.5th percentile intervals. (**B**) Outcomes of policies within the same behavioral scenario are shown with boxes of the same color (RI policies are solid, LC policies are hatched) and box heights represent the 2.5th and 97.5th percentile. In *S1*, even though LC and RI are equally burdensome in terms of students avoiding campus, LC shows improved outcome on peak reductions. In fact, for the other scenarios, LC shows better outcomes than RI, without forcing as many students into online schedules, and, therefore, being even less burdensome with greater impact. Figure S6–S9 show comparison of all policy outcomes with different budgets.

Prior works show a few locations are responsible for majority spread (30) and restricting movement between them leads to greater control (62). We found that, if COVID-19 spread through Fall 2019 (a regular semester), the cases rose after 7 days (Figure 3A). Therefore, we applied both RI and LC interventions after the first week. To devise interventions, WiMob estimated how RI uses the budget and then designed LC to match this budget under every behavioral scenario. Table 2 describes how the budget for each policy varies. Additional details are present in Supplementary Material, Modeling Policy and Scenarios.

**Table 2:** Comparison of policies in terms of controlling the disease and impacts on campus in Fall 2019.

Behavioral scenario	S1: Persistence			S2: Non-res avoidance			S3: Complete avoidance		
Policy	RI	LC		RI	LC		RI	LC	
Budget	-	Mobility (95.5%)	Exposure risk (18,800)	-	Mobility (92.3%)	Exposure risk (16,900)	-	Mobility (69.2%)	Exposure risk (12,700)
Infection reduction outcomes									
Peak infections (%)	25.34 (±12)	36.92 (±14)${}^{**}$	34.30 (±13)${}^{**}$	35.44 (±10)	49.33 (±11)${}^{**}$	52.19 (±10)${}^{**}$	61.62 (±7)	69.34 (±5)${}^{**}$	64.44 (±6)${}^{**}$
Total infections (%)	6.99 (±5)	10.63 (±6)${}^{**}$	8.19 (±5)${}^{**}$	14.88 (±4)	13.96 (±6)${}^{*}$	15.67 (±6)	33.00 (±5)	33.4 (±5)	26.94 (±5)${}^{**}$
Internal transmissions (%)	17.13 (±9)	22.62 (±11)${}^{**}$	21.01 (±11)${}^{**}$	27.58 (±8)	35.35 (±12)${}^{**}$	39.20 (±11)${}^{**}$	54.00 (±8)	70.89 (±7)${}^{**}$	60.90 (±9)${}^{**}$
Burdens on campus									
Locations affected	58	18	19	58	38	50	58	192	124
Students avoiding (%)	0	0	0	9.30	0.20	0.45	27.21	12.45	6.57
Completely isolated on campus (%)	5.42	8.40	8.40	5.95	5.72	5.71	7.09	5.18	5.23

Within each behavioral scenario, we performed the Kruskal-Wallis H-Test (63) to compare outcomes of LC with RI. We found that LC leads to significantly improved peak infection reduction and internal transmission. In terms of reduction in total infections, the outcomes were comparable in general but varied by specific scenarios. In addition, every policy also exerted some burden on campus, either in terms of locations affected, students avoiding campus or isolation. We observed that LC policies focus on fewer locations (except in *S3*). Moreover, these policies affected fewer student’s schedules and therefore fewer people avoid campus due to completely remote schedules. Finally, LC does not increase the percentage of people completely isolated on campus (p-value: ${}^{*}<$0.01, ${}^{**}<$0.001).

We present differences between LC and RI based on three infection reduction outcomes; peak infections (maximum active cases on a given day), internal transmission (exposure from infected individuals on campus), and total infections (cumulative cases at the end of the semester). Additionally, we measured the burden of policy interventions with the number of locations closed—requires resources to monitor and maintain super-spreader locations, the percentage of students that avoid campus—disruption to learning outcomes (13,20), and the percentage of individuals completely isolated—worsens mental wellbeing (64).

#### 3.2.2. LC cause greater reduction in peak infections, while affecting fewer locations

Controlling peak infections relaxes the burden on a university to support positive cases for any given day, and allows resources to be distributed over time. In all behavioral scenarios of our simulation of Fall 2019, we observed that the peak reduction was significantly better in LC (Figure 3) than RI. While RI impacted 58 different locations (classrooms and lecture halls), in *S1* and *S2*, LC achieved better outcomes by closing fewer locations. For example, in *S2*, RI achieved a 28.9% peak reduction, but LC showed reductions of 49.3% (mobility budget) and 48.1% (exposure risk budget). This was attained by closing 38 or 50 locations respectively. Therefore, with such policies, policymakers need to restrict fewer locations to remarkably minimize the pressure of active infections on campus (e.g., diagnoses, treatment, quarantining).

#### 3.2.3. LC lead to comparable reduction in total infections, while keeping more students on campus

Universities want to minimize the number of infected cases while ensuring majority of the population remains active on campus to continue successful operation. In Scenario *S1*, the total number of infections reduced by both LC was more than the reduction shown by RI. were similar. For other behavioral scenarios the total infection reduction between policies was similar (Table S2). In contrast, the impact the policies had on the student schedules was remarkably different. RI forced multiple students to adapt to fully online schedules. In Scenario *S2*, 9% of students did not visit campus and in *S3*, 27% of students did not visit campus. On the other hand, in LC, the number of students expected to avoid campus could be as low as 0 and never exceeded 12%. Besides sustaining economic loss to the campus, remote instruction can increase anxiety among students and hinder learning outcomes (20,21). Compared to RI, LC offers policymakers a way to defend against turnover in the student population, without compromising overall control of disease spread (Table 2). Limiting the number of students that avoid campus helps preserve on-campus businesses (18,19) and minimally disrupts the student wellbeing.

#### 3.2.4. LC cause greater reduction in internal transmission without causing further isolation on campus

Universities are responsible for limiting spread on campus, but they must also ensure that aggressive policies do not worsen mental wellbeing of the community. In terms of internal transmission the reduction is significantly larger with LC (Table 2). However, when LC restricted the infections early in Fall 2019, it left more individuals susceptible to external transmission. College student behavior outside campus on weekends and breaks is known to impact local transmission (65). When policymakers consider LC they should also consider policies on re-entry or required testing based on off-campus activities. In terms of isolating individuals on campus, it’s notable that LC and RI were similar in *S2*. Interestingly, in *S3*, where LC closed more than 100 locations, the percentage of isolated individuals per week was less than that of RI. This finding implies that LC can keep individuals on campus without forcing them into complete isolation. Here “isolation” refers to no form of proximate contact with any individual on campus—extreme social distancing where individuals are not even collocated in the same suite or hall. While social distancing is a recommended countermeasure for COVID-19 (8), complete isolation can have adverse effects on psychological wellbeing (64,66,67). LC can help alleviate concerns of closure interventions that increase loneliness and limit social connectedness (66).

#### 3.2.5. LC identifies a wider variety of auxiliary spaces

By using WiMob to design LC we were able to identify locations for closure at the granularity level of rooms, including unbound spaces such as lobbies and work areas. As policy design budgets changed with every behavioral scenario we found that LC identified different types of locations for closure. First, in *S1*, we found that most locations that LC targeted are a subset of the auditoriums–like rooms where large classes would take place in Fall 2019. Note, LC needs to restrict only a few such spaces to utilize the same budget as RI. This is because, under *S1*, RI policies only altered visits to lectures, while these spaces are used for other purposes during other times (e.g., club activities and seminars). We also noted that LC targeted “high traffic” locations like conference center lobbies which are typically used as waiting areas or for networking events. Next, in Scenario *S2*, we saw that in addition to spaces mentioned earlier, interestingly LC further restricted the use of smaller rooms (occupancy 13–35) which would not be affected by RI (as only classes of size ≥30 are offered online). LC also targeted areas in the recreation center (which includes locker rooms and indoor courts for 4–20 people). This insight indicates that our methodology WiMob accounts for a diverse set of student activities. Moreover, we also found a selection of spaces that would not be frequented by the undergraduate population, such as lab areas and facility buildings like the police station. Lastly, in Scenario *S3*, LC targeted closure of activity in far more spaces than RI. However, the better outcomes can be attributed to the fact that LC diversified the potential restriction areas. LC restricted heavily used small study rooms or breakout rooms (for 1–6 people). Furthermore, it restricts use of spaces where multiple small groups of people can organically assemble, such as cafes, dining halls, and reading areas. We also observed that LC restricted activity in about 10 Greek Houses but does not target other housing areas—demonstrating its ability to restrict social behavior that could amplify disease spread. Figure S18D shows the diversity in locations for various LC policies.

#### 3.2.6. Sensitivity and robustness analyses

The results above use an ABM calibrated on the positivity rate of the first 5 weeks of Fall 2020. This rate can be influenced by many latent factors (e.g., mask-wearing, hand washing, distancing, and compliance). To study any effect of these variations, we also calibrated on different time windows throughout the semester. We calibrate on weeks 5–9 and 10–14 in Fall 2020, and validate on the remaining semester. In both cases, compared to RI, we found that LC still exhibits better reduction in peak infections (up to 90%) and internal transmission (up to 77%). In the original calibration, LC maintained the same level of total infections as RI, but with the new periods we found total infections were substantially less than RI (Tables S8 and S9). Another important variable for positivity is the wider context of the campus e.g. urban/rural, the surrounding county, city, etc. To investigate this, we also calibrated our ABM on the positivity rate of different universities in the US in Fall 2020 (along with information from their county to seed external cases). Consider this as a hypothetical where the mobility of the GT community remains the same but disease outcomes resemble a different campus. We calibrated on data from University of Illinois at Urbana-Champaign and University of California, Berkeley. We found no remarkable differences from our findings with GT (Tables S10 and S11).

## 4. Discussion

Non-pharmaceutical interventions (NPI) are the first line of defense for universities to respond to contagious diseases like COVID-19 (68,69) and are also crucial to control infections and continue operations until recovery. On a campus, a common form of NPI is closure (70). Universities consider enrollment data (En) to design remote instruction (RI) for closure to support continued operations safely (11). However, En can misconstrue contact on campus, and RI policies can have broad impacts despite their effects on curbing the disease spread. This paper demonstrates that repurposing logs from a managed WiFi network (WiMob) can help design effective localized closure policies (LC). We show that WiMob uncovers rich contact dynamics and provides policymakers multiple dimensions to design policies like LC. We simulate COVID-19 with an ABM that harnesses WiMob to compare RI and LC. As universities plan for future semesters, our results present evidence that LC designed with WiMob can lead to improved infection reduction outcomes, while simultaneously relaxing burdens on the campus caused by coarse-grained broad RI policies.

### 4.1. Generalizability for other contexts

In practice LC policies should be deployed in conjunction with the other tools as well like testing, tracing, and quarantining. WiMob can complement disease-specific knowledge to identify closure spaces. For example, small indoor spaces with poor ventilation increase the risk of infection for COVID-19 (71), while other algorithm-identified locations for closure might not require closure because users of a space are compliant with mask-wearing and testing. Further, as a pandemic progresses and public health guidance develops (72), with WiMob, campuses can regulate the restriction of LC policies and anticipate the path to “normal” operations (3,4). Moreover, WiMob captures various spillover effects that cannot be captured in methods like En. For instance, with WiMob we observe that the mobility in Fall 2020 was 39% of that in Fall 2019 because the on-ground policies lead to certain staff working remotely as well. With additional information, WiMob enables policymakers to model such scenarios and design alternatives like LC with new budgets. Policymakers can use WiMob as a versatile tool to explore dynamic intervention strategies as well. Prior work shows that staggering policy restrictions could have variable impact on campus (73). Accordingly, WiMob could be used to build an adaptive version of LC that updates at different points in the semester based on expected mobility changes. Additionally, depending on campus priorities and resource limitations, different campuses can use this same data to model policies differently. The effectiveness of reopening policies is expected to be sensitive to a campus’ specific context that includes physical infrastructure, overarching guidelines, and human compliance (5). For certain campuses policies might not need to be constrained by exposure risk as testing might be frequent, ubiquitous, and voluminous. Other campuses could have limits on quarantining capacity. Policymakers might even consider the cost trade-offs by actually forecasting actual financial losses incurred by reduction in mobility (31), or valuate loss of services based on community needs (74). We elaborate on these considerations in the Supplementary Material, Implications for Policy Design.

### 4.2. Operational considerations

Beyond assessing cost-benefits, universities need to devise practical methods of obtaining, storing, and processing mobility of the community as WiMob. University can access logs from the managed network internally as it is passively collected. Moreover, it does not require any new form of surveillance sensing but universities must revise terms of use and stay sensitive to community perspectives. Despite population mobility being valuable for many applications (75), accumulating localization data can be a major privacy concern (76). Instead, operational applications need to conceive approaches that only retain insights on locations to shutdown but not individual data. Similarly, any operational use needs to employ differential privacy to limit what stakeholders can learn from the data (77) (e.g., decision-makers can only get a list of candidate locations to close). In the Supplementary Material, Discussion, we further detail approaches to reconcile privacy, ethics and legal considerations.

### 4.3. Limitations and future work

For future investigations of better closure policies, researchers and policymakers need to be cognizant of the limitations of our work. Our analyses capture heterogeneity in individual behavior but does not account for differences in intrinsic vulnerabilities, which are related to severity of risk (67,78,79) and disparity in burden of shutdowns on demographic groups (30). WiMob can be extended with other streams of data to characterize sub-contexts in the population and devise new forms of LC to explicitly study the impact of policies on specific vulnerable subgroups in the community. Additionally, our work explores the avoidance based behavioral responses to closure interventions with assumptions in line with prior work (11,52). Researchers and policymakers can be interested in substitution behaviors where the population visits new locations when others are closed. WiMob has the flexibility to model more nuanced spillover effects. Exploring different ways to remove and reallocate edges in the contact network is interesting future work. Lastly, WiMob was built on GT’s managed WiFi infrastructure with expansive coverage reflecting a large proportion of the campus population and its physical space. The coverage at other campuses might vary depending on how the access points are setup as well as the populations connectivity preferences. These variations can lead to under-representation of certain behaviors during policy evaluation. Thus, future studies can investigate the efficacy of WiMob by systematically simulating different configurations of WiFi coverage. Further discussion in Supplementary Material, Limitations and Future Work.

## Data Availability

The code used for processing the WiFi logs into mobility networks can be requested under requisite terms of use agreements. The code for simulation is publicly available at: https://github.com/AdityaLab/cv-wifi-GT. The raw data supporting the conclusions of this article will be made available by the authors, without undue reservation.
