# Supplementary material for: Empirical networks for localized COVID-19 interventions using WiFi infrastructure at university campuses
Source: Front Digit Health. 2023 May 16;5:1060828. doi: 10.3389/fdgth.2023.1060828 (PMC10227502; doi:10.3389/fdgth.2023.1060828)
Supplement: Supplementary file 1 [file Datasheet1.pdf]

Supplementary Information Appendix

1 SUPPLEMENTARY METHODS

In this section, first, we describe the primary data source for mobility models (WiMob), the data used for calibrating our simulations, and for comparison of contact networks with methods using enrollment data (En). Next, we describe how we construct counterfactual mobility networks under the two main policies of interest in our study: remote instruction (RI) and localized closures (LC). Finally, we describe an agent-based-model (ABM) of disease transmission, which has a contact structure based on WiMob, and how this model was calibrated.

1.1 Data

1.1.1 WiFi Mobility

We use data provided by the IT management facility at Georgia Institute of Technology (GT) which accumulates WiFi access point (AP) logs over time. The primary use of WiFi network logs is for maintenance and security purposes. We mine these logs post-hoc to describe the mobility of individuals on campus, which we refer to as WiMob. Here mobility is expressed by visits to certain locations that are demarcated by a corresponding AP. WiMob can also describe dwelling (duration of visits) and collocation (dwelling in the presence of others around the same AP).

The campus WiFi network spans 6959 APs distributed between 240 buildings (and some outdoor locations). We label APs according to which building they are inside, along with the closest room or space (e.g, hallway, lobby, suite, cafe, etc.). The AP may or may not reside inside the room, however, in most cases, only a single AP is associated with space. For less than 5% of the APs, the AP shared association to space with another AP. This many-to-one mapping is typically in the case of large halls and auditoriums. We resolve such many-to-one associations by using APs as a proxy of the space they are associated with. Therefore, individuals connected to different APs in the same space will still be identified as collocated. Similarly, an individual could connect to the network with multiple devices. However, less than 1% logs show that a user is connected to multiple APs around the same time. Therefore, WiMob is agnostic to which device connects to the APs to proxy the presence of the individual. For this study, we obtain the WiFi network logs retrospectively for all of Fall 2019, and the data for Fall 2020 was provided on a per-day basis. Each day, approximately $33,000$ different people connect their devices to the WiFi network on campus. Overall in Fall 2019, approximately $40,000$ different people connected to the campus network.

1.1.2 Asymptomatic surveillance testing data

We calibrated the ABM using the publicly reported positivity rate on the GT campus as reported through the asymptomatic surveillance and diagnostic testing program [28]. The testing program used pooled saliva sample surveillance with follow-up diagnostic testing. The positivity rate was reported each day, but individuals must wait at least 1 week between tests. We aggregated the positivity rate by week during the Fall 2020 semester.

1.1.3 Confirmed case data

When calibrating our ABM, we considered the reported confirmed cases in Fulton County [27], the county in which GT is located. The 'Confirmed COVID-19 Cases' reported in this dataset are cases that have been confirmed with a positive molecular (PCR) test. We considered cases during the Fall 2020 semester to inform external transmissions in the ABM.

1.1.4 Enrollment network summary statistics

We compare structural properties of contact networks constructed with WIMOB to contact networks constructed from GT's course enrollment transcripts (EN) To ensure that individuals cannot be identified by combining anonymous WiFi network logs and course enrollment transcripts, we only use aggregate statistics from EN— structural characteristics of the contact networks described in Table S2. The EN network was based on Fall 2019 transcripts for GT's Atlanta campus. These were cleaned to account for cross-listed courses and was used to determine which students were classmates with each other to form a contact network.

1.2 WiFi Mobility Models

1.2.1 Inferring location from Logs

WIMOB is our approach to describe contact between people and movement of people between locations. The first step requires using WiFi network logs to infer when individuals were at specific locations on campus by determining when devices were connected to the corresponding APs. Our system mines the WiFi network logs that are populated via the Simple Network Management Protocol (SNMP) — a standard and widely used monitoring protocol to organize device association behavior to a WiFi network. Periodic SNMP updates can be caused either by poll requests to the APs that log which devices are associated with it at that time. However, devices can appear invisible to detached from an AP for multiple reasons, for example, when devices are idle. Otherwise. SNMP updates can occur whenever a new device connects, which is typical when individuals move between APs. Our approach exploits this factor to first mine periods when individuals are moving, then identify periods of dwelling between movements, and finally determine collocation when two or more individuals are dwelling near the same AP. This system follows from other studies that mine WiFi logs [12, 36] and the detailed processing pipeline and evaluation is presented in [8]. This system to infer collocations has been tested against lecture attendance and reports a high precision of 0.89, but a relatively lower specificity of 0.79 [8]. While it is not likely to show false-positives, it has a possibility to erroneously mark people absent from a location even though they were there. However, for the purposes of our study, a contact network is made over an entire day and it only needs a single collocation instance for us to consider contact. And therefore we believe this limitation would not significantly affect our models.

1.2.2 Characterizing Logs as Contact and Movement Networks

After inferring where an individual is located on campus, we represent the entire community behavior as graphs. We describe a bipartite graph, K, that shows when a user is at a given location on campus (Figure Figure S1). This bipartite graph has edges connecting a set of m people, P, to a set of n locations, L. An individual can have multiple edges connecting to the location if they visited that location multiple times (e.g., t_1, t_2). The edge data contains the start and end times of these dwelling periods. For these bipartite graphs, we make a projection on set P to describe collocation. This projection graph, G, contains an edge between users if they were visiting the same location during overlapping times. Since we do

not use RTLS, our approach can only identify if people were in the vicinity of the same AP, but does not describe the distance between them. However, it can reasonably determine collocation in the same room [8]. Since our study is limited to localizing people indoors, we adapt the definition of *proximate contact* [17] where people might be "more than 6 feet but in the same room for an extended period". In our work, we use a lower bound threshold of 40 minutes to determine proximate contact. Therefore, individuals are only considered in contact when they are collocated in a room for 40 minutes or more. This threshold was set up to account for typical lecture duration on campus (for standard 3-credit hour courses taught 3 times a week). Additionally, we compared the clustering coefficient of the contact networks for different days by varying contact thresholds as 30 and 40 minutes. The Pearson's r correlation of these was very high 0.97. Thus, we chose to use the 40 minute threshold as it produced structurally similar graphs while requiring lower space constraints. Every edge between two individuals contains a list of locations where they were possibly in contact. G forms the basis of the contact-network that we use an agent-based model to simulate. Alternatively, we also make a projection on the set L. This projection is a directed graph, H, where an edge from L_i to L_j represents movement from the first location to the next within a span of 60 minutes. GT's large urban campus with pedestrian pathways and motorized transit services enables direct movement between any two places on campus within the threshold. The 60 minutes threshold helps discount erroneously labeling returning from outside campus (e.g., non-residential students visiting two different locations between 2 days). H effectively describes how locations are connected and which locations could be more conducive to attracting and disseminating the virus. As a consequence, the H helps inform policy design. We compute the bipartite graph and its projections for each day of the semester.

1.3 Modeling Policies and Scenarios

1.3.1 RI: Offering Large Classes Online

As a response to COVID-19, prior work has recommended using EN to enforce a form of RI— moving classes large to an online remote instruction setup while other classes are offered in–person [16, 5, 38]. While we have access to aggregate insights on EN contact networks, our study protocol prohibits us from accessing course-specific information at an individual level. Therefore to infer individual enrollment, we analyze the edges of the bipartite graph K. For this, we first scrape the GT's course roster for Fall 2019 (filtered to only represent the Atlanta campus). This process provides us with a location and weekly schedule for every lecture conducted on campus, including its various sections. With this information, we are able to identify which edges represent visits to lectures, and subsequently, we can account for unique visitors to a lecture. Thus, we can first identify the number of unique individuals on campus who are enrolled in classes. The aggregate data from course enrollment reports that $21,299$ students were enrolled in Fall 2019. In comparison, our inference identifies $22,248$ students. The excess number can be explained by the fact that our method does not distinguish between instructors, TAs, and students. Next, we study the unique visitors to every lecture in the scraped course schedule which gives us an estimate for the size of every class. Given the limitations of our data processing, actual enrollment sizes could be larger, but our process is less likely to count false positives [8]. Finally, to model RI, for the contact network G_t, we create a counterfactual network G'_t for each day t. These exclude collocations that took place at lecture locations during lecture times. If two people were connected solely by proximity during lectures — in a class with large enrollment — they will appear disconnected in the counterfactual network.

1.3.2 LC: Closing Important Locations

This article demonstrates the effectiveness of localized closures,LC, which are targeted interventions to seize mobility at different spaces on campus. For this, we identify important locations on campus by analyzing H. In the main paper, LC uses *PageRank* [29] as an illustrative algorithm to identify important location nodes. For robustness, we apply various additional algorithms to identify highly authoritative nodes in H — betweenness centrality [13], eigenvector centrality [4], and load centrality [25]. In the SI Appendix, we distinguish these different policies as $\mathsf{LC}_{\text{PRank}}$, $\mathsf{LC}_{\text{BCen}}$, $\mathsf{LC}_{\text{ECen}}$, $\mathsf{LC}_{\text{LCen}}$. Since RI captures a weekly schedule to determine enrollment, LC is implemented to find locations based on behavior from the past 7 days of mobility. We apply the weighted version of the algorithms mentioned earlier on the directed graph representing movement, H. The edge weight is based on the number of instances of movement between any L_i and L_j. After sorting the locations by importance, we determine the number of locations to shut down based on different budgets induced by RI— mobility and risk of exposure. For this purpose, we take the approach of a greedy algorithm which successively removes highly-ranked locations till the constraint is met (within 1% margin of error). Similar to RI, LC also render counterfactual collocation networks, $G"_t$ for each day t. In these networks, we remove instances of collocations that occurred at the shutdown locations. Figure S18d and Figure S19 shows the categories of buildings where different spaces are closed by LC policies.

1.3.3 Inducing Budgets and Characterizing Behavioral Scenarios

We now describe how we compare the RI and LC policies. First, we consider the effects of these policies under three behavioral scenarios. These scenarios express the spillover effects of closure that lead to students avoiding campus entirely because their entire schedule is forced online. This analysis assumes that the motivation to be present on campus is determined primarily by enrollment. We consider that, if a student has a full course load (enrolled in a minimum of 3 classes) and all their classes are offered online, that student might have less incentive to visit campus at all (for any engagement) and thus practice *Avoidance*. Since LC could end up closing classrooms, it can also lead to academic schedules being affected and elicit *Avoidance* behavior. As a result, we describe three behavioral scenarios. *Persistence*, is the preliminary, or null scenario, which represents no *Avoidance*. This counterfactual collocation graph only removes edges directly affected by RI or LC. The second scenario we model is *Non-Residential Avoidance* where only non-residential students with full online schedules stop visiting campus entirely. Here the counterfactual graph will remove all edges of non-residential students with fully online schedules. Lastly, the third scenario we model is *Complete Avoidance* where any student with fully online schedules stops activity on campus entirely (including residential students). Here the counterfactual graph will remove all edges from any student with fully online schedules. Since our study protocol prohibits us from mapping our data to other sources, we heuristically infer which individuals are likely to be residential and which are not. We label individuals as residential when they dwell an average of at least 15 minutes at residential locations between 6pm and 10am, on workdays (Monday–Thursday).

Under each behavioral scenario, we limit the number of locations that can be closed under the LC policy to ensure the level of restriction is constrained to be similar to the RI policy. We limit the number of locations under two types of restrictive budgets. The first budget is based on *mobility*, which is the percentage of edges remaining in the bipartite graph if a policy were to be implemented. The second budget is based on *exposure risk*, which is the number of unique individuals who would be in the 1-hop collocation neighborhood of positive individuals. We compute this budget by randomly sampling 2.5% of the population as positive, based on the highest 7-day average positivity rate reported by GT [15] in Fall 2019. Note, however, the effect of RI on campus can vary in different behavioral scenarios, thereby

changing the budget available to design a comparable LC policy. For instance, the number of people at exposure risk is much lower in *Complete Avoidance*. As a result, we build multiple alternate networks representing the effect of policies under counterfactual behavioral scenarios.

The infection reduction outcomes and burdens of different policy interventions (under various behavioral scenarios and budgets) is described in Table S4—Table S7 presents boxplots that compares the distribution of disease control outcomes. Figure S10—Figure S13 show cumulative plots of disease control outcomes

1.4 Agent-based Model

We constructed an agent-based model (ABM) that captures the spread of COVID-19 between individuals active within the GT community. The model is used to evaluate the effectiveness of different policy interventions. We consider a modified version of the SEIR framework for simulating the spread of COVID-19 [34, 7] by using an underlying contact network given by WiMob. Figure S2 shows the compartments of the framework. The *susceptible* state (S) represents individuals who have not been infected and can contract the disease by having contact with an infectious individual. The *exposed* state (E) is canonically equivalent to the "incubation period" and is similar to the pre-symptomatic state found in related work [39, 18]. Individuals are considered *infectious* when they are in either the *asymptomatic* state (Asym) or *symptomatic* state (Sym). Individuals in the *asymptomatic* state are assumed to be the major "spreaders" [18] and transmit the infections to *susceptible* individuals before they are *recovered* (R) [23] — after 7 days [18]. Since *asymptomatic* is considered a state of mild severity [32], individuals in this state do not have a risk of fatality. By contrast, for individuals in the *symptomatic* state, will be eventually *isolated* (Iso) (e.g. self-quarantine, or hospitalization on campus). Once in the *isolated* state, they cannot transmit the disease to individuals in the *susceptible* state. Unlike the *asymptomatic* track, the *symptomatic* state is considered critical severity. Therefore, after moving to the *isolated* state, individuals have risk of fatality and entering the death state (D). If the *isolated* individual survives, they enter the *recovered* state. We assume immunity is preserved and therefore after recovery the individual is no longer *susceptible*.

1.4.1 Definitions

Let $t = \{0, 1, 2, 3, ..., T\}$ be the index of days in simulations. We denote the sequence of dynamic collocation networks indexed by day t, as $\{G_t(A_t, B_t)\}_{t=0}^{T}$. A_t is the set of vertices, i.e. individuals on campus, and B_t is the set of edges. The universe set of the population throughout the simulation time period is given by $M = \bigcup_{i=1}^{T} A_t$. For convenience, we use $a_i \in M$ to index every person in the universe population set.

The SEIR model consists of seven compartments. Each of these corresponds to a function of population subsets with respect to day t: *susceptible* S(t), *exposed* E(t), *asymptomatic Asym*(t), *symptomatic Sym*(t), *isolation* I(t), *recovered* R(t), and *dead* D(t). For example, $a_i \in I(t)$ means a_i is in the *isolation* state at day t. We use $N^t_{S\to E}$, $N^t_{E\to Asym}$, $N^t_{E\to Sym}$, $N^t_{Asym\to R}$, $N^t_{Sym\to I}$, $N^t_{I\to R}$, and $N^t_{I\to D}$ to denote the transitions between states between day t and day $t+1$.

1.4.2 Model Initialization

The entire population M is fixed where $M = S(t) + E(t) + Asym(t) + Sym(t) + I(t) + R(t) + D(t)$ for all t. To capture the positivity out of the students coming back to campus at the start of the semester, we initialize the system by setting a subset of M into $Asym(0)$ and the reminder into $S(0)$. The initial

percentage of *asymptomatic* is described by:

$$Asym(0) \sim Binomial(M, I_0)$$

$$S(0) \sim M - Asym(0)$$

where I_0 is a parameter defined as the initial percentage of *Asymptomatic* at day $t = 0$.

1.4.3 New exposures

We consider two ways that an individual in the ABM could be exposed: (i) exposures that occur due to contacts among individuals captured by the mobility network (*internal transmission*) and (ii) exposures that occur due to contacts that occur outside of the mobility network (*external transmission*).

Internal transmissions happen exclusively among individuals in the model. On any given day, an edge becomes effective, when one of the *susceptible* individual comes in contact with the other which is infectious, i.e. *asymptomatic* or *symptomatic*, individual. Therefore, for every effective edge between two such people, the probability of the susceptible individual getting *exposed* is described by the transmission probability p, which is another model parameter. The probability for an susceptible individual a_i entering *exposed* at the end of day t is given by the following function:

$$f_p(a_i, t, p) = \begin{cases} 1 - (1-p)^{e(t,a_i)}, & \text{if } a_i \in V_t \\ 0, & \text{otherwise} \end{cases}$$

Here, $e(t, a_i)$ is the number of effective edges of individual a_i at time t. Since $(1-p)^{e(t,a_i)}$ is the probability that a_i does not contracted the disease at time t under $e(t, a_i)$ Bernoulli trials, $1 - (1-p)^{e(t,a_i)}$ is the probability that at least one effective edge leading a_i to *exposed*.

In addition to exposure due to internal transmission, we also consider new exposure due to external transmission. We consider external transmission to be exposure resulting from the physical collocations outside the scope of mobility network. For instance, the WiMob does not capture the connections between individuals without access to the campus WiFi or someone contacting infectious persons outside the campus. To reflect this risk in our model, for any day t, $I_{out}(t)$ describes the probability of infection on day t from a collocation that is external to the mobility network. We assume that the probability an individual is infected due to an external source is proportional to the number of cases in the broader community. Therefore, we model the probability of external infection as a function of confirmed cases in Fulton county, where GT is located [27]. C_t represents the confirmed cases reported by Fulton County where C_{max} is the maximum number of the cases over the whole period, $I_{out}(t)$ is given by

$$I_{out}(t) = \alpha * \frac{C_t}{C_{max}} \tag{S1}$$

where α is a parameter scaling the normalized confirm cases in the surrounding county. The resulting number of external infections on day t is then modeled to be are Binomial with $|S(t)|$ trials with probability of success $I_{out}(t)$.

In summary, for every day $t > 0$, the overall number of individuals that become newly exposed is represented as $N^t_{S \to E}$ which is the result of both external and internal transmissions.

$$N^t_{S \to E} \sim \underbrace{\textit{Binomial}(|S(t)|, I_{out}(t))}_{\text{external infections}} + \underbrace{\sum_{a_i \in M} f_p(a_i, t, p)}_{\text{internal transmissions}}$$

1.4.4 Model dynamics after exposure

After exposure, individuals in the model will progress through other disease states in our model. We update the number of individuals in each state daily to reflect transitions between them. The transitions between the states on day t are summarized according to the following equations:

$$\begin{aligned}
S(t+1) - S(t) &= -N^t_{S \to E} \\
E(t+1) - E(t) &= N^t_{S \to E} - N^t_{E \to \text{Asym}} - N^t_{E \to \text{Sym}} \\
\textit{Asym}(t+1) - \textit{Asym}(t) &= N^t_{E \to \text{Asym}} - N^t_{\text{Asym} \to R} \\
\textit{Sym}(t+1) - \textit{Sym}(t) &= N^t_{E \to \text{Sym}} - N^t_{\text{Sym} \to I} \\
I(t+1) - I(t) &= N^t_{\text{Sym} \to I} - N_{I \to D} - N_{I \to R} \\
R(t+1) - R(t) &= N_{I \to R} \\
D(t+1) - D(t) &= N_{I \to D}
\end{aligned}$$

After an individual has been exposed, they will spend Δ_{S} days in an incubation period. At day Δ_{S} after their exposure, individuals will become a *symptomatic* infection with probability p_S. Otherwise the agent will become an *asymptomatic* infection This process is given by the following two equations:

$$N^t_{E \to \text{Sym}} \sim \begin{cases} \textit{Binomial}(|E(t - \Delta_{\text{S}})|, p_S), & t \geq \Delta_{\text{S}} \\ 0, & \text{otherwise} \end{cases}$$

$$N^t_{E \to \text{Asym}} \sim \begin{cases} |E(t - \Delta_{\text{S}})| - N^t_{E \to \text{Sym}}, & t \geq \Delta_{\text{S}} \\ 0, & \text{otherwise} \end{cases}$$

Individuals who enter the *asymptomatic* state will recover after $\Delta_{\text{Asym} \to R}$ days since they were first *exposed*. Thus, we represent the number of transitions from *asymptomatic* to *recovered* on day t as:

$$N^t_{\text{Asym} \to R} \sim \begin{cases} N^{t - \Delta_{\text{Asym} \to R}}_{E \to \text{Asym}}, & t \geq \Delta_{\text{Asym} \to R} \\ 0, & \text{otherwise} \end{cases}$$

On the other hand, individuals who enter the *symptomatic* will eventually enter the *isolation* state [18]. The time that individuals spend in the *symptomatic* state before entering the *isolated* state is normally distributed $\delta^t_I \sim \textit{Normal}(\Delta_I, \sigma^2_I)$. We simulate each individual's transition between *symptomatic* and

isolated by using a sampling function $\Gamma(a_i, t, \Delta_t)$ and a function $\tau(a_i, t)$ that returns the days since *exposed* respectively:

$$\Gamma(a_i, t, \delta_I^t) = \begin{cases} 1, & t - \tau(a_i, t) \geq \delta_I^t \\ 0, & \text{otherwise} \end{cases}$$

$$\tau(a_i, t) = \begin{cases} \text{first day of } a_i \text{ entering } \textit{exposed}, & a_i \in \textit{Sym}(t) \\ +\infty, & \text{otherwise} \end{cases}$$

The aggregated transitions $N^t_{\text{Sym}\to I}$ between *symptomatic* and *isolated* is the sum of the distribution above on each day t.

$$N^t_{\text{Sym}\to I} \sim \sum_{a_i \in M} \Gamma(a_i, t, \delta_I^t)$$

Individuals who enter the *isolated* state may end up with one of two states: *dead* or *recovered*. We defined $N^t_{I\to D}$ as following another binomial distribution with parameter p_D:

$$N^t_{I\to D} \sim \textit{Binomial}(|I(t)|, p_D)$$

The transitions between *isolation* and *recovered* is quite similar to the transitions between *symptomatic* and *isolation* except $\delta_R^t \sim \textit{Normal}(\Delta_R, \sigma_R^2)$ where Δ_R and σ_R are the two parameters standing for the mean and standard deviation of days for an individual in the $isolation$ state entering $recovered$ since the first day of infection. This leads to:

$$N^t_{I\to R} \sim \sum_{a_i \in M} \Gamma(a_i, t, \delta_R^t).$$

Table S1. Model Parameters of the ABM

Parameter	Definition	Value	Std	Source
p	Transmission probability: For any edge between a *susceptible* and *infectious* individual in the contact network, p is the probability that the *susceptible* person will enter into the *exposed* state. This only dictates internal transmission	0.034	0.007	Calibration
α	Scaling factor of the normalized confirmed cases in the surrounding county (S1). This is the parameter for us to generate $I_{out}(t)$	0.032	0.0032	Calibration
I_0	Proportion of population that is *asymptomatic* at day 0	0.012	0.0009	Calibration
p_S	Probability of *exposed* persons becoming symptomatic	0.66	-	[18]
Δ_S	Incubation period (days) since the first day of exposure	5	-	[18]
$\Delta_{\text{Asym}\to R}$	Asymptomatic duration (days); it is the time taken for an *asymptomatic* person to recover since the first day of exposure	7	-	[18]
Δ_I, σ_I	Time of an *symptomatic* entering *isolated* since the first day of exposure of a *symptomatic* person	8	2	[14]
Δ_R, σ_R	Time for recovery for a *symptomatic*, since the first day of exposure	12	2	[20]
p_D	Death rate under isolation	0.0006	-	[20]

The variables p, α, and I_0 are estimated by calibrating the simulation model on the first 5 weeks of positivity rates provided by GT surveillance for Fall 2020, while incorporating external cases from Fulton County. These parameters were found by validating the ABM on the remaining weeks of Fall 2020. Figure S3 shows model estimate during the calibration and validation period.

1.4.5 Model calibration

Most of our model parameters can be estimated from previous studies (see Table S1). However, three parameters in our study are not easily estimated from previous studies: (i) the proportion of the agents that begin the semester asymptotically infected, I_0, (ii) the probability of transmission between a given infectious individual and susceptible individual given a contact in the mobility network, p, and (iii) the scaling factor α used to determine probability of transmission due to contact outside of WIMOB network on day t, $I_{out}(t)$ (see (S1)). We fit these three parameters to the published weekly positivity rate (percentage of asymptomatic cases) as reported by GT's asymptomatic surveillance testing program [28]. To fit the parameters, we performed calibration to minimize the root mean square of error(r.m.s.e) between the simulation estimates of the weekly positivity rate and the observed weekly positivity rate on GT's campus of the Fall 2020 semester as reported by the surveillance testing program.

To perform the calibration, we used two sets of public data pertaining to 2020 Fall semester at GT: (i) the confirmed cases in Fulton County [27], and (ii) the aggregated surveillance test positivity rate for each week [28]. The former helps estimate the daily external infection percentage. The latter is the ground truth trajectory we fit our model on. We consider the data aggregated by week because each individual on campus can only get tested once per week. The positivity rate provided by the surveillance testing data can be interpreted as the estimated percentage of new *asymptomatic* cases out of the total testable population which includes *susceptible*, *exposed*, and *asymptomatic* — with an assumption that every testable population get tested at the same rate.

To formalize the calibration problem, let R_w be the surveillance-testing aggregated result at week w. Let $S(I_0, \alpha, p, w)$ be the function of the simulation model which returns the percentage of new *asymptomatic* in week w out of the total testable population. For every combination of parameters, the predicted result for each week w is estimated by taking the average of N simulation outputs. The objective function is:

$$f(I_0, \alpha, p) = \sqrt{\frac{1}{W}\sum_{w=1}^{W}\left(\frac{\sum_{i=1}^{N} S(I_0\alpha, p, w)}{N} - R_w\right)^2}$$

The optimization problem is:

$$\min_{I_0, \alpha, p} f(I_0, \alpha, p)$$

We fit our model to the first 5 weeks of Fall 2020 and validate the results on the remaining weeks. After obtaining the optimal set of parameters, for robust comparison of policies with different viral variants, we generate a range of parameters by compromising the r.m.s.e within 40% of the minima [7]. First, we implement the Nelder Mead method [22] to discover the optimal set of parameters that minimizes the r.m.s.e. Next, we sample 40 different combinations of parameters within 40% of the minimum r.m.s.e to estimate the means and standard deviations of these parameters (Table S1). Throughout this paper, we pool together all simulation results across those parameters over multiple runs ($N = 15$) and report the 2.5th and 97.5th percentiles of the simulation outputs for every policy experiment.

1.5 Sensitivity Analyses

In this section, we design complementary experiments to inspect the robustness LC policies under different setups and calibration approaches. These variations are defined as follows:

Calibration periods (V1): For the results in the main paper, we discuss results with our ABM calibrated on the first 5 weeks of surveillance testing data. For additional analyses, the model parameters are re-estimated based on the surveillance data from week $5 - 9$ and $10 - 14$ in Fall 2020 at GT. The calibration is validated on the remaining weeks in the semester. Figure S3 shows the calibration and validation. The results of policy comparison with these variations can be found in Table S8 and Table S9, for weeks $5-9$ and $10-14$ respectively. Additionally, Figure S8 shows boxplots to compare the distributions of different policies, while Figure S14 and Figure S15 show cumulative plots of the disease control outcomes, for weeks $5 - 9$ and $10 - 14$ respectively.

Campuses and counties (V2): For the results in the main paper, the calibration of our ABM reflects certain latent factors inherent to GT that could affect both mobility behavior as well as testing results. To complement this we consider calibrating our data under different settings informed by surveillance testing from other similar large universities. This analysis is intended to represent the GT community in a different geographic setting, which is influenced by a different surrounding community, policies and resources. The new parameters are estimated based on the first 5 weeks of surveillance testing from the University of Illinois at Urbana-Champaign (UIUC) and the University of California, Berkeley (Berkeley) [26, 33], and the corresponding county data [10, 9] The calibration is validated on the remaining weeks in the semester. Figure S4 and Figure **??** show the calibration and validation for UIUC and Berkeley respectively. The results of policy comparison with these variations can be found in Table S10 and Table S11. Additionally, Figure S9 shows boxplots to compare the distributions

of different policies, while Figure S16 and Figure S17 show cumulative plots of the disease control outcomes.

The estimated parameters with these calibration variations are described in Table S3. Both RI and LC are evaluated in the same infection reduction metrics and burden metrics again under behavioral scenarios S1, S2, and S3. Since the budgets are structural (mobility, and exposure risk) the LC policies are unchanged among the variants. Moreover, since the burden metrics are structural, those results are invariant.

2 SUPPLEMENTARY DISCUSSION

2.1 Implications for Policy Design

To evaluate the efficacy of policies, we inspect infection reduction by simulating the disease with contact networks from Fall 2019. Since managed WiFi networks accumulate logs for long periods of time, policymakers can use WiMob to model data from previous semesters and experiment with closure policies like LC. We show that WiMob can provide retrospective disease–mitigating insight into multiple counterfactual behavioral scenarios. For instance, policymakers can consider studying seasonal behaviors over multiple semesters for more robustness. Since the underlying data is longitudinal, it provides the flexibility to realistically assess policy interventions at different time points and also study updating policies. Restricting movement on campus at different time-points is known to exert varying degrees of control on disease spread [7]. Our data also shows that mobility on campus varies across the semester and therefore, allows policymakers to consider loosening shutdowns depending on the phase of the semester.

Policy design is determined by practical budgets. We model two kinds of budgets, mobility reduction and risk of exposure. The former represents disruptions in space utilization, availing services, and social life. The latter translates to the testing burden on campus. Our analysis determines the budget in different behavioral scenarios by observing the changes to the graph when large classes are moved online. This is to ensure an equitable comparison with targeted policies. However, in real situations, these budgets can be relaxed or restricted based on that campus' preparedness to tackle a pandemic. For instance, a hypothetical campus that can test everyone every day might not be constrained by risk of exposure. Alternatively, policymakers can model other tangible budgets such as the capacity in isolation wards or available hospital beds. This can be informed by practical limitations of the campus. Similarly, this paper only assesses limited forms of cost, e.g., students avoiding campus or closing locations. From a financial perspective, university campuses can digitize their core service—education—but still realize losses from other curtailed services [21, 3, 37]. When students avoid campus it can lead to direct losses from meal passes and parking and also quantifiable losses to learning outcomes [1, 11] Policymakers can compute actual costs by complementing this data with information from other sources (e.g., revenue generated by cafes and stores on campus). This can help qualifying WiMob to reflect different costs and in turn help design policies that optimize for financial losses. Different campuses have different priorities and challenges in implementing policies.

2.2 Privacy, Ethics and Legal Considerations

We purposefully compare our prototype targeted policies against moving classes online because of practical budgets within the university. Both the WiMob and En based contact networks are derived from archival data accumulated by universities. This does not require instrumenting campus or its community with any new form of surveillance infrastructure. However, its use for a different purpose demands approval

by an IRB. Moreover, acquiring these kinds of data would require collaborating with data-stewards (e.g., the IT department) to establish a data-use agreement. This document must clarify how the data will be de-identified, transferred, and stored.

For this form of data, the critical privacy challenge might not be localization itself, but rather the aggregation of data over a period of time [35]. Data spanning a longer period are more susceptible to cross-analyzing and identifying. To mitigate over-accumulation of data, we suggest an adherence to principles of data minimization [31]. Instead of storing entire mobility graphs, the campus can compute and preserve only high-level insights, such as the importance of locations. This redacts any underlying individual behavior and corresponding identifiable information. Actually, for future purposes campuses can consider a form of differential privacy that authorizes limited forms of data querying depending on the privileges of the stakeholder [2].

An operational application would require the university to update the terms of use for its managed network. Particularly, the university should disclose how this data can be used in critical circumstances that invoke shared vulnerabilities [6]. On notifying the campus community of this change it offers individuals the choice to refrain from using the university network. Prior work on a sample within the same university campus shows that 90% of students are connected to the network on any given day [8]. Therefore, proposing such an opt-out condition can be viewed as an unfair choice. As a result, the campus needs to develop a contingency plan to accommodate network access to users who do not want their mobility behavior to constitute the aggregated insights.

2.3 Limitations and Future Work

This work presents evidence that university campuses can repurpose existing data sources to inform the design of LC policies that can control COVID-19. We evaluate these policies as alternatives to other data-driven, but, broad impact policies that universities consider implementing, such as moving large classes online. One of the drawbacks of this analysis, however, is that it assumes all edges to be the same. For example, when constraining by mobility, in real scenarios losing certain visits might be more valuable than others. Decline in mobility around profit-making services, such as shops and cafeterias, versus losing mobility at common rooms have a different tangible effects on campus. Currently, we take an agnostic stance towards the mobility behavior, where all visits at all locations are the same. In reality, implementing policies could have inequitable qualitative impacts despite appearing to have a similar network configuration. This can be improved by embedding more qualitative information into the network and conceiving ingenious ways to associate costs to edges.

Similar to the assumption that all visits and locations, the current work also assumes all people to be equal. However, different people have different underlying conditions that can make their vulnerabilities more concerning [30]. The privacy safeguards of this study restricted the research team from acquiring any additional demographic or historical information. Further work can attempt to characterize the nodes by randomly seeding the network to reflect the approximate demographic break up of the community. Alternatively, researchers could try to estimate some demographic based on behavior as well. However, to leverage accurate individual information, even for operational use during a public health emergency, policymakers and researchers need to develop new privacy protocols [24].

Lastly, this paper only studies three rudimentary behavioral scenarios, *persistence*, *non-residential avoidance*, *complete avoidance*. Yet, other substitution behaviors are possible and the richness of networks leveraged with WiMob enables the exploration of various new scenarios that can be triggered by policy interventions on campus. For instance, individuals might not even visit transitory spaces, such as lobbies

or cafes between classes. Certain collocations could be the consequence of social ties which might never be developed because of a shutdown (e.g., project teams meeting outside of class). Further research can illuminate the effects of policies in more specific scenarios by modeling post-intervention behavior more accurately.

Table S2. Comparison of Contact Network Structure (Fall 2019)

	Cornell		Georgia Tech				
Contact Network	EN		EN		WIMOB		
Contact Situations	Course Lectures	RI	Course Lectures	RI	All Spaces	Course Lectures	RI
Number of Active Nodes	22051		21299		15379($\pm$3353)	15379($\pm$3353)	15380($\pm$3353)
Average Contacts	529	$22-41$	341	30	152($\pm$63)	86($\pm$35)	86($\pm$34)
Density	0.024	0.001	0.016	0.001	0.009($\pm$0.002)	0.005($\pm$0.001)	0.005($\pm$0.001)
Largest Connected Component(%)	0.991	0.763	0.994	0.627	0.999($\pm$0.001)	0.999($\pm$0.02)	0.978($\pm$0.025)
Average Shortest Path	2.47	3.75	2.54	3.54	2.67($\pm$0.28)	3.26($\pm$0.5)	2.953($\pm$0.35)

We create a contact network of only students with WIMOB and compare it with insights from contact networks created with EN. On average, we find the contact network constructed with WIMOB shows fewer average contacts, lower density and higher average shortest path (between reachable paths). Moreover, within WIMOB itself, characterizing all spaces reveals more contacts and shorter paths than only focusing on contacts in lectures. While the proportion of the largest component appears similar, note that with WIMOB, on average about only 70% of the students visit campus on a given week. We further inspect the disease–mitigating structural changes of the RI policy on the network. We observe that the changes across all metrics with EN appear to be more drastic than compared to WIMOB.

Table S3. Calibration outcomes with variations

	Calibrating on Positivity Rate at GT			Calibrating with University's Behavior	
Parameter	weeks $0-4$	weeks $5-9$	weeks $10-14$	UIUC	Berkeley
p	0.034 ± 0.007	0.073 ± 0.005	0.0024 ± 0.0003	0.024 ± 0.0009	0.041 ± 0.003
α	0.032 ± 0.0032	0.0042 ± 0.0006	0.0159 ± 0.002	0.0069 ± 0.0013	0.038 ± 0.006
I_0	0.012 ± 0.0009	0.00057 ± 0.00007	0.0030 ± 0.0007	0.0039 ± 0.0013	0.0048 ± 0.0003
Optimal r.m.s.e	0.0034	0.0007	0.0015	0.0028	0.0031
Effective R_0 (min - max), Fall 2020	$1.15-1.18$	$1.17-2.14$	$0.33-0.95$	$1.12-1.19$	$1.24-1.28$
Effective R_0 (min - max), Fall 2019	$2.87-5.68$	$5.15-12.93$	$1.27-1.36$	$3.35-5.35$	$3.32-7.00$

The results in the main paper use variables p, α, and I_0 as estimated by calibrating the simulation model on the first 5 weeks of positivity rates provided by GT surveillance for Fall 2020, while incorporating external cases from Fulton County. For sensitivity analyses, we perform calibrations on GT data for weeks $5-9$ and $10-14$. Additionally, we perform calibrations on first five weeks of UIUC and Berkeley positivity rate (along with data from their respective county). These parameters were found by validating the ABM on the remaining weeks of Fall 2020. To assess the basic reproductive number (R_0) of our ABM we study the first 4 weeks of the disease. We find the effective R_0 to be higher for Fall 2019 than Fall 2020 as the mobility behaviors between the 2 semesters was vastly different. Note, Fall 2020 exhibits only 39% of the mobility we observe in Fall 2019. In fact, the ABM is calibrated on Fall 2020, where behavior was subject to pandemic related closures, but in Fall 2019 the mobility was not hindered by any interventions. Thus, Fall 2019 reflects a counterfactual of Fall 2020 without any closures.

Table S4. Comparison of different $\mathsf{LC}_{\text{PRank}}$ policies in terms of controlling the disease and impacts on campus in Fall 2019; calibrated from week $0-4$ in Fall 2020 at GT

Behavioral Scenario	S1: Persistence			S2: Non-Res Avoidance			S3: Complete Avoidance		
Policy	RI	LC		RI	LC		RI	LC	
Budget	-	Mobility (95.5%)	Exposure Risk (18800)	-	Mobility (92.3%)	Exposure Risk (16900)	-	Mobility (69.2%)	Exposure Risk (12700)
Infection Reduction Outcomes									
Peak Infections (%)	$25.34(\pm12)$	$36.92(\pm14)^{**}$	$34.30(\pm13)^{**}$	$35.44(\pm10)$	$49.33(\pm11)^{**}$	$52.19(\pm10)^{**}$	$61.62(\pm7)$	$69.34(\pm5)^{**}$	$64.44(\pm6)^{**}$
Total Infections (%)	$6.99(\pm5)$	$10.63(\pm6)^{**}$	$8.19(\pm5)^{**}$	$14.88(\pm4)$	$13.96(\pm6)^{*}$	$15.67(\pm6)$	$33.00(\pm5)$	$33.4(\pm5)$	$26.94(\pm5)^{**}$
Internal Transmissions (%)	$17.13(\pm9)$	$22.62(\pm11)^{**}$	$21.01(\pm11)^{**}$	$27.58(\pm8)$	$35.35(\pm12)^{**}$	$39.20(\pm11)^{**}$	$54.00(\pm8)$	$70.89(\pm7)^{**}$	$60.90(\pm9)^{**}$
Burdens on Campus									
Locations Affected	58	18	19	58	38	50	58	192	124
Students Avoiding (%)	0	0	0	9.30	0.20	0.45	27.21	12.45	6.57
Completely Isolated on Campus (%)	5.42	8.40	8.40	5.95	5.72	5.71	7.09	5.18	5.23

Note that this table is the same as **??**. We repeat the results here for easier comparison of $\mathsf{LC}_{\text{PRank}}$ to other algorithms shown in Table S5, Table S6 and Table S7. Within each behavioral scenario, we perform the Kruskal-Wallis H-Test [19] to compare outcomes of $\mathsf{LC}_{\text{PRank}}$ with RI. We find that $\mathsf{LC}_{\text{PRank}}$ leads to significantly improved peak infection reduction and internal transmission. In terms of reduction in total infections, the outcomes are comparable in general but can vary by specific scenarios. In addition, every policy also exerts some burden on campus, either in terms of locations affected, students avoiding campus or isolation. We observe that $\mathsf{LC}_{\text{PRank}}$ policies focus on fewer locations (except in *S3*). Moreover, these policies affect fewer student's schedules and therefore fewer people avoid campus due to completely remote schedules. Finally, $\mathsf{LC}_{\text{PRank}}$ does not increase the percentage of people completely isolated on campus (p-value: < 0.01:*, < 0.001:**).

Table S5. Comparison of different $\mathsf{LC}_{\mathrm{BCen}}$ policies in terms of controlling the disease and impacts on campus in Fall 2019; calibrated from week $0-4$ in Fall 2020 at GT

Behavioral Scenario	S1: Persistence			S2: Non-Res Avoidance			S3: Complete Avoidance		
Policy	RI	$\mathsf{LC}_{\mathrm{BCen}}$		RI	$\mathsf{LC}_{\mathrm{BCen}}$		RI	$\mathsf{LC}_{\mathrm{BCen}}$	
Budget	-	Mobility (95.5%)	Exposure Risk (18800)	-	Mobility (92.3%)	Exposure Risk (16900)	-	Mobility (69.2%)	Exposure Risk (12700)
Infection Reduction Outcomes									
Peak Infections (%)	$25.34(\pm 12)$	$19.14(\pm 12)^{**}$	$30.93(\pm 13)^{**}$	$35.44(\pm 10)$	$30.79(\pm 13)^{**}$	$51.87(\pm 10)^{**}$	$61.62(\pm 7)$	$65.07(\pm 6)^{**}$	$61.38(\pm 7)$
Total Infections (%)	$6.99(\pm 5)$	$4.85(\pm 4)^{**}$	$7.74(\pm 5)$	$14.88(\pm 4)$	$7.76(\pm 5)^{**}$	$15.30(\pm 6)$	$33.00(\pm 5)$	$25.32(\pm 5)^{**}$	$22.08(\pm 6)^{**}$
Internal Transmissions (%)	$17.13(\pm 9)$	$11.96(\pm 9)^{**}$	$19.64(\pm 10)^{**}$	$27.58(\pm 8)$	$19.63(\pm 10)^{**}$	$38.74(\pm 11)^{**}$	$54.00(\pm 8)$	$63.29(\pm 8)^{**}$	$54.00(\pm 8)$
Burdens on Campus									
Locations Affected	58	18	19	58	38	50	58	192	124
Students Avoiding (%)	0	0	0	9.30	0.07	0.45	27.21	11.47	6.74
Completely Isolated on Campus (%)	5.42	8.63	8.63	5.95	5.49	5.47	7.09	5.15	5.19

Within each behavioral scenario, we perform the Kruskal-Wallis H-Test [19] to compare outcomes of $\mathsf{LC}_{\mathrm{BCen}}$ with RI. We find that $\mathsf{LC}_{\mathrm{BCen}}$ leads to significantly improved peak infection reduction and internal transmission, when designed with the exposure risk budget, but can be worse with the mobility budget. In terms of reduction in total infections, the outcomes are typically worse. In addition, every policy also exerts some burden on campus, either in terms of locations affected, students avoiding campus or isolation. We observe that $\mathsf{LC}_{\mathrm{BCen}}$ policies focus on fewer locations (except in *S3*). Moreover, these policies affect fewer student's schedules and therefore fewer people avoid campus due to completely remote schedules. Finally, $\mathsf{LC}_{\mathrm{LCen}}$ does not increase the percentage of people completely isolated on campus (p-value: < 0.01:*, < 0.001:**).

Table S6. Comparison of different $\mathsf{LC}_{\text{ECen}}$ policies in terms of controlling the disease and impacts on campus in Fall 2019; calibrated from week $0-4$ in Fall 2020 at GT

Behavioral Scenario	S1: Persistence			S2: Non-Res Avoidance			S3: Complete Avoidance		
Policy	RI	$\mathsf{LC}_{\text{ECen}}$		RI	$\mathsf{LC}_{\text{ECen}}$		RI	$\mathsf{LC}_{\text{ECen}}$	
Budget	-	Mobility (95.5%)	Exposure Risk (18800)	-	Mobility (92.3%)	Exposure Risk (16900)	-	Mobility (69.2%)	Exposure Risk (12700)
Infection Reduction Outcomes									
Peak Infections (%)	$25.34(\pm12)$	$36.15(\pm13)^{**}$	$36.13(\pm13)^{**}$	$35.44(\pm10)$	$44.52(\pm12)^{**}$	$51.33(\pm10)^{**}$	$61.62(\pm7)$	$65.13(\pm6)^{**}$	$62.15(\pm7)$
Total Infections (%)	$6.99(\pm5)$	$8.66(\pm6)^{**}$	$8.69(\pm6)^{**}$	$14.88(\pm4)$	$11.75(\pm6)^{**}$	$14.96(\pm6)$	$33.00(\pm5)$	$25.39(\pm5)^{**}$	$22.82(\pm6)^{**}$
Internal Transmissions (%)	$17.13(\pm9)$	$22.33(\pm11)^{**}$	$22.37(\pm11)^{**}$	$27.58(\pm8)$	$29.95(\pm12)^{*}$	$37.94(\pm11)^{**}$	$54.00(\pm8)$	$63.56(\pm8)^{**}$	$57.07(\pm10)^{**}$
Burdens on Campus									
Locations Affected	58	18	19	58	38	50	58	192	124
Students Avoiding (%)	0	0	0	9.30	0.20	0.55	27.21	13.11	6.96
Completely Isolated on Campus (%)	5.42	8.59	8.59	5.95	5.53	5.51	7.09	5.17	5.23

Within each behavioral scenario, we perform the Kruskal-Wallis H-Test [19] to compare outcomes of $\mathsf{LC}_{\text{ECen}}$ with RI. We find that $\mathsf{LC}_{\text{ECen}}$ leads to significantly improved peak infection reduction and internal transmission. In terms of reduction in total infections, the outcomes vary by specific scenarios. In addition, every policy also exerts some burden on campus, either in terms of locations affected, students avoiding campus or isolation. We observe that $\mathsf{LC}_{\text{ECen}}$ policies focus on fewer locations (except in $S3$). Moreover, these policies affect fewer student's schedules and therefore fewer people avoid campus due to completely remote schedules. Finally, $\mathsf{LC}_{\text{ECen}}$ does not increase the percentage of people completely isolated on campus (p-value: < 0.01:*, < 0.001:**).

Table S7. Comparison of different LC$_{\mathrm{LCen}}$ policies in terms of controlling the disease and impacts on campus in Fall 2019; calibrated from week $0-4$ in Fall 2020 at GT

Behavioral Scenario	S1: Persistence			S2: Non-Res Avoidance			S3: Complete Avoidance		
Policy	RI	LC$_{\mathrm{LCen}}$		RI	LC$_{\mathrm{LCen}}$		RI	LC$_{\mathrm{LCen}}$	
Budget	-	Mobility (95.5%)	Exposure Risk (18800)	-	Mobility (92.3%)	Exposure Risk (16900)	-	Mobility (69.2%)	Exposure Risk (12700)
Infection Reduction Outcomes									
Peak Infections (%)	$25.34(\pm12)$	$22.42(\pm13)^{**}$	$30.73(\pm13)^{**}$	$35.44(\pm10)$	$32.85(\pm13)^{*}$	$51.44(\pm10)^{**}$	$61.62(\pm7)$	$65.01(\pm6)^{**}$	$61.40(\pm7)$
Total Infections (%)	$6.99(\pm5)$	$5.48(\pm5)^{**}$	$7.64(\pm5)$	$14.88(\pm4)$	$8.23(\pm5)^{**}$	$15.03(\pm6)$	$33.00(\pm5)$	$25.33(\pm5)^{**}$	$21.98(\pm6)^{**}$
Internal Transmissions (%)	$17.13(\pm9)$	$13.79(\pm9)^{**}$	$19.37(\pm10)^{**}$	$27.58(\pm8)$	$20.86(\pm11)^{**}$	$38.08(\pm11)^{**}$	$54.00(\pm8)$	$63.28(\pm8)^{**}$	$55.28(\pm9)$
Burdens on Campus									
Locations Affected	58	18	19	58	38	50	58	192	124
Students Avoiding (%)	0	0	0	9.30	0.07	0.43	27.21	11.47	6.73
Completely Isolated on Campus (%)	5.42	8.63	8.63	5.95	5.49	5.47	7.09	5.15	5.20

Within each behavioral scenario, we perform the Kruskal-Wallis H-Test [19] to compare outcomes of LC$_{\mathrm{LCen}}$ with RI. We find that LC$_{\mathrm{LCen}}$ leads to significantly improved peak infection reduction and internal transmission. In terms of reduction in total infections, the outcomes are comparable in some scenarios but can vary in specific scenarios. In addition, every policy also exerts some burden on campus, either in terms of locations affected, students avoiding campus or isolation. We observe that LC$_{\mathrm{LCen}}$ policies focus on fewer locations (except in *S3*). Moreover, these policies affect fewer student's schedules and therefore fewer people avoid campus due to completely remote schedules. Finally, LC$_{\mathrm{LCen}}$ does not increase the percentage of people completely isolated on campus (p-value: <0.01:*, <0.001:**).

Table S8. Comparison of different LC$_{\mathrm{PRank}}$ policies in terms of controlling the disease and impacts on campus in Fall 2019; calibrated from week $5-9$ in Fall 2020 at GT

Behavioral Scenario	S1: Persistence			S2: Non-Res Avoidance			S3: Complete Avoidance		
Policy	Broad	LC$_{\mathrm{PRank}}$		RI	LC$_{\mathrm{PRank}}$		RI	LC$_{\mathrm{PRank}}$	
Budget	-	Mobility (95.5%)	Exposure Risk (18800)	-	Mobility (92.3%)	Exposure Risk (16900)	-	Mobility (69.2%)	Exposure Risk (12700)
Infection Reduction Outcomes									
Peak Infections (%)	$20.10(\pm4)$	$25.60(\pm3)^{**}$	$25.63(\pm3)^{**}$	$31.25(\pm3)$	$42.32(\pm4)^{**}$	$47.29(\pm4)^{**}$	$62.35(\pm2)$	$88.87(\pm2)^{**}$	$76.89(\pm3)^{**}$
Total Infections (%)	$8.89(\pm2)$	$10.50(\pm3)^{**}$	$9.70(\pm3)^{**}$	$20.26(\pm2)$	$20.02(\pm3)$	$23.71(\pm4)^{**}$	$46.72(\pm2)$	$67.92(\pm4)^{**}$	$51.30(\pm4)^{**}$
Internal Transmissions (%)	$9.97(\pm2)$	$11.51(\pm2)^{**}$	$10.95(\pm2)^{**}$	$21.84(\pm2)$	$22.51(\pm3)$	$26.64(\pm3)^{**}$	$49.80(\pm2)$	$74.96(\pm3)^{**}$	$56.89(\pm4)^{**}$

Within each behavioral scenario, we perform the Kruskal-Wallis H-Test [19] to compare outcomes of LC$_{\mathrm{PRank}}$ with RI. We find that LC$_{\mathrm{PRank}}$ leads to significantly improved peak infection reduction and internal transmission. In terms of reduction in total infections, the outcomes are better in general but can be comparable in specific scenarios. The burden exerted on campus is the same as structural impacts of LC$_{\mathrm{PRank}}$ (Table S4). (p-value: <0.01:*, <0.001:**).

Table S9. Comparison of different $\mathsf{LC}_{\text{PRank}}$ policies in terms of controlling the disease and impacts on campus in Fall 2019; calibrated from week $10-14$ in Fall 2020 at GT

Behavioral Scenario	S1: Persistence			S2: Non-Res Avoidance			S3: Complete Avoidance		
Policy	Broad	$\mathsf{LC}_{\text{PRank}}$		RI	$\mathsf{LC}_{\text{PRank}}$		RI	$\mathsf{LC}_{\text{PRank}}$	
Budget	-	Mobility (95.5%)	Exposure Risk (18800)	-	Mobility (92.3%)	Exposure Risk (16900)	-	Mobility (69.2%)	Exposure Risk (12700)
Infection Reduction Outcomes									
Peak Infections (%)	$-1.75(\pm 8)$	$3.65(\pm 8)^{**}$	$-1.95(\pm 8)$	$3.88(\pm 8)$	$-2.24(\pm 8)^{**}$	$-2.06(\pm 8)^{**}$	$20.39(\pm 7)$	$7.57(\pm 8)^{**}$	$2.81(\pm 8)^{**}$
Total Infections (%)	$3.93(\pm 9)$	$10.36(\pm 8)^{**}$	$5.13(\pm 9)$	$9.87(\pm 8)$	$6.36(\pm 9)^{**}$	$6.48(\pm 9)^{**}$	$26.02(\pm 7)$	$16.37(\pm 8)^{**}$	$11.80(\pm 8)^{**}$
Internal Transmissions (%)	$42.33(\pm 10)$	$61.15(\pm 7)^{**}$	$56.25(\pm 8)^{**}$	$49.83(\pm 9)$	$67.10(\pm 6)^{**}$	$69.10(\pm 6)^{**}$	$74.74(\pm 5)$	$84.80(\pm 3)^{**}$	$79.90(\pm 4)^{**}$

Within each behavioral scenario, we perform the Kruskal-Wallis H-Test [19] to compare outcomes of $\mathsf{LC}_{\text{PRank}}$ with RI. We find that $\mathsf{LC}_{\text{PRank}}$ leads to significantly improved peak infection reduction and internal transmission. In terms of reduction in total infections, the outcomes are better in general but can be comparable in specific scenarios. The burden exerted on campus is the same as structural impacts of $\mathsf{LC}_{\text{PRank}}$ (Table S4). (p-value: < 0.01:*, < 0.001:**).

Table S10. Comparison of different $\mathsf{LC}_{\text{PRank}}$ policies in terms of controlling the disease and impacts on campus in Fall 2019; calibrated from week $0-4$ in Fall 2020 at UIUC

Behavioral Scenario	S1: Persistence			S2: Non-Res Avoidance			S3: Complete Avoidance		
Policy	Broad	$\mathsf{LC}_{\text{PRank}}$		RI	$\mathsf{LC}_{\text{PRank}}$		RI	$\mathsf{LC}_{\text{PRank}}$	
Budget	-	Mobility (95.5%)	Exposure Risk (18800)	-	Mobility (92.3%)	Exposure Risk (16900)	-	Mobility (69.2%)	Exposure Risk (12700)
Infection Reduction Outcomes									
Peak Infections (%)	$41.40(\pm3)$	$60.44(\pm2)^{**}$	$59.52(\pm2)^{**}$	$49.75(\pm2)$	$74.22(\pm2)^{**}$	$76.44(\pm2)^{**}$	$78.14(\pm1)$	$85.81(\pm1)^{**}$	$83.71(\pm1)^{**}$
Total Infections (%)	$18.46(\pm3)$	$27.12(\pm3)^{**}$	$25.25(\pm3)^{**}$	$27.09(\pm3)$	$38.00(\pm4)^{**}$	$40.68(\pm4)^{**}$	$51.97(\pm3)$	$59.93(\pm5)^{**}$	$54.07(\pm5)^{**}$
Internal Transmissions (%)	$28.22(\pm3)$	$40.93(\pm3)^{**}$	$39.09(\pm3)^{**}$	$37.89(\pm3)$	$58.47(\pm2)^{**}$	$65.45(\pm2)^{**}$	$68.04(\pm2)$	$86.45(\pm1)^{**}$	$80.08(\pm1)^{**}$

Within each behavioral scenario, we perform the Kruskal-Wallis H-Test [19] to compare outcomes of $\mathsf{LC}_{\text{PRank}}$ with RI. We find that $\mathsf{LC}_{\text{PRank}}$ leads to significantly improved peak infection reduction, internal transmission and total infections. The burden exerted on campus is the same as structural impacts of $\mathsf{LC}_{\text{PRank}}$ (Table S4). (p-value: < 0.01:*, < 0.001:**).

Table S11. Comparison of different $\mathsf{LC}_{\text{PRank}}$ policies in terms of controlling the disease and impacts on campus in Fall 2019; calibrated from week $0-4$ in Fall 2020 at UC Berkeley

Behavioral Scenario	S1: Persistence			S2: Non-Res Avoidance			S3: Complete Avoidance		
Policy	Broad	$\mathsf{LC}_{\text{PRank}}$		RI	$\mathsf{LC}_{\text{PRank}}$		RI	$\mathsf{LC}_{\text{PRank}}$	
Budget	-	Mobility (95.5%)	Exposure Risk (18800)	-	Mobility (92.3%)	Exposure Risk (16900)	-	Mobility (69.2%)	Exposure Risk (12700)
Infection Reduction Outcomes									
Peak Infections (%)	$29.13(\pm3)$	$36.46(\pm5)^{**}$	$36.34(\pm5)^{**}$	$38.83(\pm3)$	$54.95(\pm4)^{**}$	$58.88(\pm4)^{**}$	$66.69(\pm2)$	$78.18(\pm1)^{**}$	$77.65(\pm2)^{**}$
Total Infections (%)	$6.34(\pm3)$	$8.59(\pm3)^{**}$	$7.28(\pm3)^{**}$	$14.71(\pm3)$	$13.18(\pm4)^{**}$	$14.83(\pm4)$	$33.86(\pm4)$	$33.98(\pm5)$	$27.10(\pm5)^{**}$
Internal Transmissions (%)	$15.99(\pm3)$	$20.43(\pm4)^{**}$	$19.17(\pm4)^{**}$	$27.01(\pm3)$	$34.60(\pm4)^{**}$	$38.78(\pm4)^{**}$	$55.01(\pm2)$	$74.65(\pm2)^{**}$	$63.57(\pm3)^{**}$

Within each behavioral scenario, we perform the Kruskal-Wallis H-Test [19] to compare outcomes of $\mathsf{LC}_{\text{PRank}}$ with RI. We find that $\mathsf{LC}_{\text{PRank}}$ leads to significantly improved peak infection reduction, internal transmission and total infections. The burden exerted on campus is the same as structural impacts of $\mathsf{LC}_{\text{PRank}}$ (Table S4). (p-value: < 0.01:*, < 0.001:**).

3 FIGURE CAPTIONS

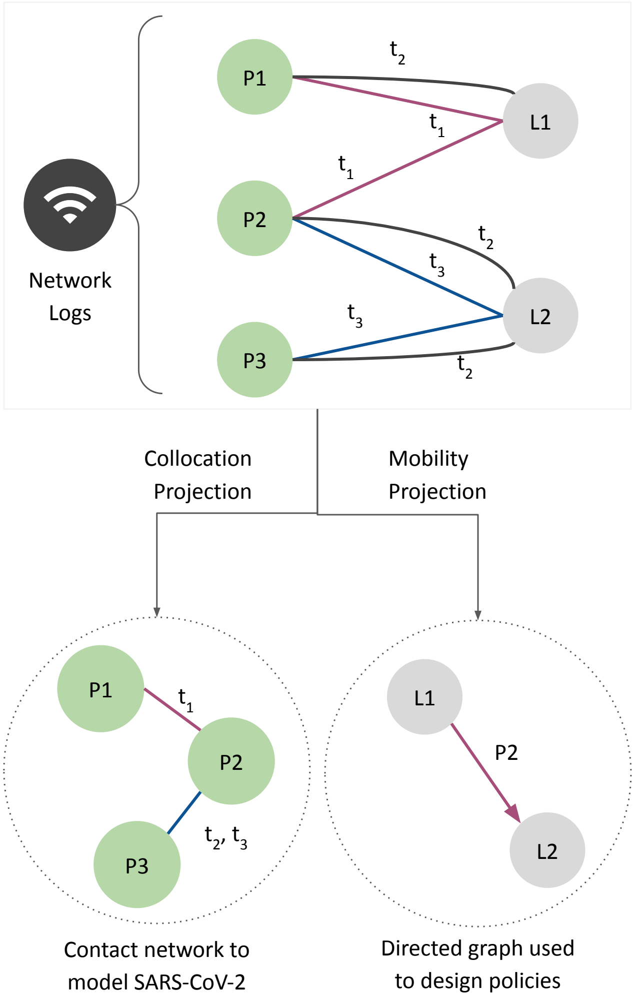

Figure S1: In a managed network, SNMP updates the logs by describing device association to an AP at a certain timestamp. WIMOB mines these logs to characterize mobility as a bipartite graph. The nodes are partitioned to describe people nodes (e.g., $P1$, $P2$) connected to locations nodes (e.g., $L1$, $L2$). Every edge across the partition describes people visiting locations on campus during different times (e.g., t_1, t_2). Projecting the bipartite on people nodes helps construct a contact network (e.g., $P1$ and $P2$ were collocated at $L1$ at t_1), while projecting it on locations helps construct a directed movement graph ($P2$ dwelled at $L1$ and then at $L2$).

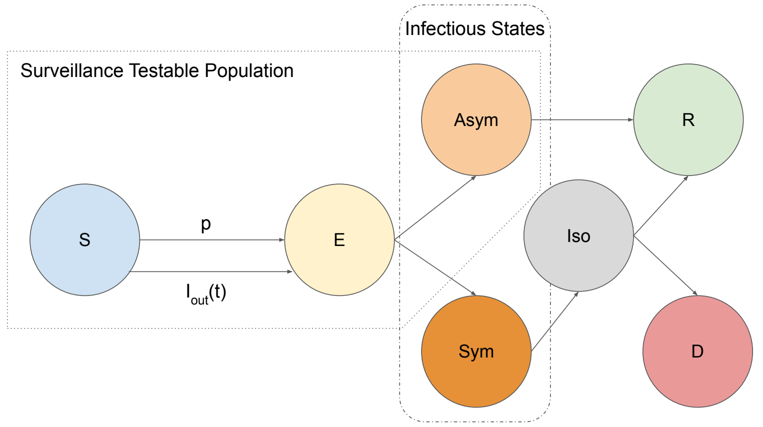

Figure S2a

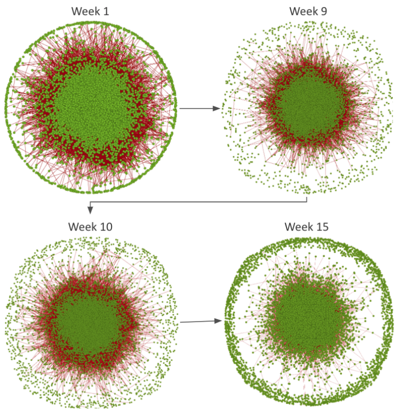

Figure S2b

Figure S2: (a) The schematic of the compartments in our modified SEIR model. By the design of the GT surveillance testing [28, 15], the total testable population is defined as the summation of *susceptible*, *exposed*, and *asymptomatic*. Infectious persons are in either *symptomatic* or *symptomatic*. For every effective edge in the mobility network, a susceptible individual that is exposed to an infectious person becomes infected with probability p. Individuals may also get infected due to an exposure not captured by the WiMob network which occurs with probability $I_{out}(t)$ on day t. account for new infected cases. (b) The mobility behavior represented by WiMob changes every day of the semester (shown weekly here). The contact network constructed from WiMob forms the underlying contact structure of the ABM.

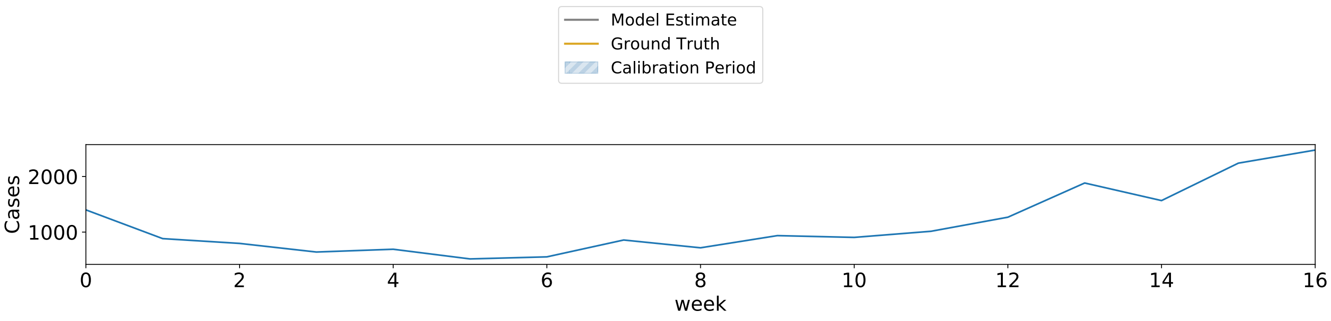

Figure S3a: External cases

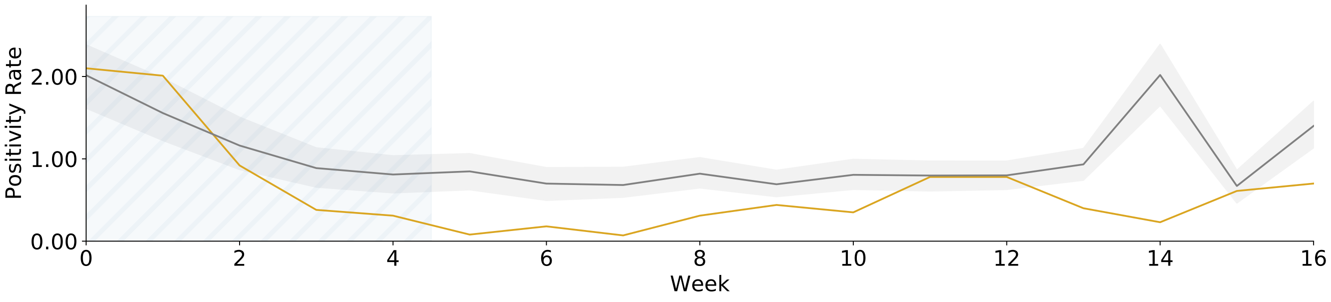

Figure S3b: Calibrating on the weeks 0-4

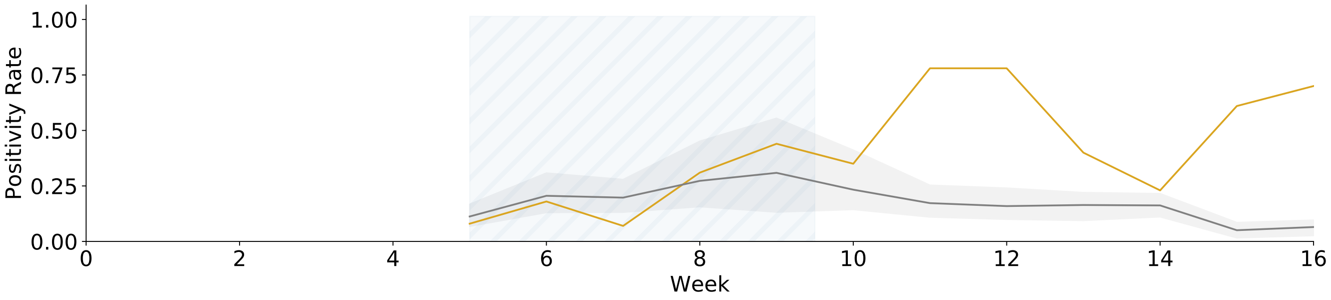

Figure S3c: Calibrating on the weeks 5-9

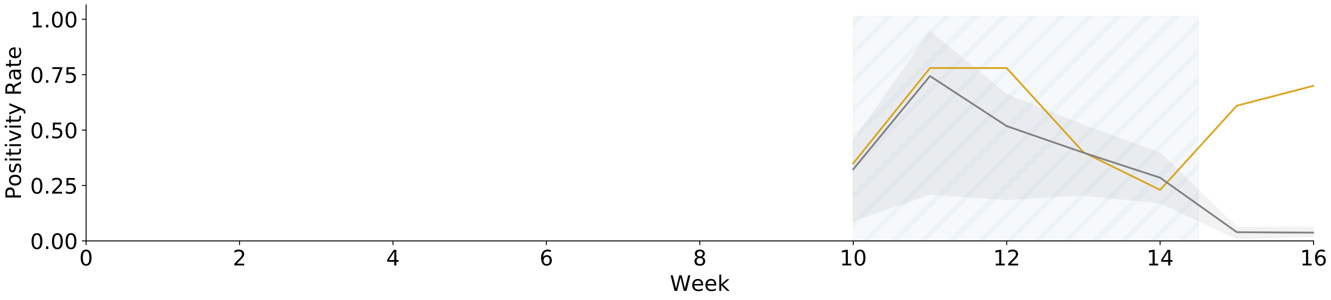

Figure S3d: Calibrating on the weeks 10-14

Figure S3: We calibrate ABM on positivity rates from Fall 2020 at GT. The objective function of the calibration is to minimize the r.m.s.e. with the weekly average of positivity rate obtained from surveillance testing results at GT [15]. (a) The parameter that determines external transmission of infections on a given day, $I_{out}(t)$, is a function of cases in Fulton county (where GT is located). (b) The models discussed in the main paper are calibrated using the first 5 weeks of data. We illustrate the output for a range of parameters that incorporate quantitative uncertainty, i.e., within 40% of the r.m.s.e. (c, d) illustrate calibration on the second period of 5 weeks and third period of 5 weeks respectively. These only show the optimal parameter output. The shaded region around the lines show the 2.5^{th} and 97.5^{th} percentile.

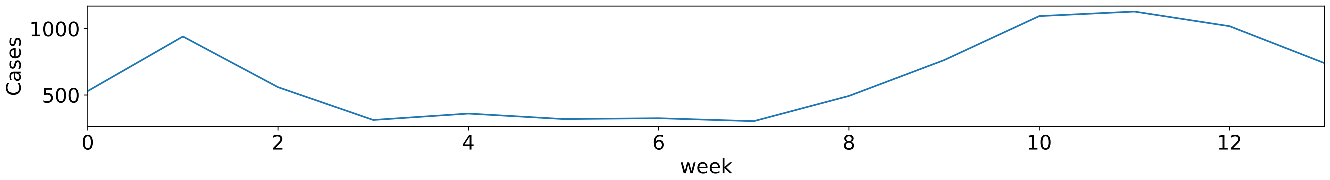

Figure S4a: External cases

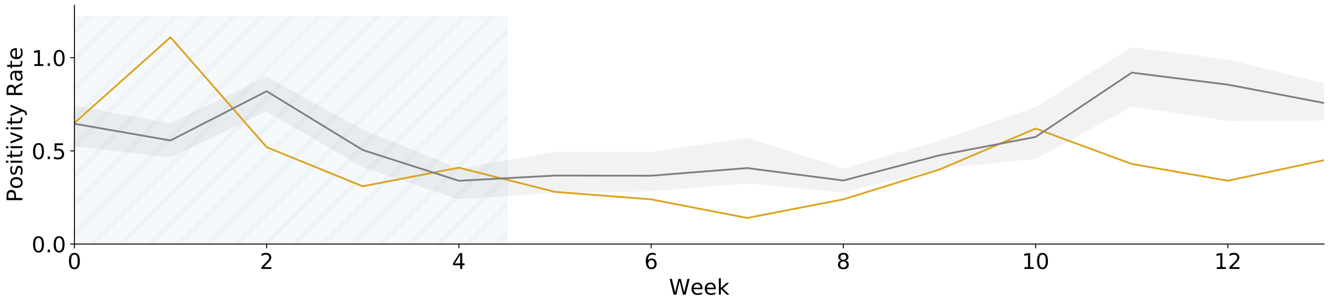

Figure S4b: Calibrating on the weeks 0-4 at UIUC

Figure S4: We calibrate ABM on positivity rates from first 5 weeks of Fall 2020 at UIUC. The objective function of the calibration is to minimize the r.m.s.e. with the weekly average of positivity rate obtained from surveillance testing results at GT [15]. (a) The parameter that determines external transmission of infections on a given day, $I_{out}(t)$, is a function of cases in Champaign county (where UIUC is located). (b) We illustrate the output for a range of parameters that incorporate quantitative uncertainty, i.e., within 40% of the r.m.s.e. The shaded region around the lines show the 2.5^{th} and 97.5^{th} percentile.

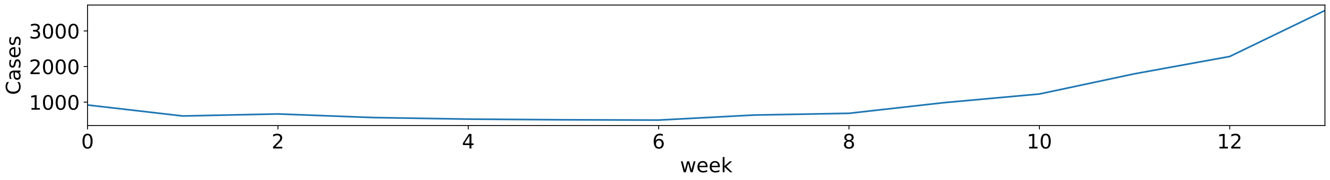

Figure S5a: External cases

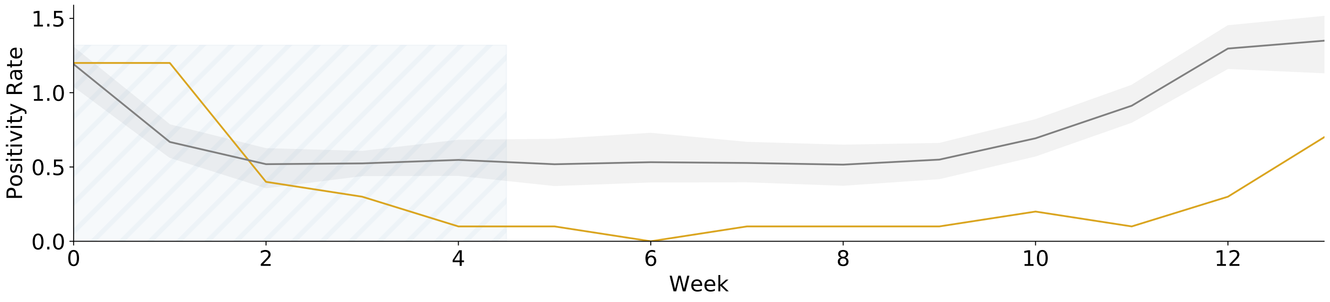

Figure S5b: Calibrating on the weeks 0-4 at UC Berkeley

Figure S5: We calibrate ABM on positivity rates from first 5 weeks of Fall 2020 at UC Berkeley. The objective function of the calibration is to minimize the r.m.s.e. with the weekly average of positivity rate obtained from surveillance testing results at GT [15]. (a) The parameter that determines external transmission of infections on a given day, $I_{out}(t)$, is a function of cases in Alameda county (where UIUC is located). (b) We illustrate the output for a range of parameters that incorporate quantitative uncertainty, i.e., within 40% of the r.m.s.e. The shaded region around the lines show the 2.5^{th} and 97.5^{th} percentile.

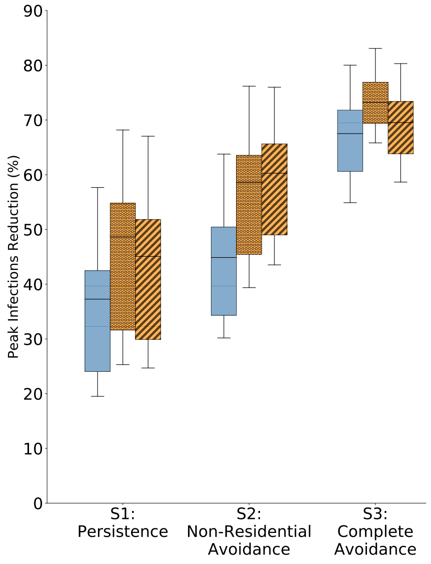

Figure S6a: Peak Transmission Reduction Percentage ($\mathsf{LC}_{\mathrm{PRank}}$)

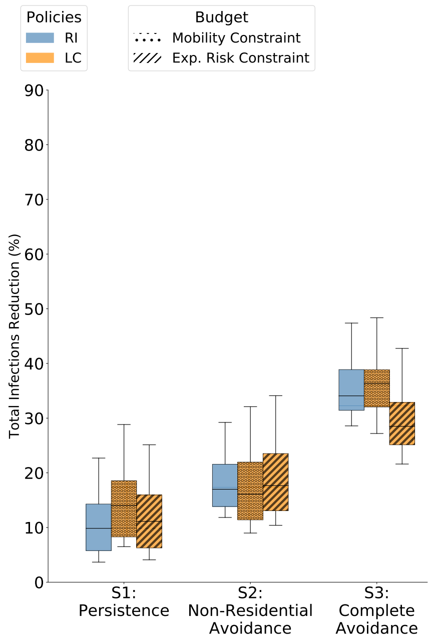

Figure S6b: Total Infections Reduction Percentage ($\mathsf{LC}_{\mathrm{PRank}}$)

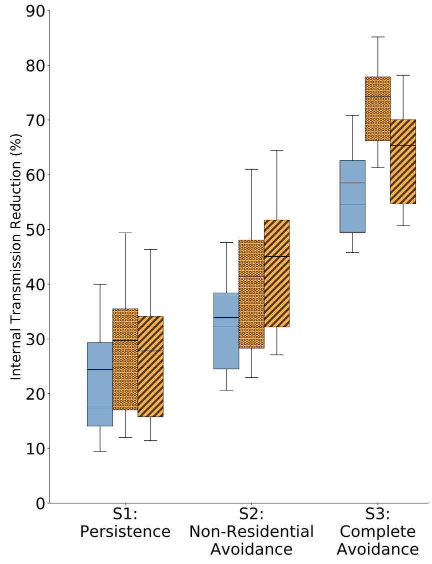

Figure S6c: Internal Transmission Reduction Percentage ($\mathsf{LC}_{\mathrm{PRank}}$)

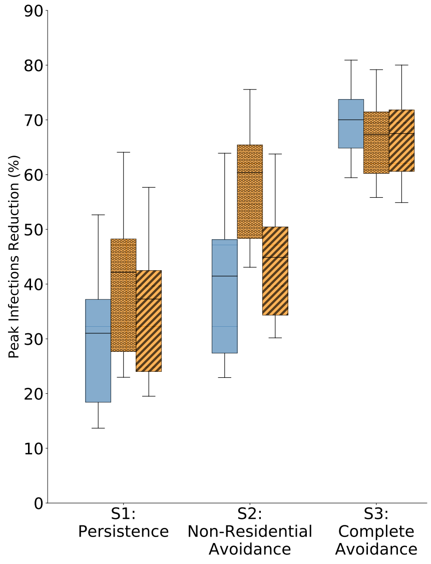

Figure S6d: Peak Infection Reduction Percentage ($\mathsf{LC}_{\mathrm{BCen}}$)

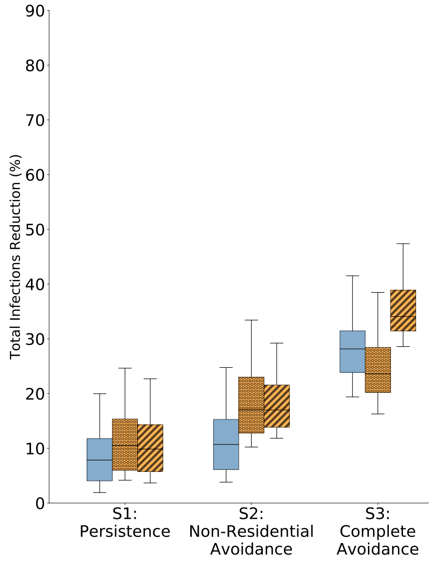

Figure S6e: Total Infection Reduction Percentage ($\mathsf{LC}_{\mathrm{BCen}}$)

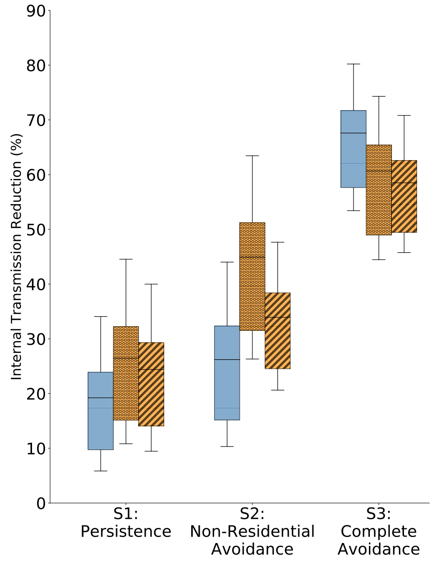

Figure S6f: Internal Transmission Reduction Percentage ($\mathsf{LC}_{\mathrm{BCen}}$)

Figure S6: Disease control outcomes in Fall 2019 for different algorithms of LC with the ABM is calibrated on weeks $0-4$ of Fall 2020 at GT. $(a-c)$ Comparison of RI with $\mathsf{LC}_{\mathrm{PRank}}$. Under all behavioral scenarios, for peak infection reduction (b) and internal transmission reduction (c), $\mathsf{LC}_{\mathrm{PRank}}$ shows better disease control outcomes than RI. For total infection reduction (b), $\mathsf{LC}_{\mathrm{PRank}}$ is better in *S1*, worse in *S3* when designed within an exposure risk budget, and comparable in others. $(d-f)$ Comparison of RI with $\mathsf{LC}_{\mathrm{BCen}}$. Under all behavioral scenarios, for peak infection reduction (d) and internal transmission reduction (f) $\mathsf{LC}_{\mathrm{BCen}}$ is better when designed within an exposure risk budget. For total infection reduction (e), $\mathsf{LC}_{\mathrm{BCen}}$ is always worse than RI

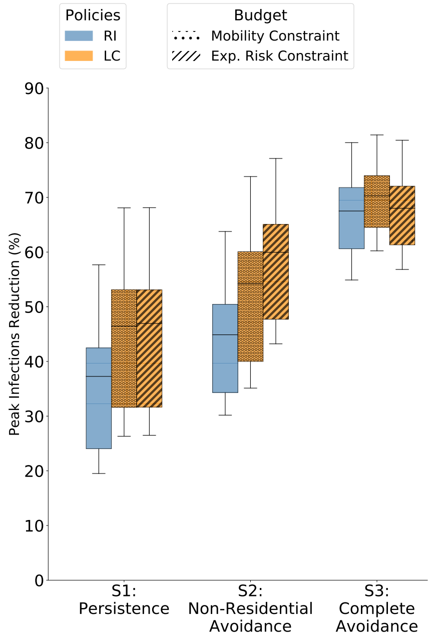

Figure S7a: Peak Infection Reduction Percentage (LC_{ECen})

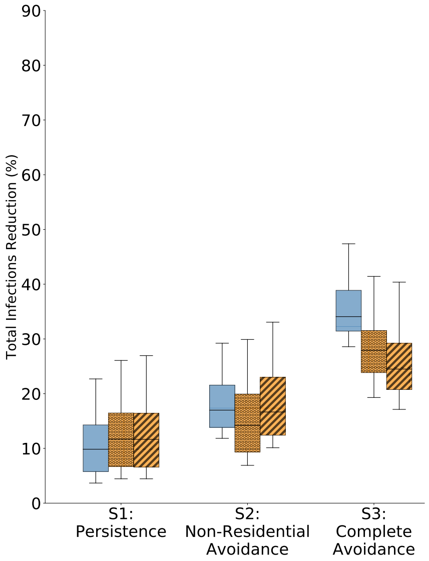

Figure S7b: Total Infection Reduction Percentage (LC_{ECen})

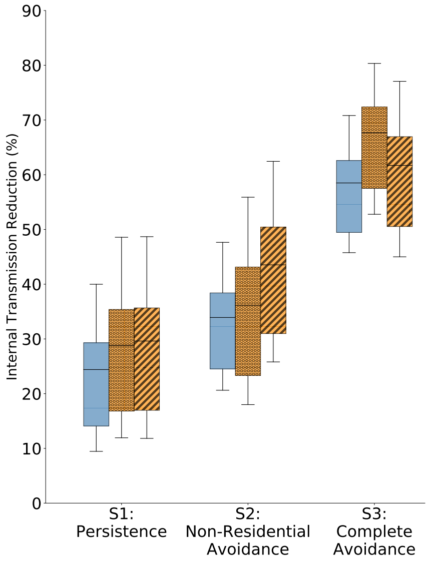

Figure S7c: Internal Transmission Reduction Percentage (LC_{ECen})

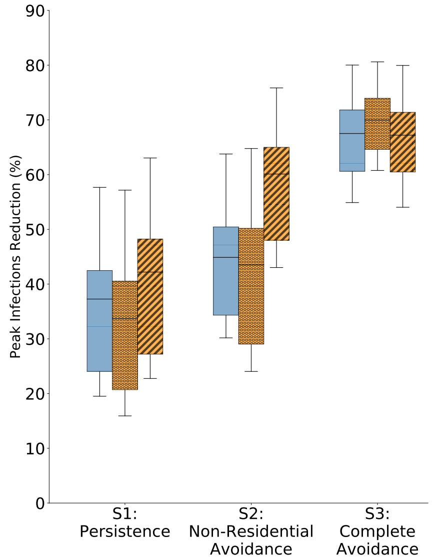

Figure S7d: Peak Infection Reduction Percentage (LC_{LCen})

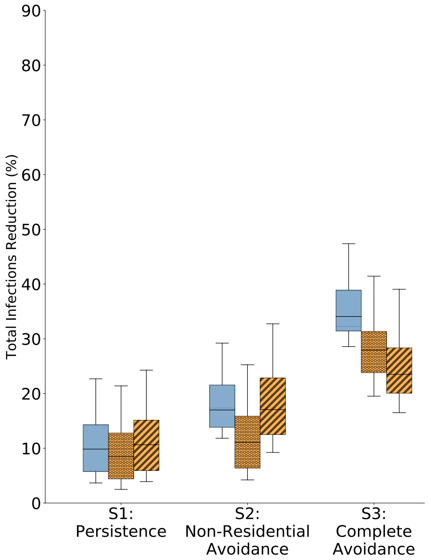

Figure S7e: Total Infection Reduction Percentage (LC_{LCen})

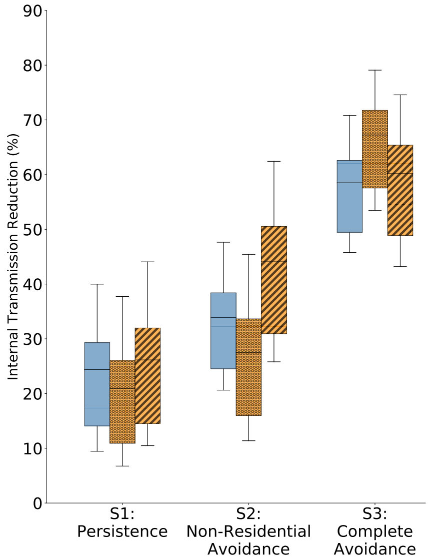

Figure S7f: Internal Transmission Reduction Percentage (LC_{LCen})

Figure S7: Disease control outcomes in Fall 2019 for different algorithms of LC with the ABM is calibrated on weeks $0-4$ of Fall 2020 at GT. $(a-c)$ Comparison of RI with LC_{ECen}. Under all behavioral scenarios, for peak infection reduction (b) and internal transmission reduction (c), LC_{ECen} shows better disease control outcomes than RI. For total infection reduction (b), LC_{ECen} is better in *S1* and worse in *S3* when designed within an exposure risk budget. $(d-f)$ Comparison of RI with LC_{ECen}. Under all behavioral scenarios, for peak infection reduction (d) and internal transmission reduction (f), LC_{ECen} shows better disease control outcomes than RI. For total infection reduction (e), LC_{ECen} is better in *S1* and worse in *S3* when designed within an exposure risk budget.

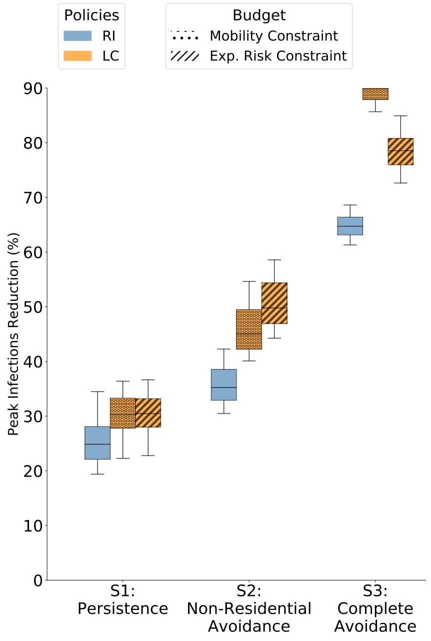

Figure S8a: Peak Transmission Reduction Percentage (Weeks 5 - 9)

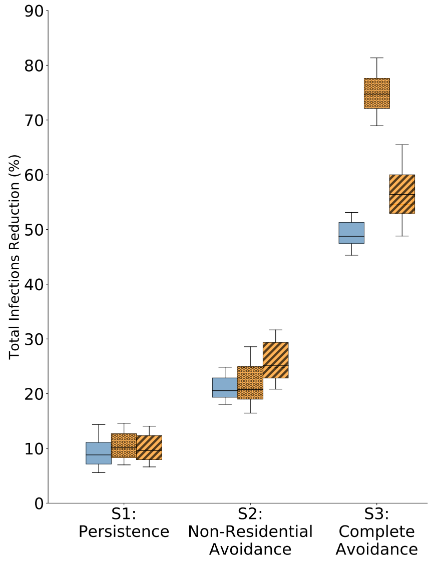

Figure S8b: Total Infections Reduction Percentage (Weeks 5 - 9)

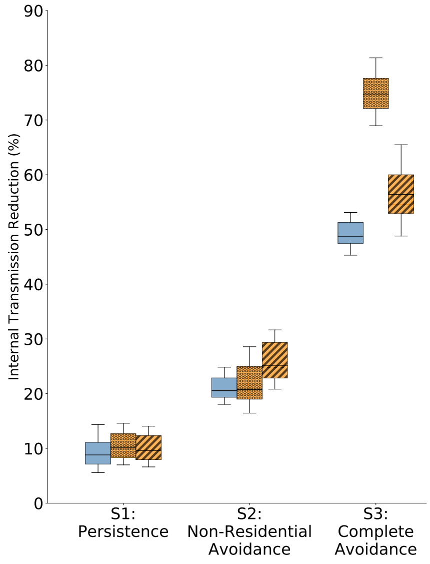

Figure S8c: Internal Transmission Reduction Percentage (Weeks 5 - 9)

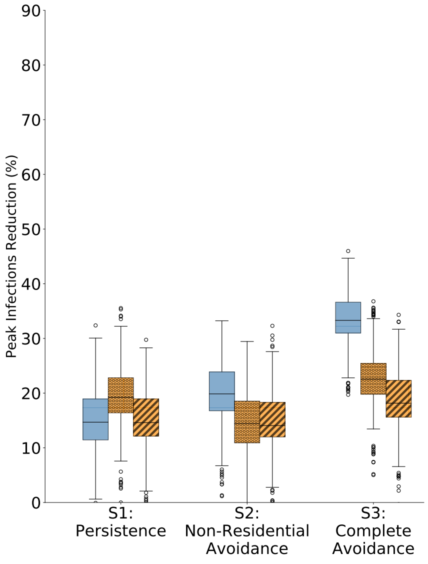

Figure S8d: Peak Transmission Reduction Percentage (Weeks 10 - 14)

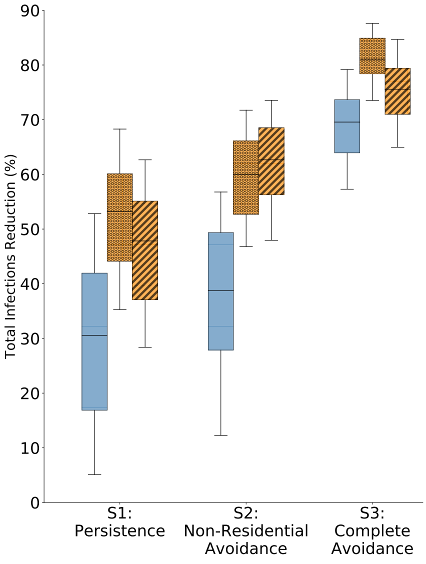

Figure S8e: Total Infections Reduction Percentage (Weeks 10 - 14)

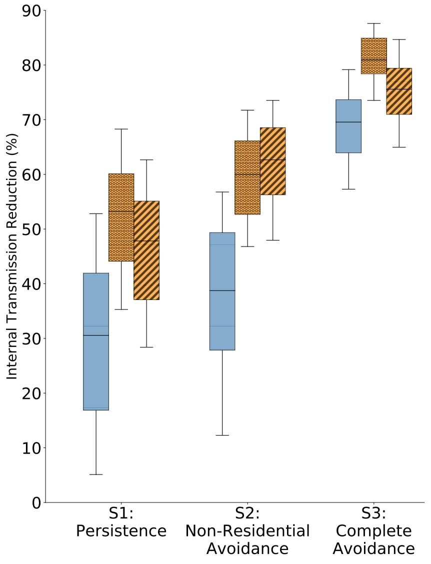

Figure S8f: Internal Transmission Reduction Percentage (Weeks 10 - 14)

Figure S8: Disease control outcomes in Fall 2019 for $\mathsf{LC}_{\mathrm{PRank}}$. $(a-c)$ The ABM was calibrated on weeks $5-9$ of Fall 2020 at GT. Under all behavioral scenarios, for all outcomes, $\mathsf{LC}_{\mathrm{PRank}}$ is better than RI. $(d-f)$ The ABM was calibrated on weeks $10-14$ of Fall 2020 at GT. Under all behavioral scenarios, for all outcomes, $\mathsf{LC}_{\mathrm{PRank}}$ is better than RI.

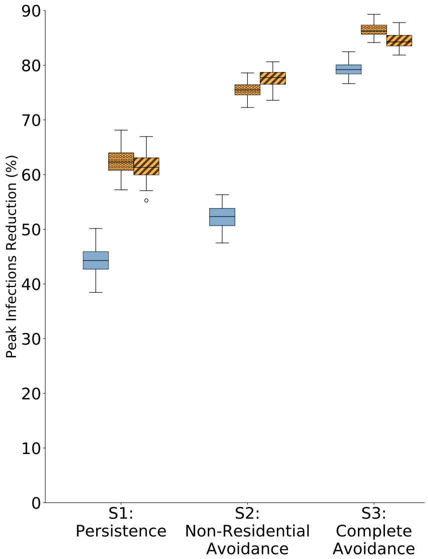

Figure S9: Peak Transmission Reduction Percentage (UIUC)

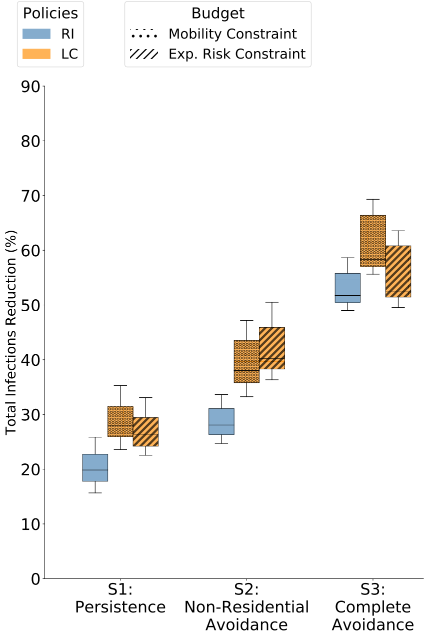

Figure S9a: Total Infections Reduction Percentage (UIUC)

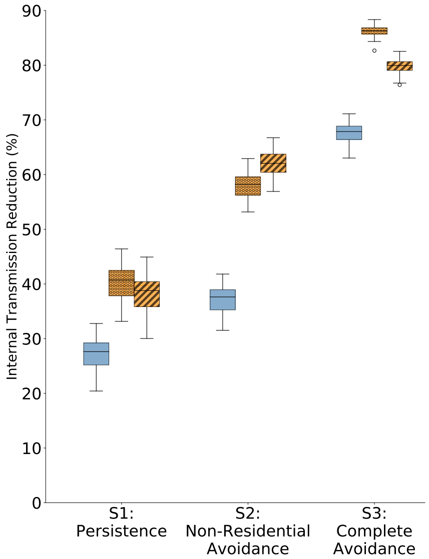

Figure S9b: Internal Transmission Reduction Percentage (UIUC)

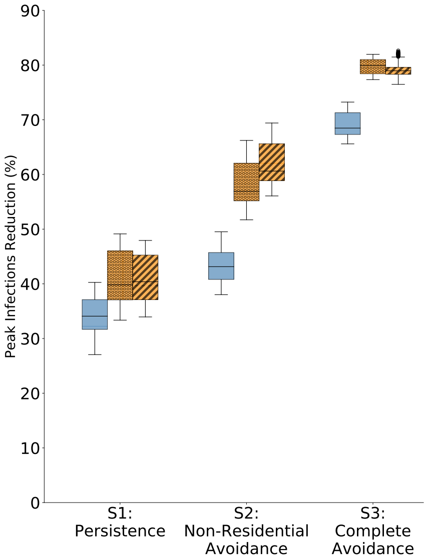

Figure S9c: Peak Transmission Reduction Percentage (UCB)

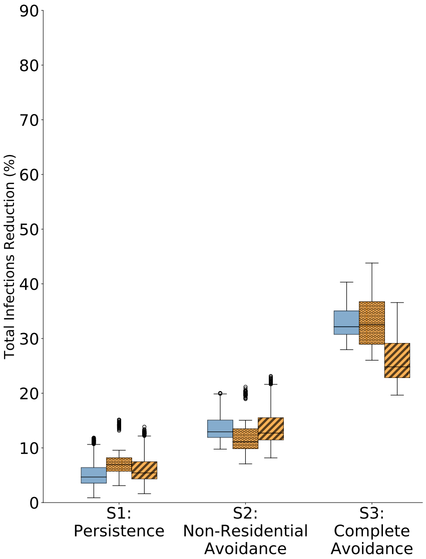

Figure S9d: Total Infections Reduction Percentage (UCB)

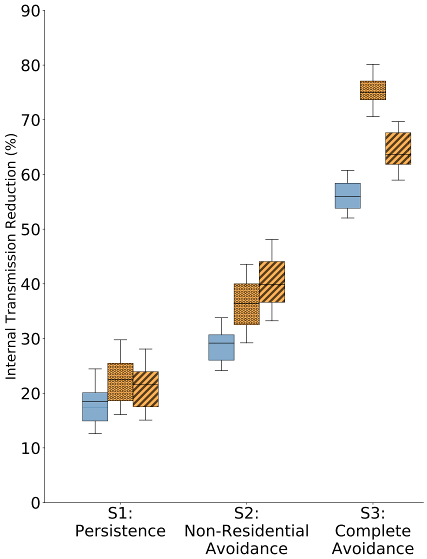

Figure S9e: Internal Transmission Reduction Percentage (UCB)

Figure S9: Disease control outcomes in Fall 2019 for $\mathsf{LC}_{\text{PRank}}$. $(a - c)$ The ABM was calibrated on weeks $0 - 4$ of Fall 2020 at UIUC. Under all behavioral scenarios, for all outcomes, $\mathsf{LC}_{\text{PRank}}$ is better than RI. $(d - f)$ The ABM was calibrated on weeks $0 - 4$ of Fall 2020 at UC Berkeley. Under all behavioral scenarios, for all outcomes, $\mathsf{LC}_{\text{PRank}}$ is better than RI.

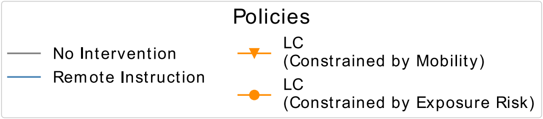

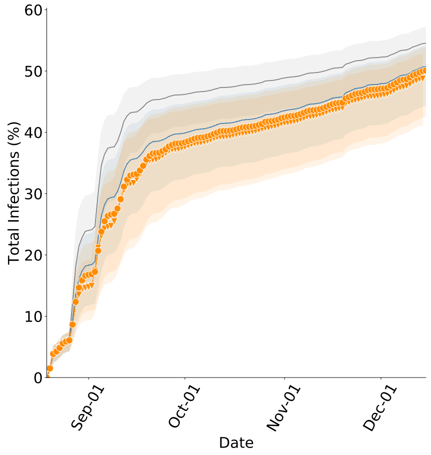

Figure S10a: S1 (LC$_{\text{PRank}}$)

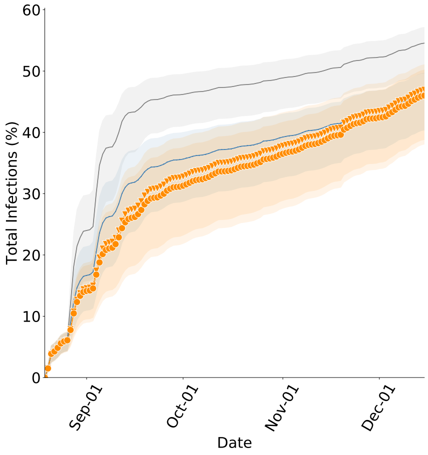

Figure S10b: S2 (LC$_{\text{PRank}}$)

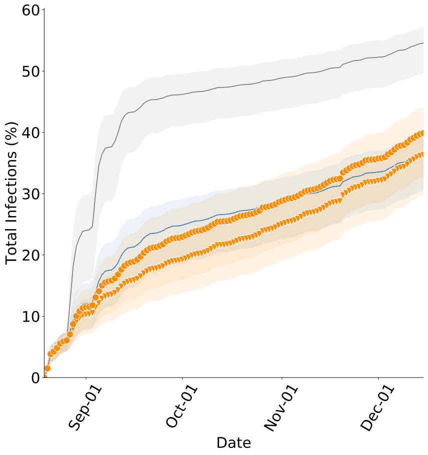

Figure S10c: S3 (LC$_{\text{PRank}}$)

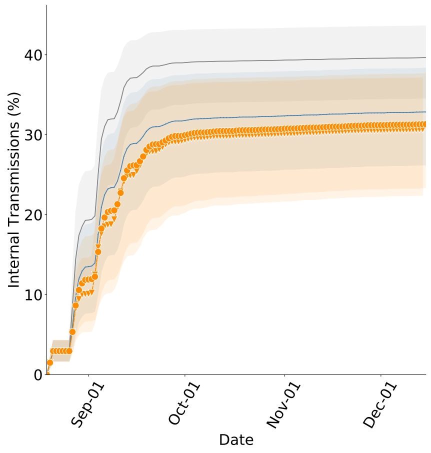

Figure S10d: S1 (LC$_{\text{PRank}}$)

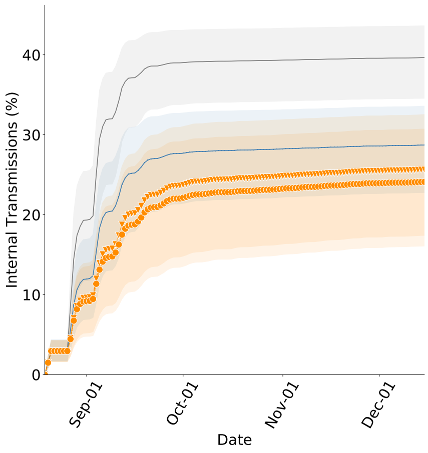

Figure S10e: S2 (LC$_{\text{PRank}}$)

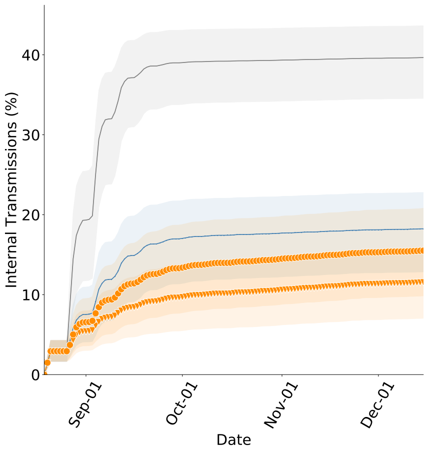

Figure S10f: S3 (LC$_{\text{PRank}}$)

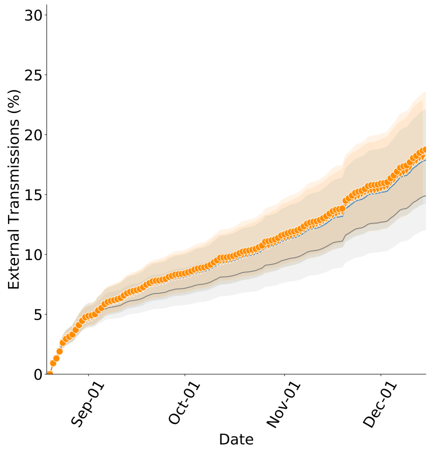

Figure S10g: S1 (LC$_{\text{PRank}}$)

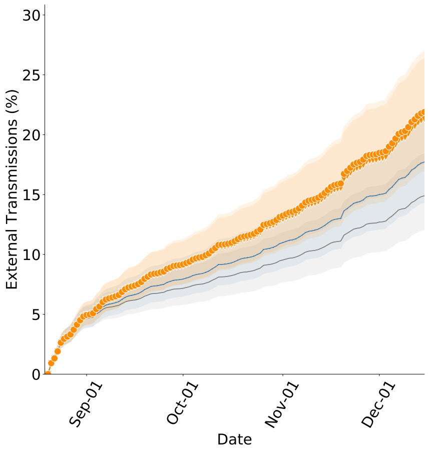

Figure S10h: S2 (LC$_{\text{PRank}}$)

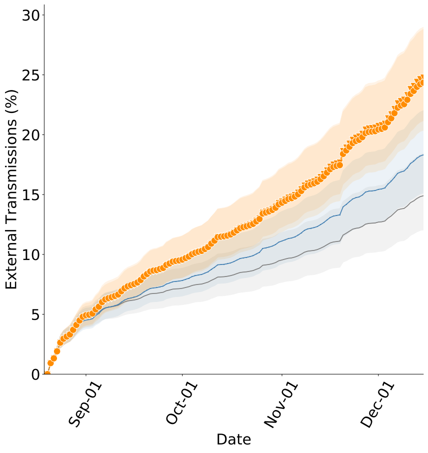

Figure S10i: S3 (LC$_{\text{PRank}}$)

Figure S10: Cumulative infections in Fall 2019 while comparing RI and LC$_{\text{PRank}}$ with ABM calibrated on weeks $0-4$ of Fall 2020, GT. The bands show the 2.75^{th} and 97.25^{th} percentile. $(a-c)$ Total infections of interventions is lower than no-intervention and is lowest in the *S3* scenario. In this behavioral scenario, the mobility budget is 69% of what it would be without interventions, and therefore the transmissions are also contained. In comparison, in Fall 2020, we saw far fewer infections which is because the mobility was 39% of that in Fall 2019. $(d-f)$ Internal transmissions are lower with LC$_{\text{PRank}}$ in comparison to RI. $(g-i)$ External transmissions are higher with LC$_{\text{PRank}}$ in comparison to RI. Since internal transmission is controlled, more individuals remain susceptible to infections from outside campus.

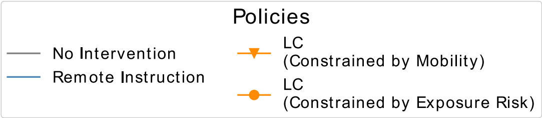

Figure S11a: S1 ($\mathsf{LC}_{\mathrm{BCen}}$)

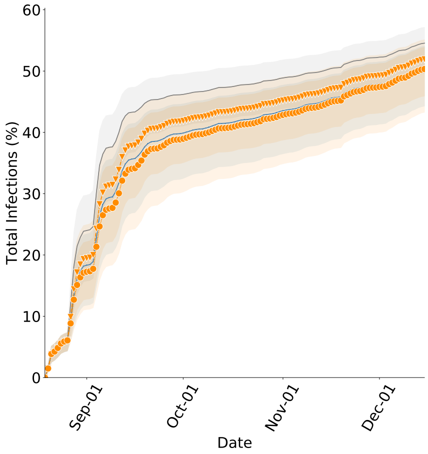

Figure S11b: S2 ($\mathsf{LC}_{\mathrm{BCen}}$)

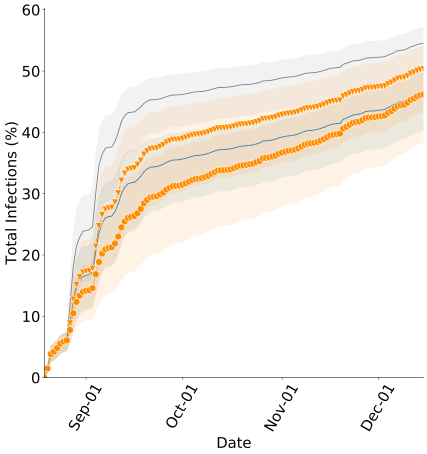

Figure S11c: S3 ($\mathsf{LC}_{\mathrm{BCen}}$)

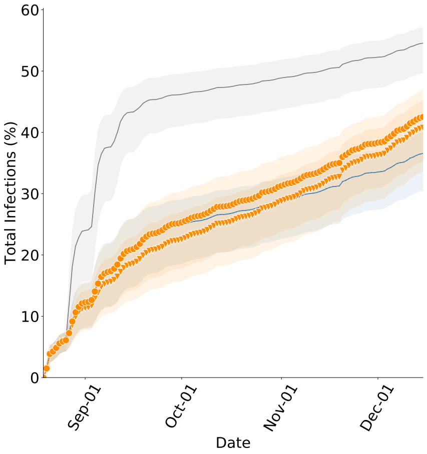

Figure S11d: S1 ($\mathsf{LC}_{\mathrm{BCen}}$)

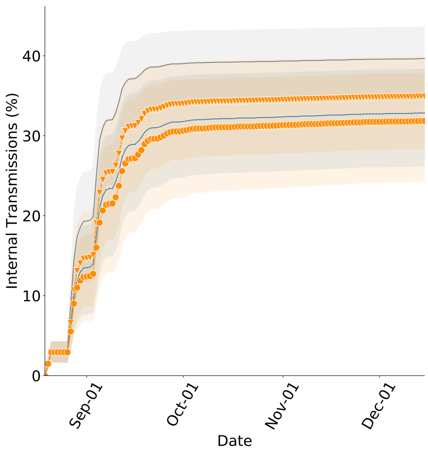

Figure S11e: S2 ($\mathsf{LC}_{\mathrm{BCen}}$)

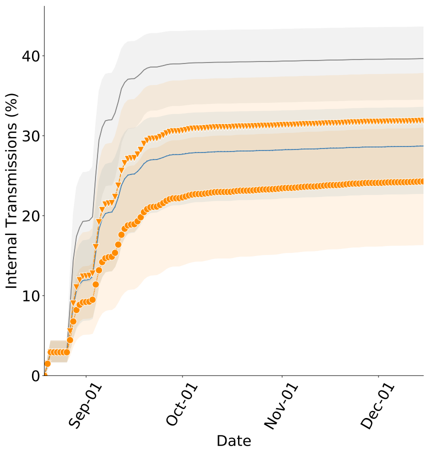

Figure S11f: S3 ($\mathsf{LC}_{\mathrm{BCen}}$)

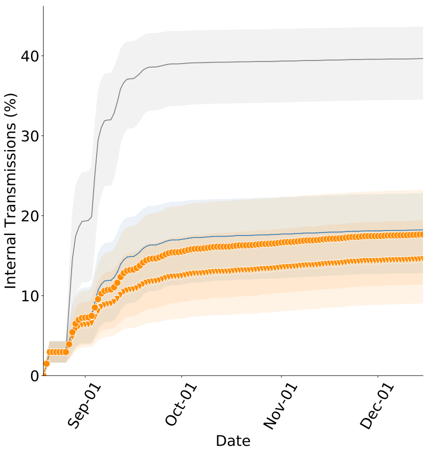

Figure S11g: S1 ($\mathsf{LC}_{\mathrm{BCen}}$)

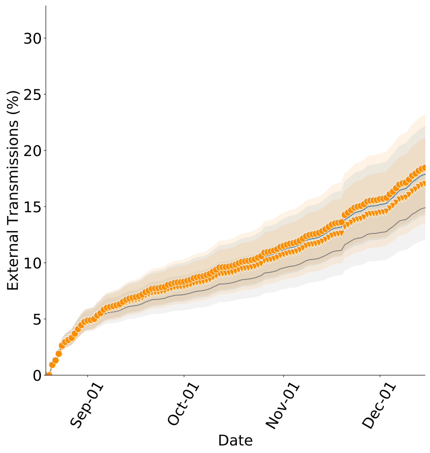

Figure S11h: S2 ($\mathsf{LC}_{\mathrm{BCen}}$)

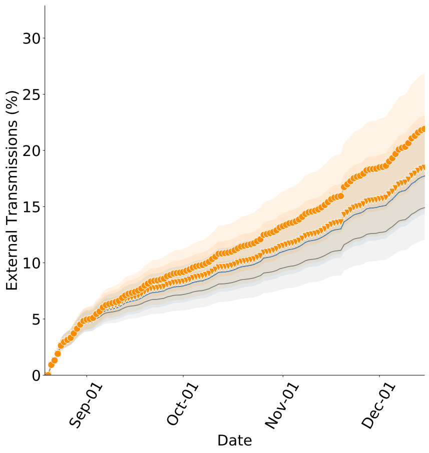

Figure S11i: S3 ($\mathsf{LC}_{\mathrm{BCen}}$)

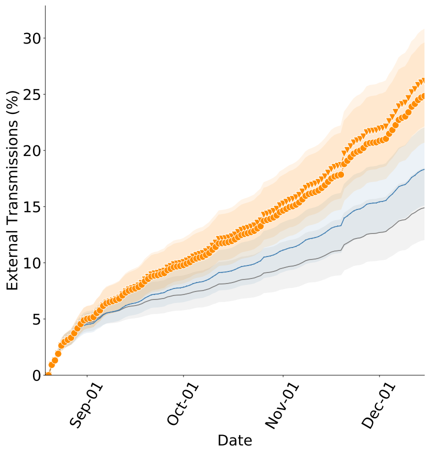

Figure S11: Cumulative infections in Fall 2019 while comparing RI and $\mathsf{LC}_{\mathrm{BCen}}$ with ABM calibrated on weeks $0-4$ of Fall 2020, GT. The bands show the 2.75^{th} and 97.25^{th} percentile. $(a-c)$ Total infections of interventions is lower than no-intervention and is lowest in the $S3$ scenario. In this scenario, the mobility budget is 69% of what it would be without interventions, and therefore the transmissions are also contained. In comparison, in Fall 2020, we saw far fewer infections which is because the mobility was 39% of that in Fall 2019. $(d-f)$ Internal transmissions are lower with $\mathsf{LC}_{\mathrm{BCen}}$ in comparison to RI, only when constrained under the exposure risk budget. $(g-i)$ External transmissions are higher with $\mathsf{LC}_{\mathrm{BCen}}$ in comparison to RI. Since internal transmission is controlled, more individuals remain susceptible to infections from outside campus.

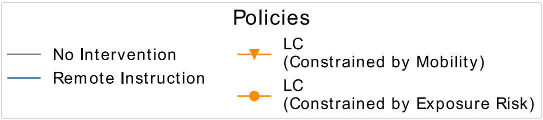

Figure S12a: S1 ($\mathsf{LC}_{\text{ECen}}$) **Figure S12b:** S2 ($\mathsf{LC}_{\text{ECen}}$) **Figure S12c:** S3 ($\mathsf{LC}_{\text{ECen}}$)

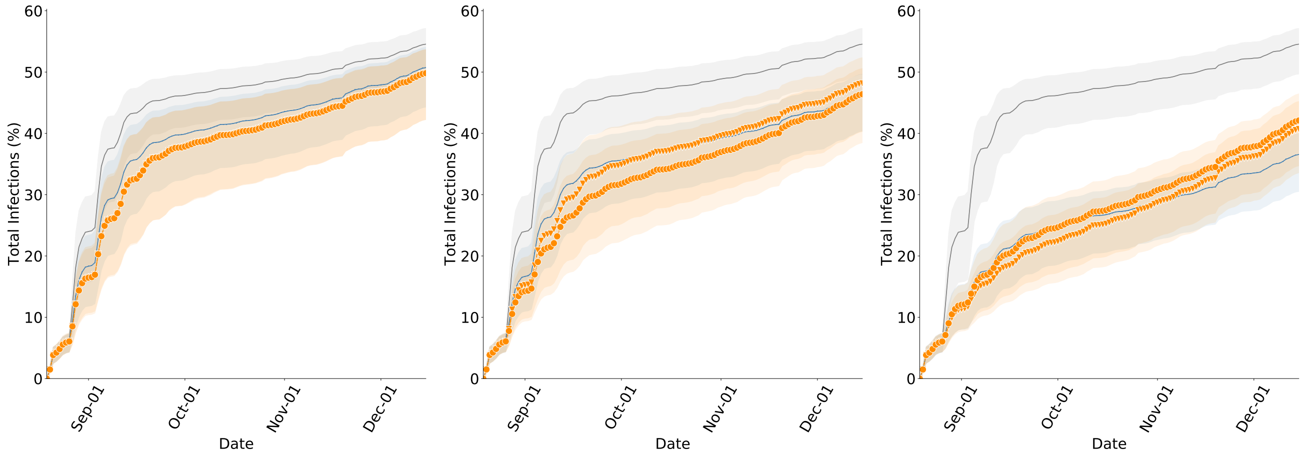

Figure S12d: S1 ($\mathsf{LC}_{\text{ECen}}$) **Figure S12e:** S2 ($\mathsf{LC}_{\text{ECen}}$) **Figure S12f:** S3 ($\mathsf{LC}_{\text{ECen}}$)

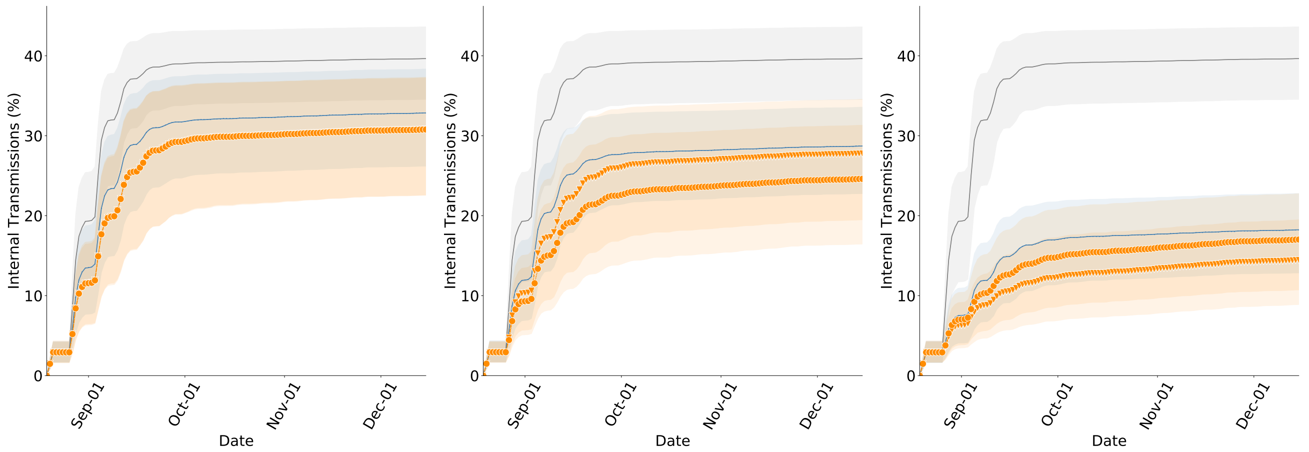

Figure S12g: S1 ($\mathsf{LC}_{\text{ECen}}$) **Figure S12h:** S2 ($\mathsf{LC}_{\text{ECen}}$) **Figure S12i:** S3 ($\mathsf{LC}_{\text{ECen}}$)

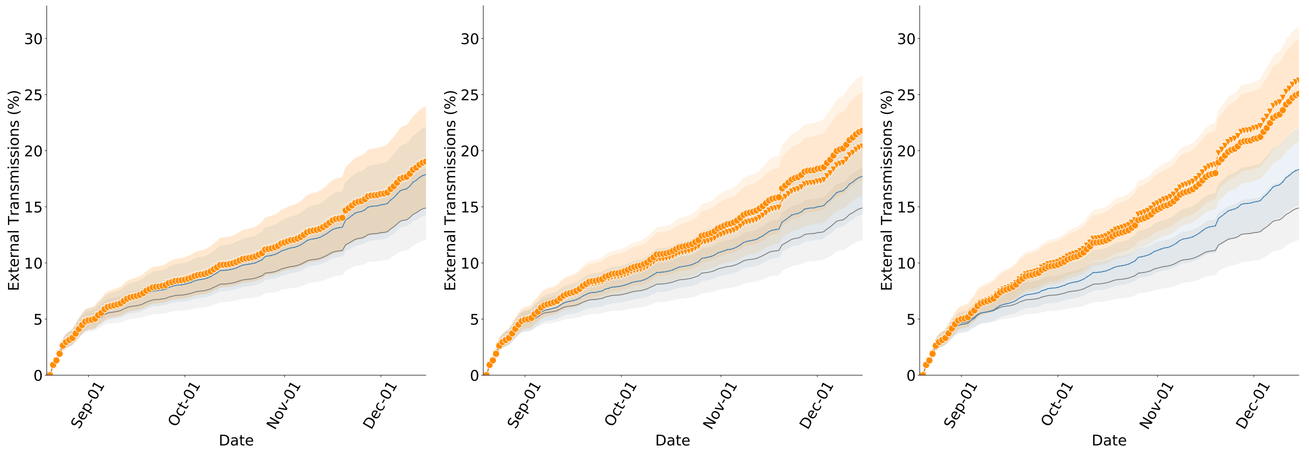

Figure S12: Cumulative infections in Fall 2019 while comparing RI and $\mathsf{LC}_{\text{ECen}}$ with ABM calibrated on weeks $0-4$ of Fall 2020, GT. The bands show the 2.75^{th} and 97.25^{th} percentile. $(a-c)$ Total infections of interventions is lower than no-intervention scenarios and is lowest in the $S3$ scenario. In this scenario, the mobility budget is 69% of what it would be without interventions, and therefore the transmissions are also contained. In comparison, in Fall 2020, we saw far fewer infections which is because the mobility was 39% of that in Fall 2019. $(d-f)$ Internal transmissions are lower with $\mathsf{LC}_{\text{ECen}}$ in comparison to RI. $(g-i)$ External transmissions are higher with $\mathsf{LC}_{\text{ECen}}$ in comparison to RI. Since internal transmission is controlled, more individuals remain susceptible to infections from outside campus.

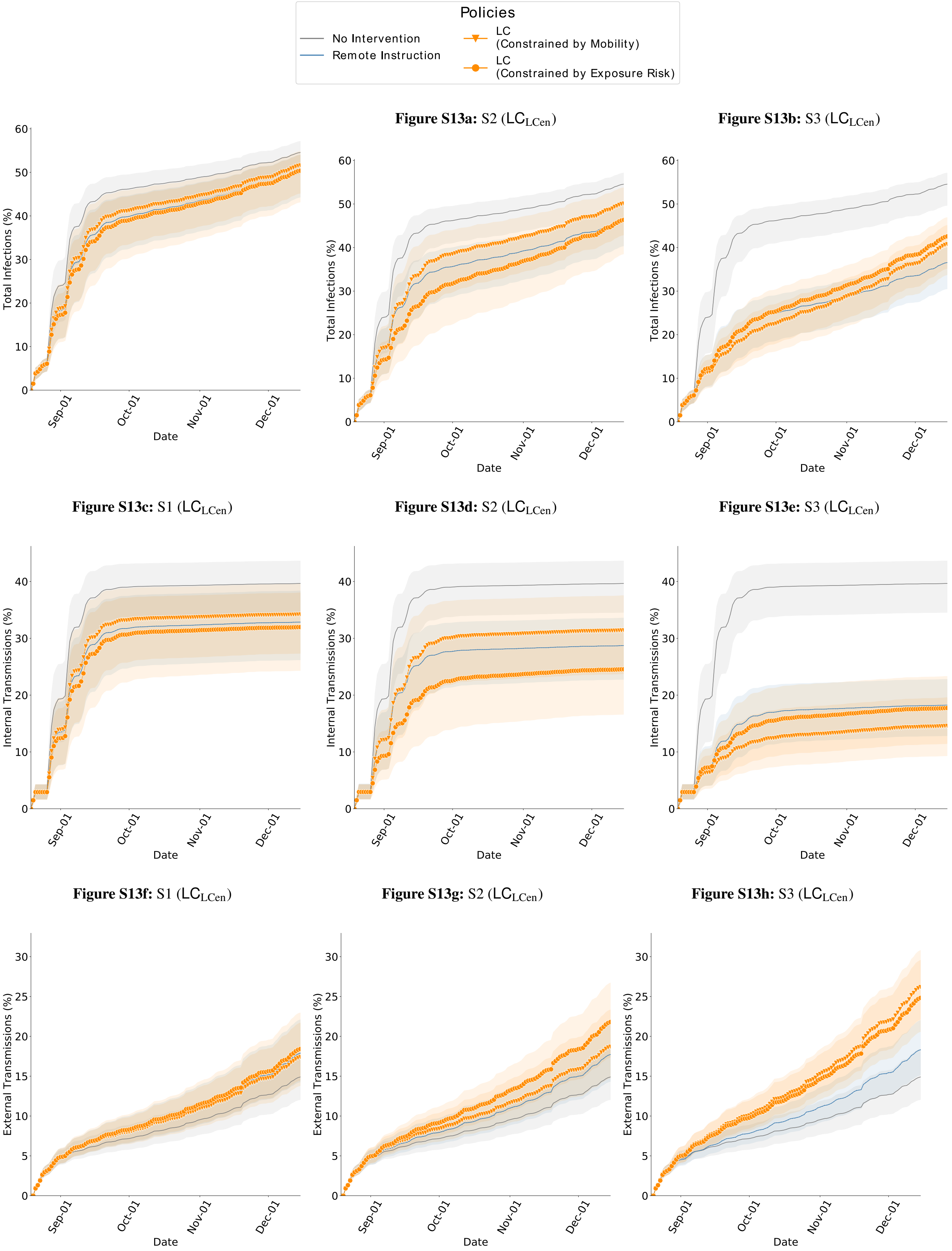

Figure S13a: S2 ($\mathsf{LC}_{\mathrm{LCen}}$)

Figure S13b: S3 ($\mathsf{LC}_{\mathrm{LCen}}$)

Figure S13c: S1 ($\mathsf{LC}_{\mathrm{LCen}}$)

Figure S13d: S2 ($\mathsf{LC}_{\mathrm{LCen}}$)

Figure S13e: S3 ($\mathsf{LC}_{\mathrm{LCen}}$)

Figure S13f: S1 ($\mathsf{LC}_{\mathrm{LCen}}$)

Figure S13g: S2 ($\mathsf{LC}_{\mathrm{LCen}}$)

Figure S13h: S3 ($\mathsf{LC}_{\mathrm{LCen}}$)

Figure S13: Cumulative infections in Fall 2019 while comparing RI and $\mathsf{LC}_{\mathrm{LCen}}$ with ABM calibrated on weeks $0-4$ of Fall 2020, GT. The bands show the 2.75^{th} and 97.25^{th} percentile. $(a-c)$ Total infections of interventions is lower than no-intervention scenarios and is lowest in the *S3* scenario. In this scenario, the mobility budget is 69% of what it would be without interventions, and therefore the transmissions are also contained. In comparison, in Fall 2020, we saw far fewer infections which is because the mobility was 39% of that in Fall 2019. $(d-f)$ Internal transmissions are lower with $\mathsf{LC}_{\mathrm{LCen}}$ in comparison to RI. $(g-i)$ External transmissions are higher with $\mathsf{LC}_{\mathrm{LCen}}$ in comparison to RI. Since internal transmission is controlled, more individuals remain susceptible to infections from outside campus.

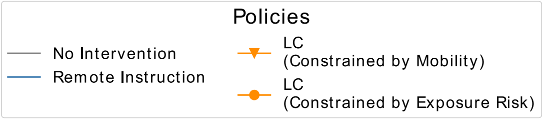

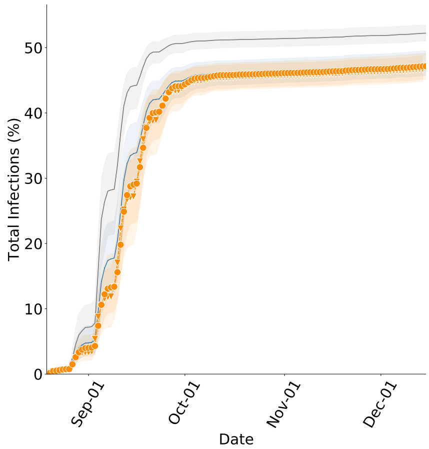

Figure S14a: S1 (ABM calibrated on weeks 5-9)

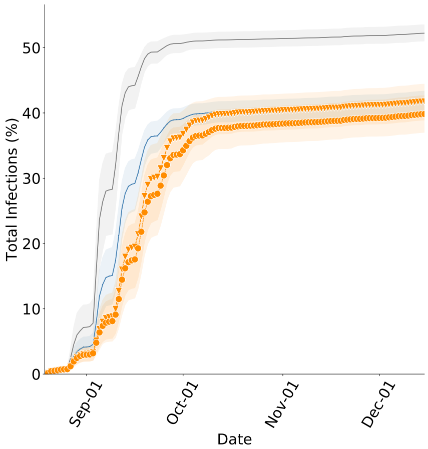

Figure S14b: S2 (ABM calibrated on weeks 5-9)

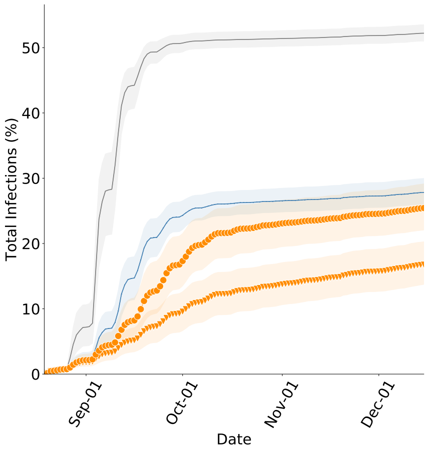

Figure S14c: S3 (ABM calibrated on weeks 5-9)

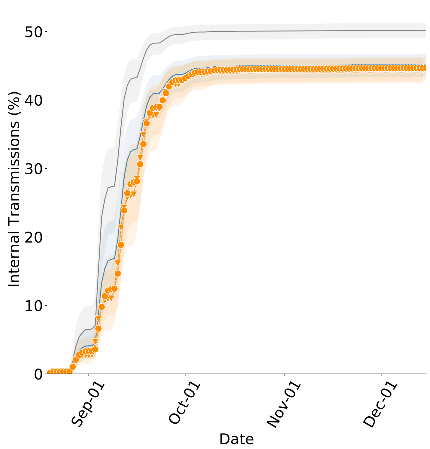

Figure S14d: S1 (ABM calibrated on weeks 5-9)

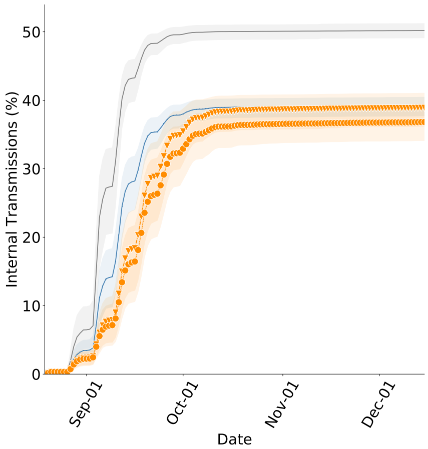

Figure S14e: S2 (ABM calibrated on weeks 5-9)

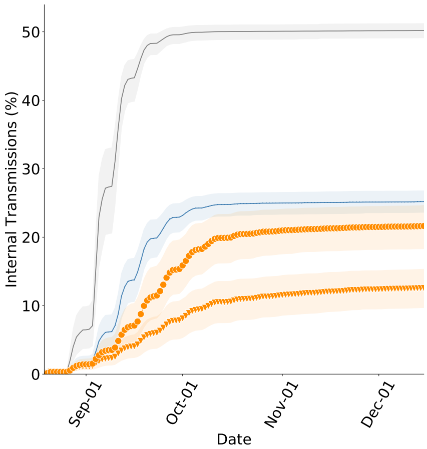

Figure S14f: S3 (ABM calibrated on weeks 5-9)

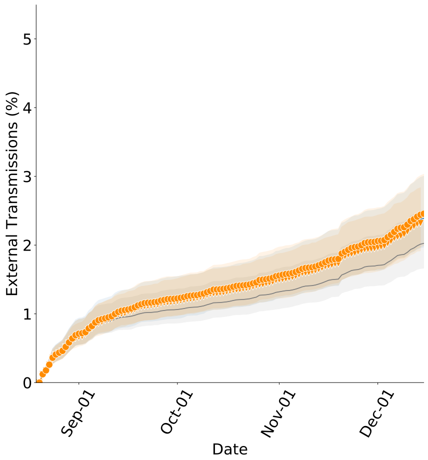

Figure S14g: S1 (ABM calibrated on weeks 5-9)

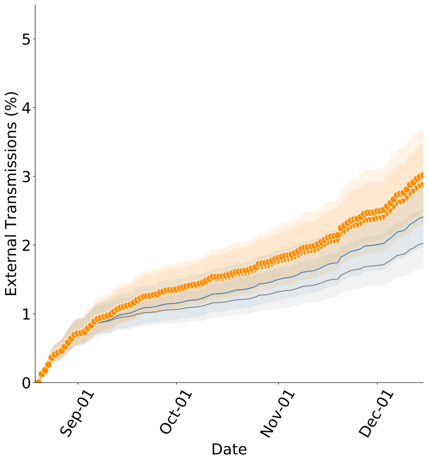

Figure S14h: S2 (ABM calibrated on weeks 5-9)

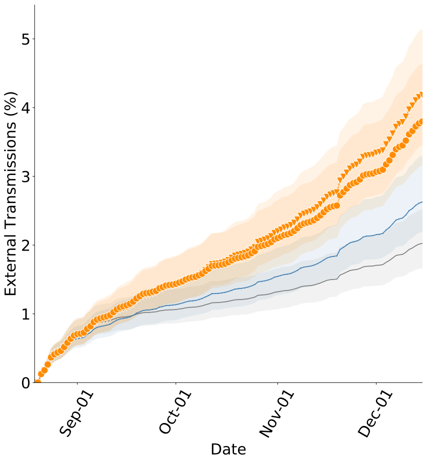

Figure S14i: S3 (ABM calibrated on weeks 5-9)

Figure S14: Cumulative infections in Fall 2019 while comparing RI and $\mathsf{LC}_{\text{PRank}}$ with ABM calibrated on weeks $5-9$ of Fall 2020, GT. The bands show the 2.75^{th} and 97.25^{th} percentile. $(a-c)$ Total infections of interventions is lower than no-intervention scenarios and is lowest in the $S3$ scenario. In this scenario, the mobility budget is 69% of what it would be without interventions, and therefore the transmissions are also contained. In comparison, in Fall 2020, we saw far fewer infections which is because the mobility was 39% of that in Fall 2019. $(d-f)$ Internal transmissions are lower with $\mathsf{LC}_{\text{PRank}}$ in comparison to RI. $(g-i)$ External transmissions are higher with $\mathsf{LC}_{\text{PRank}}$ in comparison to RI. Since internal transmission is controlled, more individuals remain susceptible to infections from outside campus.

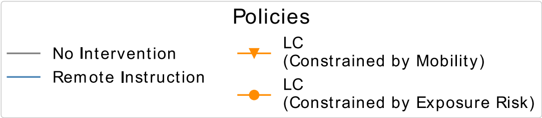

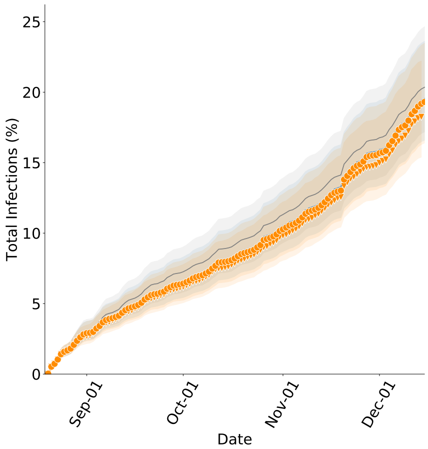

Figure S15a: S1 (ABM calibrated on weeks 10-14)

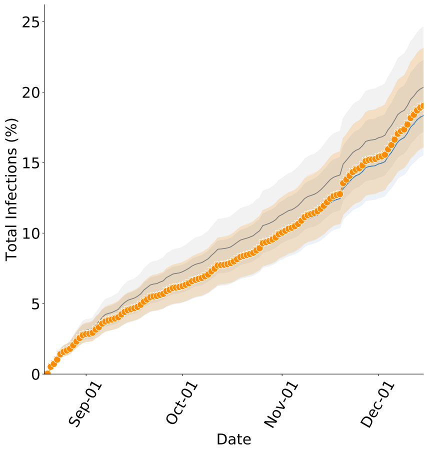

Figure S15b: S2 (ABM calibrated on weeks 10-14)

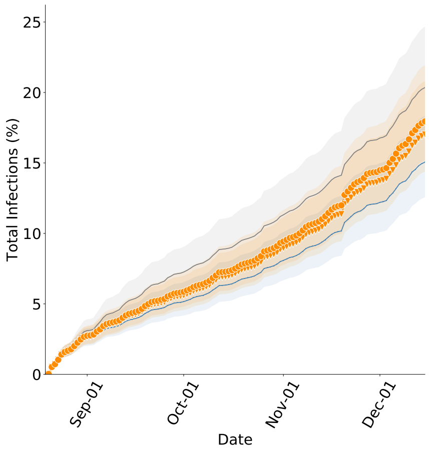

Figure S15c: S3 (ABM calibrated on weeks 10-14)

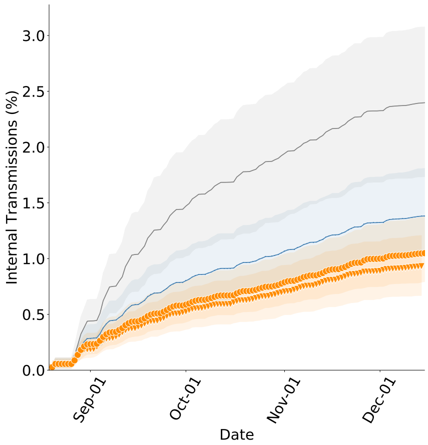

Figure S15d: S1 (ABM calibrated on weeks 10-14)

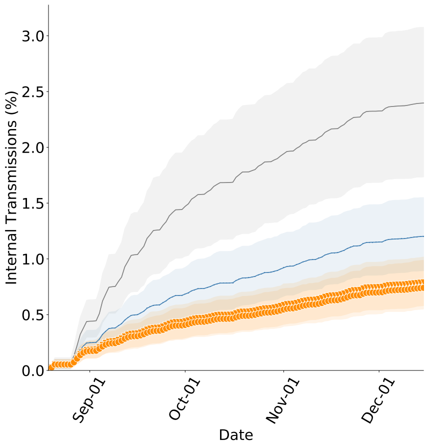

Figure S15e: S2 (ABM calibrated on weeks 10-14)

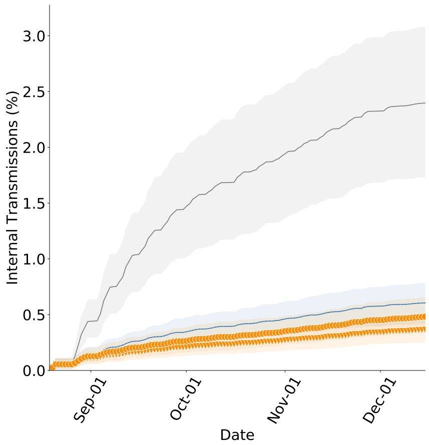

Figure S15f: S3 (ABM calibrated on weeks 10-14)

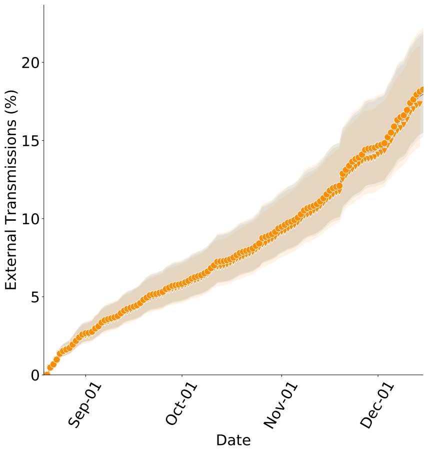

Figure S15g: S1 (ABM calibrated on weeks 10-14)

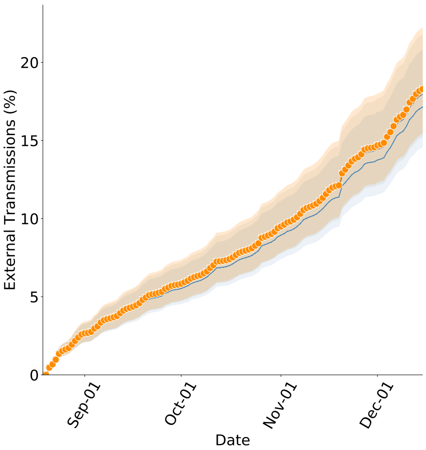

Figure S15h: S2 (ABM calibrated on weeks 10-14)

Figure S15i: S3 (ABM calibrated on weeks 10-14)

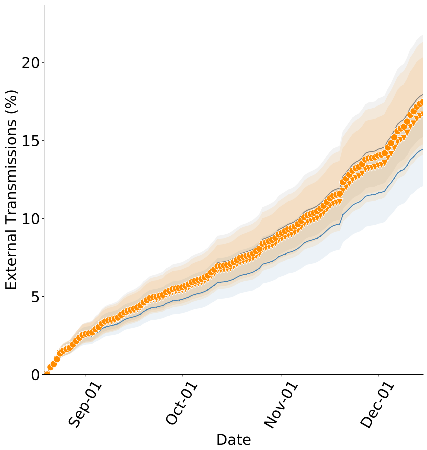

Figure S15: Cumulative infections in Fall 2019 while comparing RI and LC_{PRank} with ABM calibrated on weeks $10-14$ of Fall 2020, GT. The bands show the 2.75^{th} and 97.25^{th} percentile. $(a-c)$ Total infections of interventions is lower than no-intervention scenarios and is lowest in the *S3* scenario. In this scenario, the mobility budget is 69% of what it would be without interventions, and therefore the transmissions are also contained. In comparison, in Fall 2020, we saw far fewer infections which is because the mobility was 39% of that in Fall 2019. $(d-f)$ Internal transmissions are lower with LC_{PRank} in comparison to RI. $(g-i)$ External transmissions are higher with LC_{PRank} in comparison to RI. Since internal transmission is controlled, more individuals remain susceptible to infections from outside campus.

3 FIGURE CAPTIONS

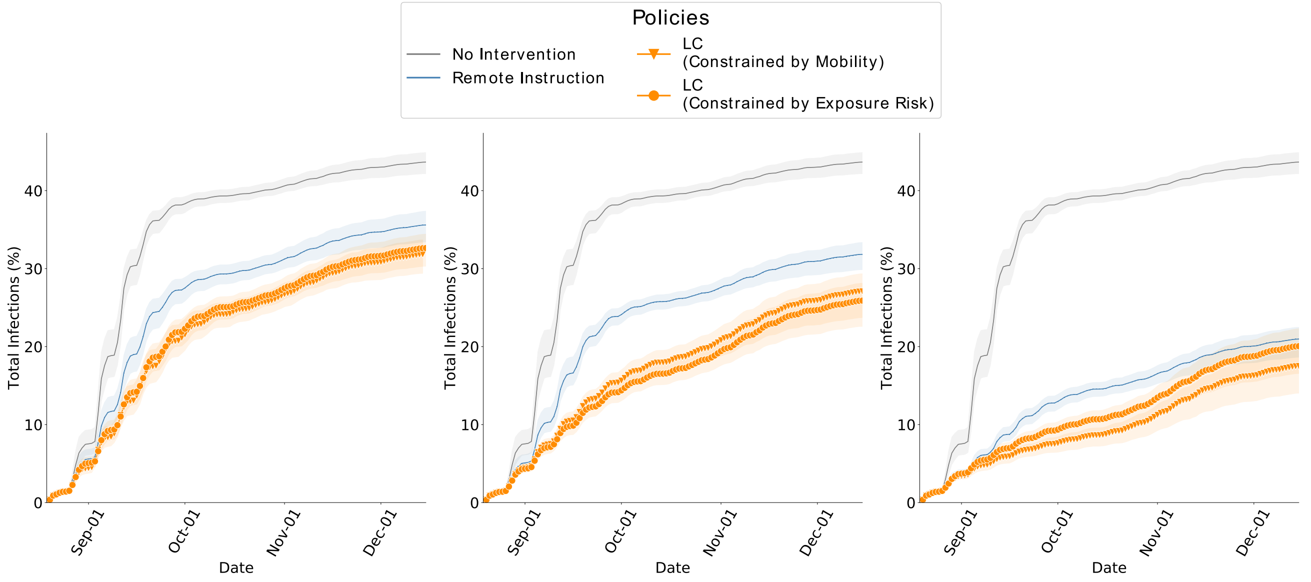

Figure S16a: S1 (ABM calibrated on UIUC data) **Figure S16b:** S2 (ABM calibrated on UIUC data) **Figure S16c:** S3 (ABM calibrated on UIUC data)

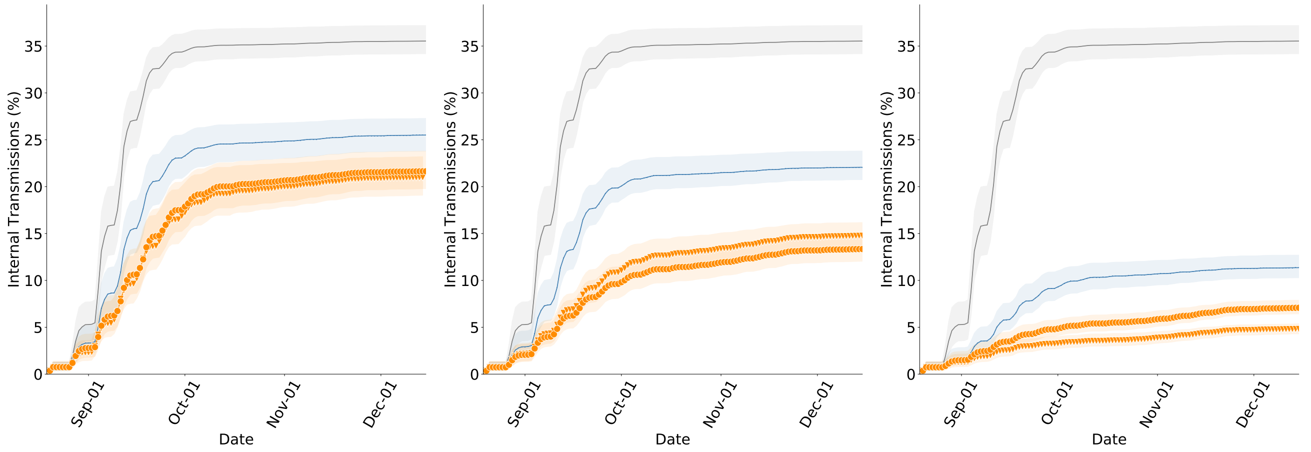

Figure S16d: S1 (ABM calibrated on UIUC data) **Figure S16e:** S2 (ABM calibrated on UIUC data) **Figure S16f:** S3 (ABM calibrated on UIUC data)

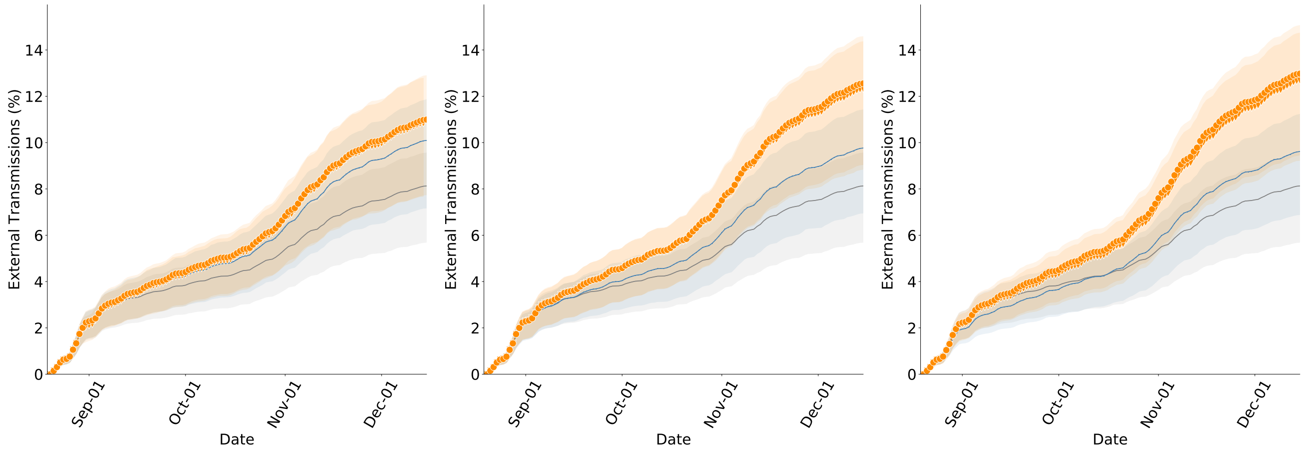

Figure S16g: S1 (ABM calibrated on UIUC data) **Figure S16h:** S2 (ABM calibrated on UIUC data) **Figure S16i:** S3 (ABM calibrated on UIUC data)

Figure S16: Cumulative infections in Fall 2019 while comparing RI and LC_{PRank} with ABM calibrated on weeks $0-4$ of Fall 2020, UIUC. The bands show the 2.75^{th} and 97.25^{th} percentile. $(a-c)$ Total infections of interventions is lower than no-intervention scenarios and is lowest in the $S3$ scenario. In this scenario, the mobility budget is 69% of what it would be without interventions, and therefore the transmissions are also contained. In comparison, in Fall 2020, we saw far fewer infections which is because the mobility was 39% of that in Fall 2019. $(d-f)$ Internal transmissions are lower with LC_{PRank} in comparison to RI. $(g-i)$ External transmissions are higher with LC_{PRank} in comparison to RI. Since internal transmission is controlled, more individuals remain susceptible to infections from outside campus.

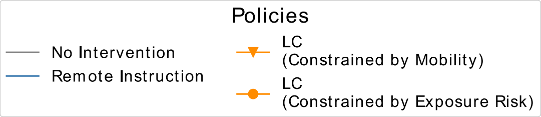

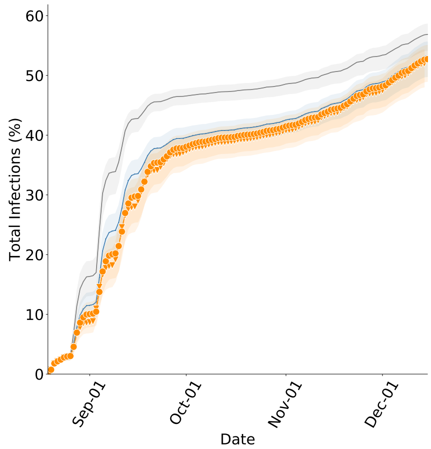

Figure S17a: S1 (ABM calibrated on Berkeley data)

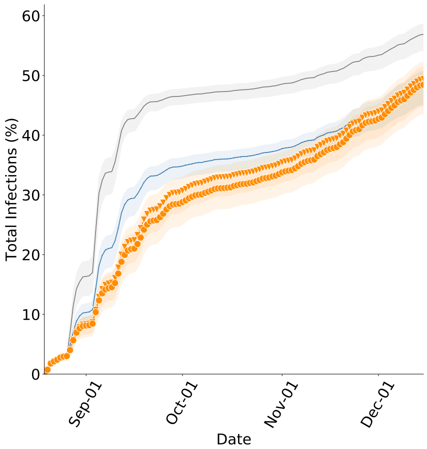

Figure S17b: S2 (ABM calibrated on Berkeley data)

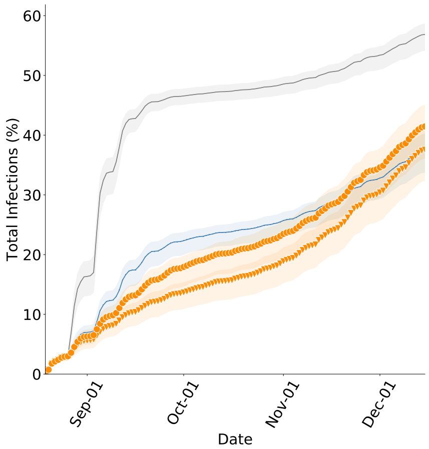

Figure S17c: S3 (ABM calibrated on Berkeley data)

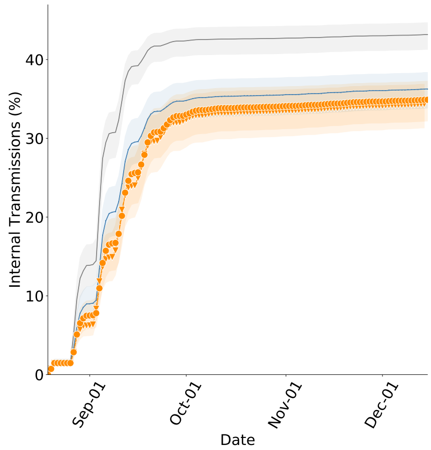

Figure S17d: S1 (ABM calibrated on Berkeley data)

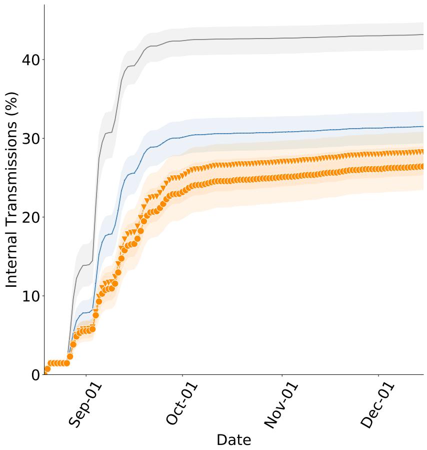

Figure S17e: S2 (ABM calibrated on Berkeley data)

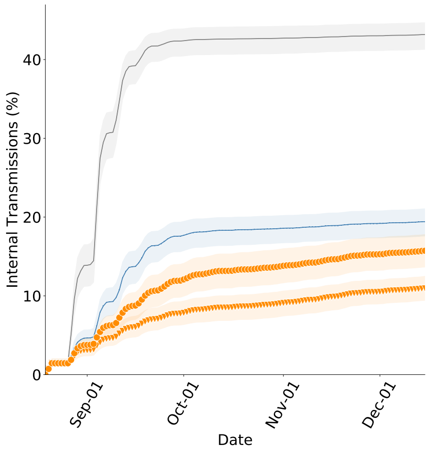

Figure S17f: S3 (ABM calibrated on Berkeley data)

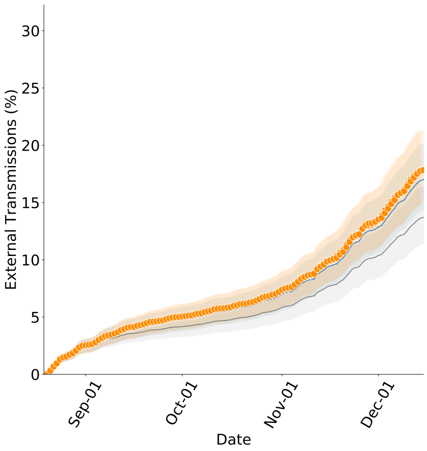

Figure S17g: S1 (ABM calibrated on Berkeley data)

Figure S17h: S2 (ABM calibrated on Berkeley data)

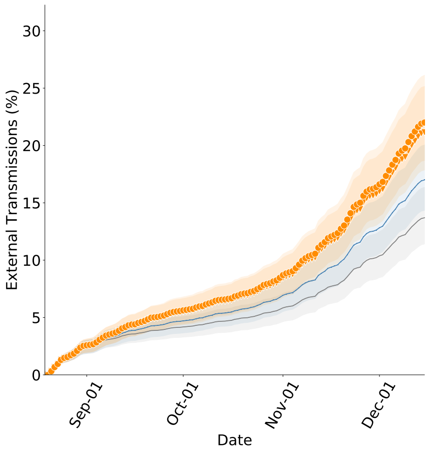

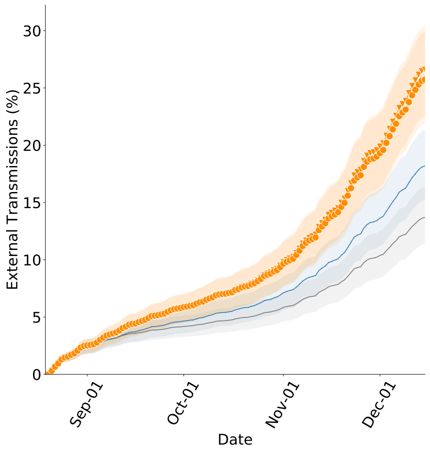

Figure S17i: S3 (ABM calibrated on Berkeley data)

Figure S17: Cumulative infections in Fall 2019 while comparing RI and LC_{PRank} with ABM calibrated on weeks $0-4$ of Fall 2020, UC Berkeley. The bands show the 2.75^{th} and 97.25^{th} percentile. $(a-c)$ Total infections of interventions is lower than no-intervention scenarios and is lowest in the $S3$ scenario. In this scenario, the mobility budget is 69% of what it would be without interventions, and therefore the transmissions are also contained. In comparison, in Fall 2020, we saw far fewer infections which is because the mobility was 39% of that in Fall 2019. $(d-f)$ Internal transmissions are lower with LC_{PRank} in comparison to RI. $(g-i)$ External transmissions are higher with LC_{PRank} in comparison to RI. Since internal transmission is controlled, more individuals remain susceptible to infections from outside campus.

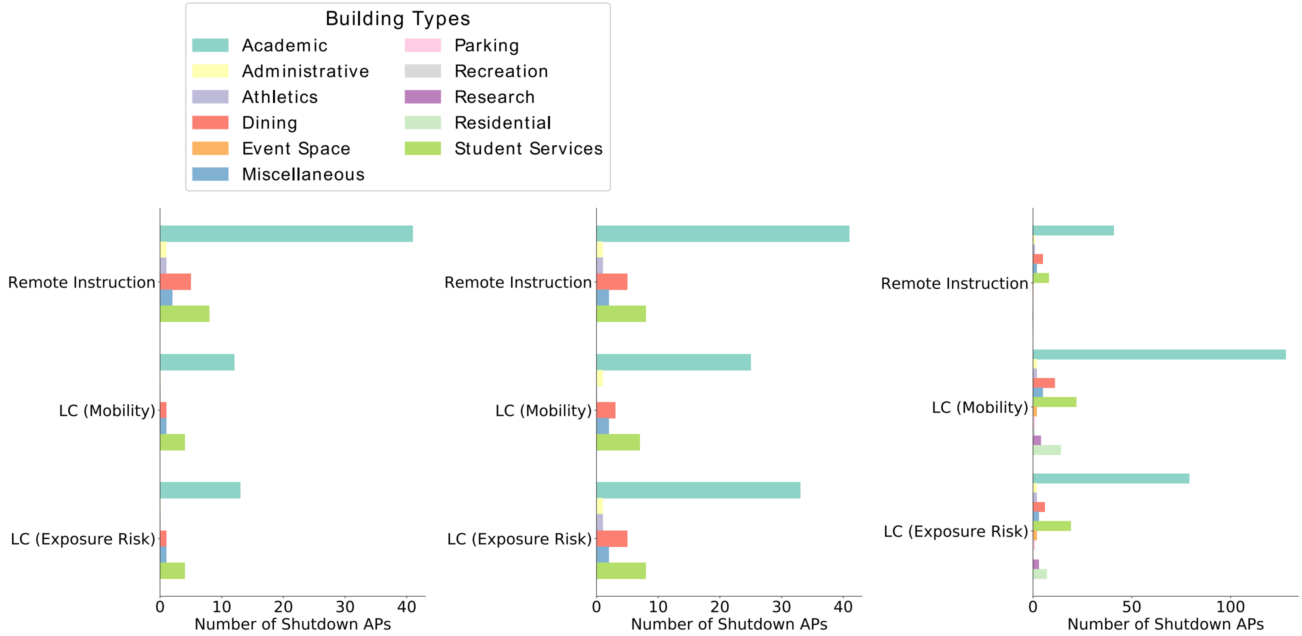

Figure S18a: S1 ($\mathsf{LC}_{\text{PRank}}$)

Figure S18b: S2 ($\mathsf{LC}_{\text{PRank}}$)

Figure S18c: S3 ($\mathsf{LC}_{\text{PRank}}$)

Figure S18d: The locations shutdown by each policy are grouped into the the general building category. The distribution of locations is different between policies, for example, in *S1* (*a*) and *S2* (*b*), LC closes fewer locations that RI. Even when targeting spaces in similar buildings, the locations are qualitatively different — RI only affects classrooms, whereas LC also closes smaller spaces like breakout rooms, reading areas and cafes. LC In *S3* (*c*) we find LC to target locations in a greater variety of buildings, but it also targets more locations to utilize the budget.

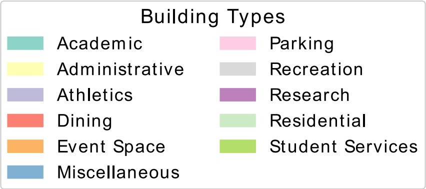

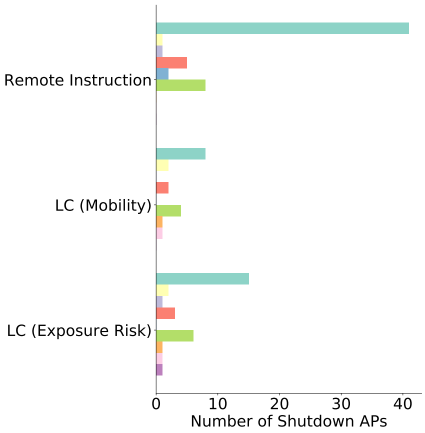

Figure S19a: S1 (LC$_{\text{BCen}}$)

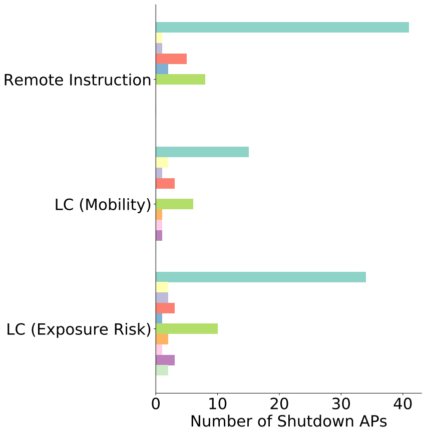

Figure S19b: S2 (LC$_{\text{BCen}}$)

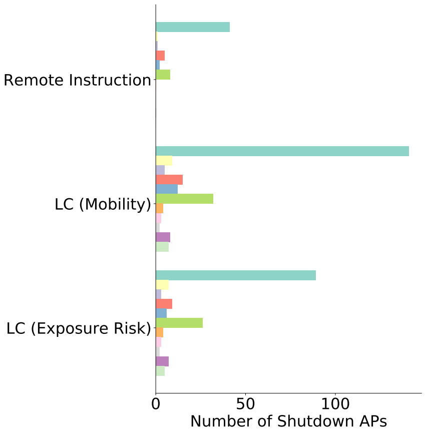

Figure S19c: S3 (LC$_{\text{BCen}}$)

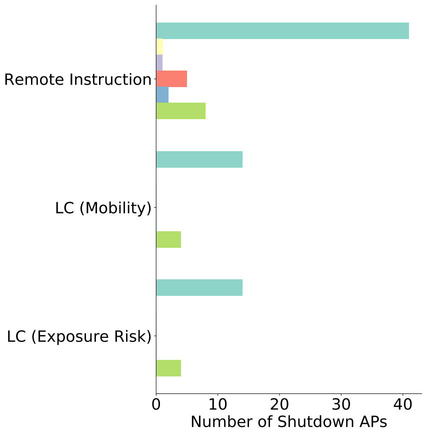

Figure S19d: S1 (LC$_{\text{ECen}}$)

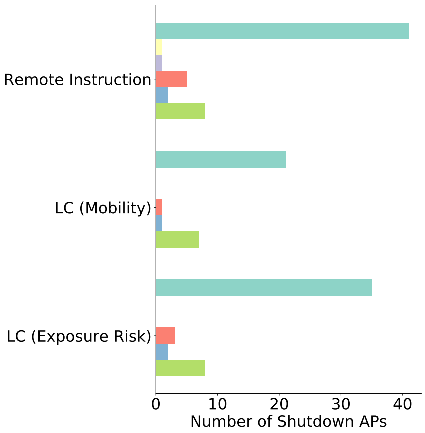

Figure S19e: S2 (LC$_{\text{ECen}}$)

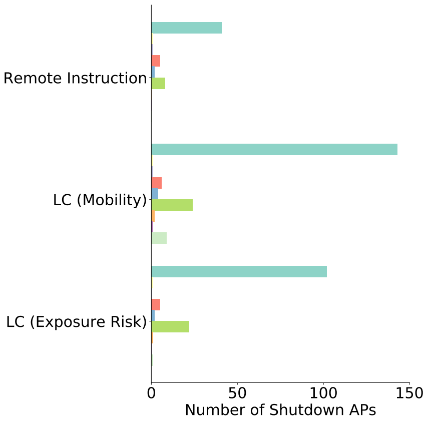

Figure S19f: S3 (LC$_{\text{ECen}}$)

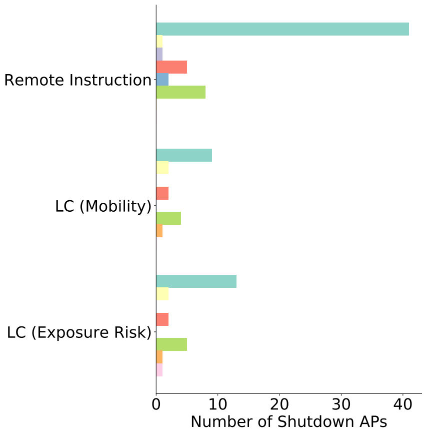

Figure S19g: S1 (LC$_{\text{LCen}}$)

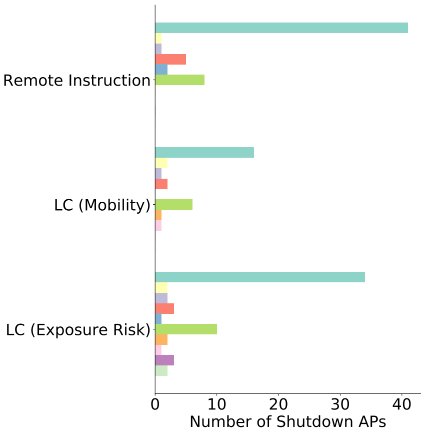

Figure S19h: S2 (LC$_{\text{LCen}}$)

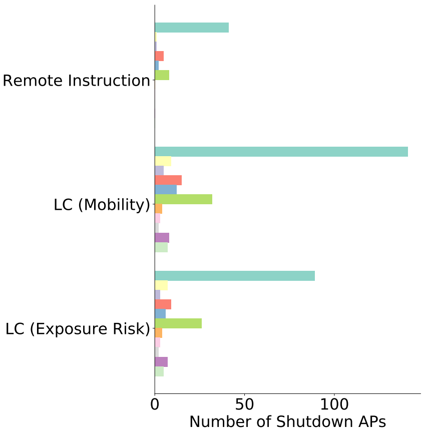

Figure S19i: S3 (LC$_{\text{LCen}}$)

Figure S19: The locations shutdown by each policy are grouped into the the general building category. The distribution of locations is different between policies, for example, in *S1* (*a*) and *S2* (*b*), LC closes fewer locations that RI. Even when targeting spaces in similar buildings, the locations are qualitatively different — RI only affects classrooms, whereas LC also closes smaller spaces like breakout rooms, reading areas and cafes. LC In *S3* (*c*) we find LC to target locations in a greater variety of buildings, but it also targets more locations to utilize the budget.

REFERENCES

[1]João Pedro Azevedo, Amer Hasan, Diana Goldemberg, Syedah Aroob Iqbal, and Koen Geven. *Simulating the potential impacts of COVID-19 school closures on schooling and learning outcomes: A set of global estimates*. The World Bank, 2020.

[2]Eugene Bagdasaryan, Griffin Berlstein, Jason Waterman, Eleanor Birrell, Nate Foster, Fred B Schneider, and Deborah Estrin. Ancile: Enhancing privacy for ubiquitous computing with use-based privacy. In *Proceedings of the 18th ACM Workshop on Privacy in the Electronic Society*, pages 111–124, 2019.

[3]Seth G Benzell, Avinash Collis, and Christos Nicolaides. Rationing social contact during the covid-19 pandemic: Transmission risk and social benefits of us locations. *Proceedings of the National Academy of Sciences*, 117(26):14642–14644, 2020.

[4]Phillip Bonacich. Some unique properties of eigenvector centrality. *Social networks*, 29(4):555–564, 2007.

[5]Molly Borowiak, Fayfay Ning, Justin Pei, Sarah Zhao, Hwai-Ray Tung, and Rick Durrett. Controlling the spread of covid-19 on college campuses. *Mathematical biosciences and engineering: MBE*, 18(1):551–563, 2020.

[6]Gabriela Arriagada Bruneau, Vincent C. Müller, and Mark S. Gilthorpe. The ethical imperatives of the covid 19 pandemic: A review from data ethics. *Veritas: Revista de Filosofía y Teología*, 46:13–35, 2020.

[7]Serina Chang, Emma Pierson, Pang Wei Koh, Jaline Gerardin, Beth Redbird, David Grusky, and Jure Leskovec. Mobility network models of covid-19 explain inequities and inform reopening. *Nature*, 589(7840):82–87, 2021.

[8]V Das Swain, H Kwon, B Saket, M Bin Morshed, K Tran, D Patel, Y Tian, J Philipose, Y Cui, T Plötz, et al. Leveraging wifi network logs to infer social interactions: A case study of academic performance and student behavior. *arXiv e-prints*, 2020.

[9]Alameda County Public Health Department. Real-time data of the impact of covid-19, 2020. Available at: `https://covid-19.acgov.org/data.page`.

[10]Champaign-Urbana Public Health Distric. Champaign-urbana covid-19 coronavirus information, 2020. Available at: `https://www.c-uphd.org/champaign-urbana-illinois-coronavirus-information.html`.

[11]Emma Dorn, Bryan Hancock, Jimmy Sarakatsannis, and Ellen Viruleg. Covid-19 and student learning in the united states: The hurt could last a lifetime. *McKinsey & Company*, 2020.

[12]Muawya Habib Sarnoub Eldaw, Mark Levene, and George Roussos. Presence analytics: making sense of human social presence within a learning environment. In *2018 IEEE/ACM 5th International Conference on Big Data Computing Applications and Technologies (BDCAT)*, pages 174–183. IEEE, 2018.

[13]Linton C Freeman. A set of measures of centrality based on betweenness. *Sociometry*, pages 35–41, 1977.

[14]Katy Gaythorpe, Natsuko Imai, Gina Cuomo-Dannenburg, Marc Baguelin, Sangeeta Bhatia, Adhiratha Boonyasiri, and A Cori. Report 8: Symptom progression of covid-19, 2020.

[15]Greg Gibson, Joshua S Weitz, Michael P Shannon, Benjamin Holton, Anton Bryksin, Brian Liu, Madeline Sieglinger, Ashley R Coenen, Conan Zhao, Stephen J Beckett, et al. Surveillance-to-diagnostic testing program for asymptomatic sars-cov-2 infections on a large, urban campus in fall 2020. *Epidemiology*, 33(2):209–216, 2021.

[16]Philip T Gressman and Jennifer R Peck. Simulating covid-19 in a university environment. *Mathematical biosciences*, 328:108436, 2020.

[17]Emily S. Gurley. Strategies to support the covid-19 response in lmics, 2020. Available at: `https://hopkinsglobalhealth.org/assets/documents/CGH_Webinar_-_Contact_Tracing_(Final_Version).pdf`.

[18]Pinar Keskinocak, Buse Eylul Oruc, Arden Baxter, John Asplund, and Nicoleta Serban. The impact of social distancing on covid19 spread: State of georgia case study. *Plos one*, 15(10):e0239798, 2020.

[19]William H Kruskal and W Allen Wallis. Use of ranks in one-criterion variance analysis. *Journal of the American statistical Association*, 47(260):583–621, 1952.

[20]Ben Lopman, Carol Y Liu, Adrien Le Guillou, Andreas Handel, Timothy L Lash, Alexander P Isakov, and Samuel M Jenness. A modeling study to inform screening and testing interventions for the control of sars-cov-2 on university campuses. *Scientific Reports*, 11(1):1–11, 2021.

[21]Christos Makridis and Jonathan Hartley. The cost of covid-19: A rough estimate of the 2020 us gdp impact, 2020.

[22]Ken IM McKinnon. Convergence of the nelder–mead simplex method to a nonstationary point. *SIAM Journal on optimization*, 9(1):148–158, 1998.

[23]Seyed M Moghadas, Meagan C Fitzpatrick, Pratha Sah, Abhishek Pandey, Affan Shoukat, Burton H Singer, and Alison P Galvani. The implications of silent transmission for the control of covid-19 outbreaks. *Proceedings of the National Academy of Sciences*, 117(30):17513–17515, 2020.

[24]Stephen J Mooney and Vikas Pejaver. Big data in public health: terminology, machine learning, and privacy. *Annual review of public health*, 39:95–112, 2018.

[25]Mark EJ Newman. Scientific collaboration networks. ii. shortest paths, weighted networks, and centrality. *Physical review E*, 64(1):016132, 2001.

[26]University of Illinois at Urbana-Champaign. On-campus covid-19 testing, 2020. Available at: `https://covid19.illinois.edu/on-campus-covid-19-testing-data-dashboard/`.

[27]Georgia Department of Public Health. Georgia department of public health daily status report, 2020. Available at: `https://dph.georgia.gov/covid-19-daily-status-report`.

[28]Georgia Institute of Technology. Georgia tech launches campus coronavirus testing, 2020. Available at: `https://health.gatech.edu/coronavirus/testing-launched`.

[29]Lawrence Page, Sergey Brin, Rajeev Motwani, and Terry Winograd. The pagerank citation ranking: Bringing order to the web. Technical report, Stanford InfoLab, 1999.

[30]Betty Pfefferbaum and Carol S North. Mental health and the covid-19 pandemic. *New England Journal of Medicine*, 383(6):510–512, 2020.

[31]Andreas Pfitzmann and Marit Hansen. A terminology for talking about privacy by data minimization: Anonymity, unlinkability, undetectability, unobservability, pseudonymity, and identity management, 2010.

[32]John H Stone, Matthew J Frigault, Naomi J Serling-Boyd, Ana D Fernandes, Liam Harvey, Andrea S Foulkes, Nora K Horick, Brian C Healy, Ruta Shah, Ana Maria Bensaci, et al. Efficacy of tocilizumab in patients hospitalized with covid-19. *New England Journal of Medicine*, 383(24):2333–2344, 2020.

[33]Berkeley University of California. Coronavirus dashboard testing, 2020. Available at: `https://coronavirus.berkeley.edu/dashboard/`.

[34]Srinivasan Venkatramanan, Adam Sadilek, Arindam Fadikar, Christopher L Barrett, Matthew Biggerstaff, Jiangzhuo Chen, Xerxes Dotiwalla, Paul Eastham, Bryant Gipson, Dave Higdon, et al. Forecasting influenza activity using machine-learned mobility map. *Nature Communications*, 12(1):1–12, 2021.

[35]Jessa Liying Wang and Michael C Loui. Privacy and ethical issues in location-based tracking systems. In *2009 IEEE International Symposium on Technology and Society*, pages 1–4. IEEE, 2009.

[36]Shweta Ware, Chaoqun Yue, Reynaldo Morillo, Jin Lu, Chao Shang, Jayesh Kamath, Athanasios Bamis, Jinbo Bi, Alexander Russell, and Bing Wang. Large-scale automatic depression screening using meta-data from wifi infrastructure. *Proceedings of the ACM on Interactive, Mobile, Wearable and Ubiquitous Technologies*, 2(4):1–27, 2018.

[37]Sarah Watson, Shawn Hubler, Danielle Ivory, and Robert Gebeloff. A new front in america's pandemic: College towns, 2020. Available at: `https://www.nytimes.com/2020/09/06/us/colleges-coronavirus-students.html`.

[38]Kim A Weeden and Ben Cornwell. The small-world network of college classes: implications for epidemic spread on a university campus. *Sociological science*, 7:222–241, 2020.

[39]Bryan Wilder, Marie Charpignon, Jackson A Killian, Han-Ching Ou, Aditya Mate, Shahin Jabbari, Andrew Perrault, Angel N Desai, Milind Tambe, and Maimuna S Majumder. Modeling between-population variation in covid-19 dynamics in hubei, lombardy, and new york city. *Proceedings of the National Academy of Sciences*, 117(41):25904–25910, 2020.
